# Sources of Variation in the Spectral Slope of the Sleep EEG

**DOI:** 10.1523/ENEURO.0094-22.2022

**Published:** 2022-09-21

**Authors:** Nataliia Kozhemiako, Dimitris Mylonas, Jen Q. Pan, Michael J. Prerau, Susan Redline, Shaun M. Purcell

**Affiliations:** 1 Department of Psychiatry, Brigham & Women’s Hospital, Harvard Medical School, Boston, MA 02115; 2 Department of Psychiatry, Massachusetts General Hospital, Harvard Medical School, Boston, MA 02114; 3 Stanley Center for Psychiatric Research, Broad Institute, Cambridge, MA 02142; 4 Department of Medicine, Brigham & Women’s Hospital, Harvard Medical School, Boston, MA 02115

**Keywords:** 1/*f* slope, developmental changes, electroencephalography, individual variability, spectral slope

## Abstract

The 1/*f* spectral slope of the electroencephalogram (EEG) estimated in the γ frequency range has been proposed as an arousal marker that differentiates wake, nonrapid eye movement (NREM) sleep, and rapid eye movement (REM) sleep. Here, we sought to replicate and extend these findings in a large sample, providing a comprehensive characterization of how slope changes with age, sex, and its test-retest reliability as well as potential confounds that could affect the slope estimation. We used 10,255 whole-night polysomnograms (PSGs) from the National Sleep Research Resource (NSRR). All preprocessing steps were performed using an open-source Luna package and the spectral slope was estimated by fitting log-log linear regression models on the absolute power from 30 to 45 Hz separately for wake, NREM, and REM stages. We confirmed that the mean spectral slope grows steeper going from wake to NREM to REM sleep. We found that the choice of mastoid referencing scheme modulated the extent to which electromyogenic, or electrocardiographic artifacts were likely to bias 30- to 45-Hz slope estimates, as well as other sources of technical, device-specific bias. Nonetheless, within individuals, slope estimates were relatively stable over time. Both cross-sectionally and longitudinal, slopes tended to become shallower with increasing age, particularly for REM sleep; males tended to show flatter slopes than females across all states. Our findings support that spectral slope can be a valuable arousal marker for both clinical and research endeavors but also underscore the importance of considering interindividual variation and multiple methodological aspects related to its estimation.

## Significance Statement

In a large sample, we validate the electroencephalogram (EEG) spectral slope as a practical and scalable neurobiological marker of cortical arousal across wake, nonrapid eye movement (NREM), and rapid eye movement (REM) sleep states. We report sources of intraindividual and interindividual variability, including changes across the lifespan. The slope during REM (when it is steepest, consistent with higher cortical inhibition) showed the greatest age-related flattening, suggesting that sleep-based biomarkers may be particularly sensitive to age-related physiological change, including cognitive decline. We also highlight critical methodological and sample issues. Our findings support alternative parameterizations of the EEG as being valuable for clinical and research endeavors, but also underscore the importance of accounting for interindividual and technical sources of variation.

## Introduction

Building on prior work (Pereda et al., 1998; Shen et al., 2003; Freeman and Zhai, 2009; Gao et al., 2017; Miskovic et al., 2019), Lendner et al. (2020) recently reported an electrophysiological marker of arousal level in humans: the 1/*f* spectral slope of the electroencephalogram (EEG) estimated in the range of 30–45 Hz. When calculated so as to avoid frequencies with strong oscillatory components (such as spindle activity during N2 sleep), the linear slope of the log-log power spectrum (the so-called “spectral noise” exponent) can be interpreted as an index of the aperiodic, scale-free component that reflects aggregated neural dynamics (Voytek and Knight, 2015). Furthermore, it has been suggested that the spectral slope might reflect the ratio of neural excitation and inhibition (E/I balance; Gao, 2016; Gao et al., 2017; Lombardi et al., 2017). Lendner and colleagues found significantly steeper slopes (consistent with greater cortical inhibition) in rapid eye movement (REM) compared with non-REM (NREM) sleep, and in NREM sleep compared with wake, concluding that the spectral slope represents a biomarker of human arousal, that might have applications in intraoperative neuromonitoring or automatic sleep stage classification, for example.

Here, we initially sought to replicate Lendner and colleagues’ core result concerning scalp EEG spectral slope and sleep state, leveraging a collection of over 15,000 whole-night polysomnograms (PSGs) from the National Sleep Research Resource (NSRR). Whereas Lendner and colleagues’ analyses were restricted to a small sample (*N *=* *20 for the primary scalp sleep EEG dataset) albeit one complemented with additional imaging and intracranial EEG recordings, our analyses of multiple, diverse cohorts were intended to provide high statistical power, robustness and generalizability across populations. Althogh only two central scalp EEG channels (C3 and C4) were available accross studies in our sample, Lendner and colleagues reported broad effects across the scalp including central sites and source localization was not a focus of this report. To address potential role of spectral slope in automated sleep staging, we also asked whether the spectral slope predicted sleep state (epoch by epoch) with greater accuracy than traditional (e.g., band power) metrics.

Lendner and colleagues employed various checks for potential methodological confounds, including referencing scheme and analytic approach. Although they did not look at contralateral mastoid (CM) referencing (i.e., in the present context, C4-M1 and C3-M2), slope estimates were broadly consistent across the different referencing schemes considered [linked mastoid (LM), average, Laplacian and bipolar]. We initially adopted CM referencing, which is recommended for clinical studies by the American Academy of Sleep Medicine (AASM); further, the Sleep Heart Health Study (SHHS) dataset (total *N *=* *8444 nights on 5793 individuals) was recorded with contralateral references hardwired and so could not be re-referenced offline. Lendner and colleagues briefly considered potential confounding because of muscle activity: controlling for the spectral slope derived from the chin electromyogram (EMG), they reported that this did not alter results. Nonetheless, that the EMG is (1) of orders of magnitude higher amplitude than the EEG, (2) exhibits a broad frequency spectrum that overlaps the EEG spectrum, particularly at higher (>30 Hz) frequencies (Goncharova et al., 2003), and (3) varies markedly between wake, NREM, and REM (Jacobson et al., 1964), collectively make this a serious concern that should always be addressed. In particular, as mastoid electrodes are sensitive to neck muscle EMG and cardiac activity (via blood flow in the carotid arteries), here we considered possible indicators of confounding because of non-neural sources, with attention to the choice of mastoid referencing scheme.

Beyond our primary replication attempt, to robustly establish mean slope differences between states, to more fully characterize spectral slope distributions we evaluated its variability as a function of sleep state, as well as state-specific covariation between spectral slope, power and coherence. Using model-based simulation, we considered whether changes in the spectral slope alone (assuming a strict power law model) could account for these other characteristics.

Finally, as well as within-individual, between-state phenomena, we investigated between-individual, within-state changes in the spectral slope. Previous reports have suggested that the spectral slope varies between individuals in systematic and physiologically relevant ways, for example, flattening with age (Feinberg et al., 1984; Voytek et al., 2015; Dave et al., 2018; Donoghue et al., 2020; Bódizs et al., 2021; Schaworonkow and Voytek, 2021; Hill et al., 2022). As NSRR cohorts included males and females from ∼5 to ∼97 years of age (and a sample of individuals with a repeated sleep study, performed years later), we also tested whether the spectral slope showed age-related flattening and sex differences, and whether these effects varied by sleep state.

## Materials and Methods

### PSG data

All PSG data were as previously reported (Purcell et al., 2017). Briefly, we combined PSG and demographic data on 11,630 individuals aged 4–97 years from the NSRR. All data were collected as part of research protocols that were approved by local institutional review boards; written, informed consent was obtained from each individual before participation. The majority of individuals were from community-based samples, although two studies recruited participants for sleep apnea. A subset (*N *=* *4079) had a second PSG, typically administered five or six years after the first. We accessed European Data Format (EDF) files and annotation files (indicating manually scored sleep states in 30-s epochs as well as arousals and respiratory events). All studies used AASM staging conventions, except the SHHS, which used R&K: here, NREM3 and NREM4 were collapsed to a single N3 state, for consistency with the other studies.

Cohorts ordered by average age are as follows: CHAT (children), CCSHS (adolescents), CFS (wide range, but predominantly adolescents and middle-aged adults), SHHS (middle-aged adults), MrOS (elderly males), and SOF (elderly females). We generally stratified analyses by study to control for possible technical and measurement differences as well as the effects of aging. The CHAT baseline cohort contained children with an apnea hypopnea index of 2–30, randomized to one of two trial arms; the CHAT nonrandomized group contained children screened for the trial but not randomized, either because of unwillingness or apnea hypopnea indices that did not meet inclusion criteria. The CHAT follow-up cohort was collected six to seven months after intervention with adenotonsillectomy or watchful waiting.

All EEG analyses were based on two central electrodes, initially re-referenced to the CM (C3-M2 and C4-M1). For specific analyses, we alternatively re-referenced to the average mastoid ([M1+M2]/2) denoted here as C3-LM and C4-LM (for “linked mastoid”); we also derived two bipolar channels: C3-C4 and M1-M2. The chin EMG channel was derived from left and right submentalis electrodes; the ECG channel was derived from left and right arm electrodes. All physiological signals (EEG, EMG, and ECG) were resampled to 128 Hz.

### Epoch-level artifact removal

Many recordings contained extended periods of gross artifact at the beginning and ends, typically scored as “wake.” Although most NSRR studies did not have explicit “lights on/off” annotations, these leading/trailing wake periods typically included “lights on” periods, i.e., with the participant not in bed. To alleviate this issue of excessive artifact during wake, we removed all leading/trailing wake epochs, meaning that all “wake” data reported below occurred during the sleep period (i.e., after sleep onset and before the final sleep epoch). We further removed any epoch containing a manually annotated arousal, apnea, or hypopnea. All subsequent analyses were state-dependent; we only selected epochs of a given state if they were flanked by at least one other similar epoch on both sides, to exclude transitional/unstable periods of sleep. Of note, these criteria meant that a nontrivial proportion of the overall sample was excluded, and in particular the studies of younger individuals [because of their relatively low rate of extended wake time after sleep onset (WASO)].

We next applied epoch-wise normalization to each channel, subtracting its median value. EEG channels were further high-pass filtered at 2 Hz using a zero-phase Kaiser window FIR (transition bandwidth 2 Hz, ripple 0.01). Separately for each stage (N1, N2, N3, R, or W), we removed epochs for which either C3-M2 or C4-M1 had (1) a flat signal spanning >10% of the epoch, (2) a clipped signal spanning >10% of the epoch, (3) >10% of the epoch exceeding 100 μV, (4) >1% of the epoch exceeding 250 μV, or (5) a maximum absolute amplitude <5 μV. Based on EEG and chin EMG channels, we further removed epochs that were statistical outliers (±3 SD units from the mean) for at least one of those channels for one or more Hjorth parameter (activity, mobility, complexity; Hjorth, 1970), by comparing that epoch to the mean across all epochs of that stage and channel. Hjorth-based statistical outlier removal was performed twice. Finally, we removed epochs that were flanked by multiple already-masked epochs (where the masking may have been because of any of the above reasons). Specifically, we required that at least three of five flanking epochs passed quality check (QC), either for the five preceding or the five following; we further required that retained epochs were immediately flanked by at least one other QC-passing epochs.

Collectively, this procedure was intentionally conservative, weighing specificity over sensitivity with respect to selecting clean and homogeneous epochs for the final analytic samples. Because we wanted to select a single set of criteria to be applied across all studies and stages, this means that studies had a nontrivial proportion of epochs removed (especially for younger individuals who did not meet the WASO criterion). Future analyses of these datasets focused only on the spectral slope during sleep could of course achieve larger sample sizes by ignoring the extent of wake in each study.

### Spectral power analysis

We used the Welch method to estimate power spectra per epoch, using 4-s segments each with 50% (2 s) overlap, applying a Tukey (cosine-tapered) window with α = 0.5, yielding a spectral resolution of 0.25 Hz. We also estimated spectral band power using the following definitions: slow (0.5–1 Hz), δ (1–4 Hz), θ (4–8 Hz), α (8–11 Hz), σ (11–15 Hz), β (15–30 Hz), and γ (for this analysis, defined as 30–45 Hz to match the interval in which the spectral slope was estimated). In sensitivity analyses, we compared spectral slopes estimated from the Welch spectra to those based on multitaper analysis; here we applied 29 tapers and set the time half bandwidth product to 15 to achieve a frequency resolution of 1 Hz for a 30-s epoch. The resulting spectral slopes correlated *r *>* *0.99 and so we based all final analyses on the Welch method.

As artifacts (e.g., corresponding to electrical line noise at 60 Hz and subharmonics) can lead to sharp peaks in power spectra which may bias the estimation of spectral slopes, we quantified the extent of spectral “peakedness” within the 30- to 45-Hz interval. Specifically, we detrended log-transformed power spectra (assuming a linear *x*-axis frequency scale) and applied a median filter (using an 11 point = 2.5-Hz window) to smooth the detrended spectra. Labeling the detrended spectra *D* and the detrended and smoothed spectra *S*, we quantified peakedness as the kurtosis of the *D* – *S* difference spectra. Power spectra with strong peaks will show more leptokurtic (long tailed) distributions.

As well as summarizing mean power per individual/channel/stage by averaging over all epochs, we estimated the SDs of epoch-level metrics, to facilitate the analyses of within-individual variability in spectral power.

### Spectral slope estimation

Following Lendner and colleagues, to estimate the spectral slope, we extracted the absolute power from 30 to 45 Hz (here 61 values) and fit log-log linear regression models, which they showed to be appropriate for this interval of the EEG spectrum, being free of strong oscillatory activity. To reduce the impact of line noise and other artifacts that might induce sharp spectral peaks, we removed outlier data points (i.e., particular frequency/power pairs): specifically, before running the main regression, we fit an initial model and removed points that had residuals >2 SD units from the mean. Also, because we estimated slope only over a relatively restricted interval (30–45 Hz), we did not consider the potential issue that can arise with different representations of lower versus higher frequencies because of the log-scaling of linearly uniform frequencies. For example, there is only a 1.45-fold difference in the log-scale intervals from 30 to 31 Hz compared with 44–45 Hz; in contrast, there is >20-fold difference between 30- to 31-Hz and 1- to 2-Hz intervals, implying that if we had looked at a broader frequency range, this issue would be more apparent.

Power spectra were estimated individually for each epoch. To derive the spectral slope, we averaged log-scaled spectra, and then calculated a single slope estimate. As a sensitivity analysis, we also calculated two other estimates: (1) averaging untransformed spectra, then taking the log and estimating the slope; alternatively; (2) averaging slopes calculated per epoch on the log-transformed spectra. That is, the three approaches can be labeled: *slope(mean(log(X)))*, *slope(log(mean(X)))* and *mean(slope(log(X)))*, respectively, where *X* are the epoch-level power spectra. An advantage of the third approach is that it also provides a convenient estimate of the variability in slope across epochs. We expect qualitatively similar results from all approaches, although the second approach may be more sensitive to outlier epochs not otherwise filtered out. Work on the resting state eyes open/closed alpha rhythm differences suggested that log-transforming power at the epoch level before averaging was optimal in that context (Smulders et al., 2018). Based on an initial analysis in the CFS cohort, all three metrics were effectively equivalent, both in terms of correlation with each other (*r > *0.95) and the magnitude of tests of mean differences between stages (data not shown). The one exception was that *slope(log(mean(X)))* was less correlated with the other two metrics during wake (here *r* ∼0.7), which presumably reflects a greater sensitivity to noisy/outlier epochs that occur more often during wake. As noted, our final analyses were based on *slope(mean(log(X)))*, although we do not expect any substantive differences in results if these alternatives were used.

### Individual-level outlier rejection

After obtaining stage-specific slope and spectral power estimates for all individuals, we removed individuals from all primary analyses if they either (1) did not have a sufficient duration of epochs for key stages, or (2) had excessive outliers for key metrics. Specifically, we required at least 5 min (10 epochs) of nominally artifact-free data for W, N2, and R (as key analyses were based on these stages, and some individuals had relatively short N1 and/or N3 durations). We then removed individuals who were outliers (4 SD units) for any of the following metrics (applied sequentially such that latter filters only consider the set of currently of included individuals): γ band power for C4-M1 or C3-M2 for W, N2 or R; γ band power for a mastoid-mastoid derivation M1-M2 for W, N2 or R; the kurtosis (spectral peakedness) measure for C4-M1, C3-M2 or M1-M2 for W, N2 or R; the mean and SD of the spectral slope estimates for C4-M1 or C3-M2 for W, N2 or R; for C4-M1 or C3-M2, the difference in spectral slope between R and N2, between R and W, or between N2 and W; finally, the mean and SD of the EMG spectral slope. For the second round of analyses based on LM channels, the C3-LM and C4-LM were additionally included in the above exclusions, as well as C3-M2 and C4-M1.

Our primary analyses are presented with these relatively stringent individual-level outlier rejection criteria; however, all key results were qualitatively similar if less stringent (or if no) outlier rejection procedures were applied instead (data not shown).

### State classification

To classify individual epochs in the CFS dataset based on the spectral slope (or other metric), we used linear discriminant analysis (LDA; as implemented in “MASS” R library) together with leave-one-out cross validation. Classification was performed in a pairwise fashion for W versus R, W versus N2, and R versus N2. Accuracy was computed as the measure of classifier performance. Individuals with <20 epochs for any of the states were excluded from this analysis, resulting in a final sample of 311 individuals. Following Lendner and colleagues, to ensure that the chance level equated to 50% accuracy, we randomly downsampled one state to ensure the same number of epochs for both states. To reduce variability, we repeated the previous step 50 times, averaging to obtain the final accuracy per individual. Within individual, spectral slopes and log-scaled power estimates were z-scored before LDA. Matched pair *t* tests were used to compare accuracies based on spectral slopes versus band power.

### 1/*f* model-based parameterization of individual differences in power spectra

We directly simulated power spectra for *N *=* *5000 individuals, initially in the form PSD(*f*) = *A*/*f^α^* + *e*, where *A* and *α* were random variables for the spectral intercept and exponent (slope β = –*α*), respectively. Following others (Gao, 2016; Podvalny et al., 2015), we also extended the basic power law equation model in three ways. First, whereas a strict power law implies the slope rotates around 1 Hz (i.e., log(1) = 0), we allowed an alternate point of rotation, *f_r_* to be specified, such that log PSD(*f*) = log *A* + *α* log (*f*/*f_r_*) + *e*. Second, following Gao (2016), we allowed for an additional “flat” spectrum *C*, as a single, fixed term added to all points of the spectrum, such that log PSD(*f*) = log (exp (log *A* + *α* log (*f*/*f_r_*) + *e*) + *C*) . Third, we allowed the magnitude of the influence of *α* to vary across the power spectrum, by weighting its contribution by a frequency-dependent sigmoid weight function, *w*(*f*)* *=* *1/ (1 + exp (– (*f* – *w_f_*) /*w_s_*)), meaning that at frequency *w_f_* the impact of the slope defined by the random variable *α* is 50% of its full value; it rises to 100% at higher frequencies, and at lower frequencies, falls to toward 0%. The scaling factor *w_s_* determined the sharpness of transition. An independent random variable α* was defined similarly to *α* and applied with weight 1-*w*(*f*). In this way, although the overall, marginal properties of the spectra were broadly similar, this parameterization allowed for variation in the slope in one frequency range (e.g., >20 Hz) to be independent of variation in the slope at lower frequencies.

After generating a sample of *N *=* *5000 independent spectra (0.5–50 Hz in 0.5-Hz increments) under a particular set of parameter values, we used log-log linear regression to estimate the realized spectral slope in the 30- to 45-Hz frequency range, for each individual spectrum. In order to replicate the findings shown in Results, Relationships between the 30- to 45-Hz spectral slope and power, we then calculated the Pearson correlation coefficient between the slope and power value (for power from 0.5 to 50 Hz) across all spectra; based on a median split of the estimated spectral slope, we also plotted the mean power spectra for individuals with “steep” versus “shallow” slopes.

### Data and code accessibility

EEG signal analysis was performed with Luna (v0.26), an open-source C/C++ package for the analysis of sleep signal data (http://zzz.bwh.harvard.edu/luna/); all PSG data are available via the NSRR (http://sleepdata.org). The Luna script used for the primary analyses is available on request. Luna code was run on a cluster of compute nodes with Linux operating system.

## Results

We analyzed 15,709 whole-night PSGs on 11,630 individuals (Table 1), 4079 of whom had a second PSG, from the NSRR. This is the same sample (comprising ten cohorts from six distinct studies) as previously described in a study of sleep spindle activity (Purcell et al., 2017). Three cohorts were pediatric, six were of middle to late adulthood, and one was a family-based study with a wide age range. Other than CHAT, all cohorts were observational and not undergoing sleep or other interventions.

**Table 1:** Cohort characteristics

Study	Label	Sample *N*	% female	Mean age	Min. age	Max. age	DOI
Childhood Adenotonsillectomy Trial (baseline)	CHAT(BL)	453	52%	6.6	5	10	doi.org/10.25822/d68d-8g03
Childhood Adenotonsillectomy Trial (nonrandomized)	CHAT(NR)	779	53%	7.1	5	10	doi.org/10.25822/d68d-8g03
Childhood Adenotonsillectomy Trial (follow-up)	CHAT(FU)	407	51%	7.1	5	10	doi.org/10.25822/d68d-8g03
Cleveland Children’s Sleep and Health Study	CCSHS	515	50%	17.7	16	20	doi.org/10.25822/cg2n-4y91
Cleveland Family Study	CFS	730	55%	41.4	7	89	doi.org/10.25822/jmyx-mz90
Sleep Heart Health Study (wave 1)	SHHS1	5793	52%	63.1	39	90	doi.org/10.25822/ghy8-ks59
Sleep Heart Health Study (wave 2)	SHHS2	2647	54%	67.6	44	90	doi.org/10.25822/ghy8-ks59
Osteoporotic Fractures in Men Study (wave 1)	MrOS1	2907	0%	76.4	67	96	doi.org/10.25822/kc27-0425
Osteoporotic Fractures in Men Study (wave 2)	MrOS2	1025	0%	81.1	73	97	doi.org/10.25822/kc27-0425
Study of Osteoporotic Fractures	SOF	453	100%	82.9	75	95	doi.org/10.25822/e1cf-rx65

All data are available via the NSRR (http://sleepdata.org). CHAT(FU), SHHS2, and MrOS2 cohorts contained repeated PSGs performed on subsets of CHAT(BL), SHHS1, and MrOS1. For post-QC sample description, see Extended Data Table 1-1.

10.1523/ENEURO.0094-22.2022.t1-1 Extended Data Table 1-1 **Pre-QC and post-QC sample sizes and durations of sleep state by cohort.** For each cohort, the average duration of each state, following the initial round of QC (based on removing individuals without sufficient duration (at least 10 minutes) after performing various epoch-level exclusions (QC1), e.g. only retaining epochs flanked by similarly staged epochs, rejecting epochs with annotated arousals or respiratory events, as well as signal outliers, etc. The second round of QC (QC2) excluded individuals based upon statistical properties of the derived metrics (e.g. power, slope). See **Methods** for details. These procedures were collectively designed to be stringent: that they removed large proportions of some cohorts for the final analysis more reflects the choices of QC rather than inherent issues with the data (i.e. many childhood recordings were removed due to low rates of WASO, as we excluded leading and trailing wake epochs from all recordings, but required all studies to have sufficient duration of wake as well as sleep epochs). No SHHS individuals were retained in the linked mastoid dataset, as it was not possible to re-reference the hardwired contralateral mastoid channels; also see ‘Technical factors in the SHHS datasets’ in Results for other technical issues encountered in the SHHS cohorts. Download Table 1-1, DOC file.

After stringent exclusions and QCs (see Materials and Methods and Extended Data Table 1-1), the final analytic sample comprised 10,255 nights on 7312 individuals. Most primary analyses were based on the first recording from each individual, pooling CHAT baseline and nonrandomized samples, yielding six independent cohorts. Cohorts containing repeated recordings (CHAT follow-up, SHHS2, and MrOS2) were used in the longitudinal analyses, paired with their respective baseline cohort.

### Initial analyses of spectral power and slope stratified by sleep state

**Figure 1.**
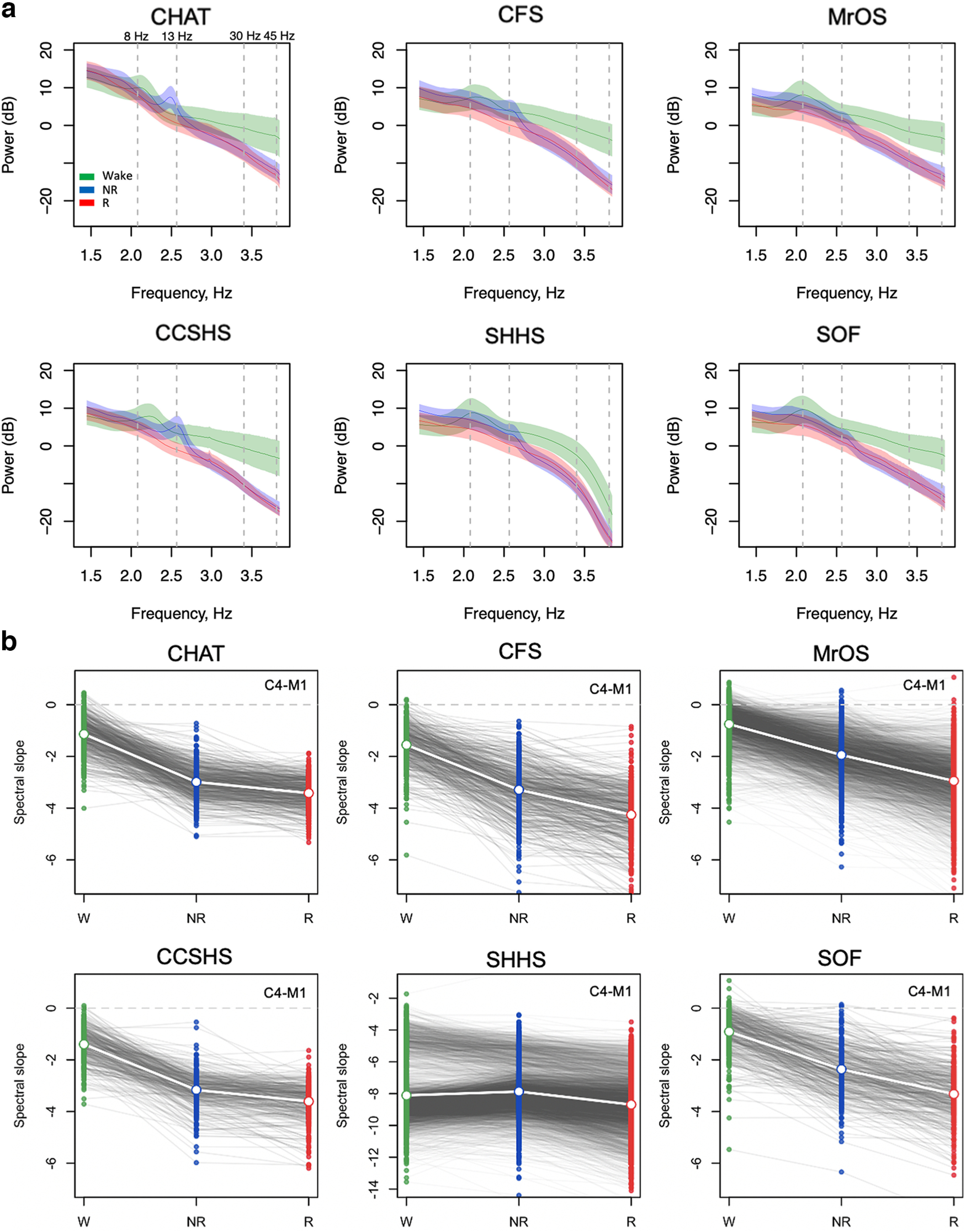
Spectral power and slopes based on contra-lateral mastoid referencing. For the channel C4-M1 (linked mastoid referencing, see Extended Data Fig. 1-2; for other channels/reference schemes, see Extended Data Fig. 1-3), (***a***) mean log power spectra (5–46 Hz) as a function of log frequency by sleep state (wake, NREM, and REM) and cohort with shading illustrating the SDs. Dashed vertical lines at 8 and 13 Hz indicate typical modes of oscillatory activity during wake (α rhythms) and N2 (spindles); dashed lines at 30 and 45 Hz indicate the interval within which the spectral slope was estimated. ***b***, Estimated spectral slopes for the set of independent first-wave individuals (CHAT baseline and nonrandomized samples pooled). Gray lines connect the three values for each individual. Note the different scaling of the *y*-axis for SHHS1 versus the five other datasets (for details, see Extended Data Fig. 1-1). Green, blue, and red indicate wake, NREM, and REM, respectively. See Table 1 for sample size details.

Primary analyses were based on a three-level classification: wake, N2 sleep and REM sleep. As more individuals had a sufficient duration of N2 sleep following extensive QC (at least 10 epochs), compared with N1 or N3 (Extended Data Table 1-1), we focused specifically on N2 sleep for all primary NREM analyses. (As results for N1 and N3 were broadly equivalent to those for N2, Figures and tables only show N2 results but refer to it as “NREM sleep” generically.) We first estimated each study’s mean log-scaled EEG power spectra (C4-M1) during wake, NREM, and REM (Fig. 1*a*). Perhaps most notably, both SHHS studies showed divergent, supralinear mean spectra across all states, especially >30 Hz. On further investigation, we identified a set of technical issues specific to the SHHS that impacted the high-frequency EEG (described below, Technical factors in the SHHS datasets, and Extended Data Fig. 1-1). Beyond this and as expected, wake power spectra exhibited characteristic ∼8-Hz α peaks across all cohorts, whereas N2 spectra exhibited characteristic σ-band peaks. The childhood CHAT samples (green lines) had more pronounced spindle peaks at slower frequencies (e.g., 11 Hz) and, across all states, higher power at lower frequencies.

10.1523/ENEURO.0094-22.2022.f1-1 Extended Data Figure 1-1 **Spectral slopes in SHHS**. a) Histograms of W spectral slopes for C_3_-M2 and C_4_-M1, for both wave 1 and 2, which indicate a bimodal distribution for C_4_-M1 only. b) Mean spectral slopes (separately for C_3_-M2 and C_4_-M1 in wave 1 and 2) stratified by the ID of the physical recording device (headbox). The same units and IDs were preserved across waves 1 and 2 (albeit with fewer individuals/devices used, as well as a handful of new devices introduced for wave 2). The devices that were outliers for C_4_-M1 in wave 2 were also outliers in wave 1. Download Figure 1-1, TIF file.

**Figure 2.**
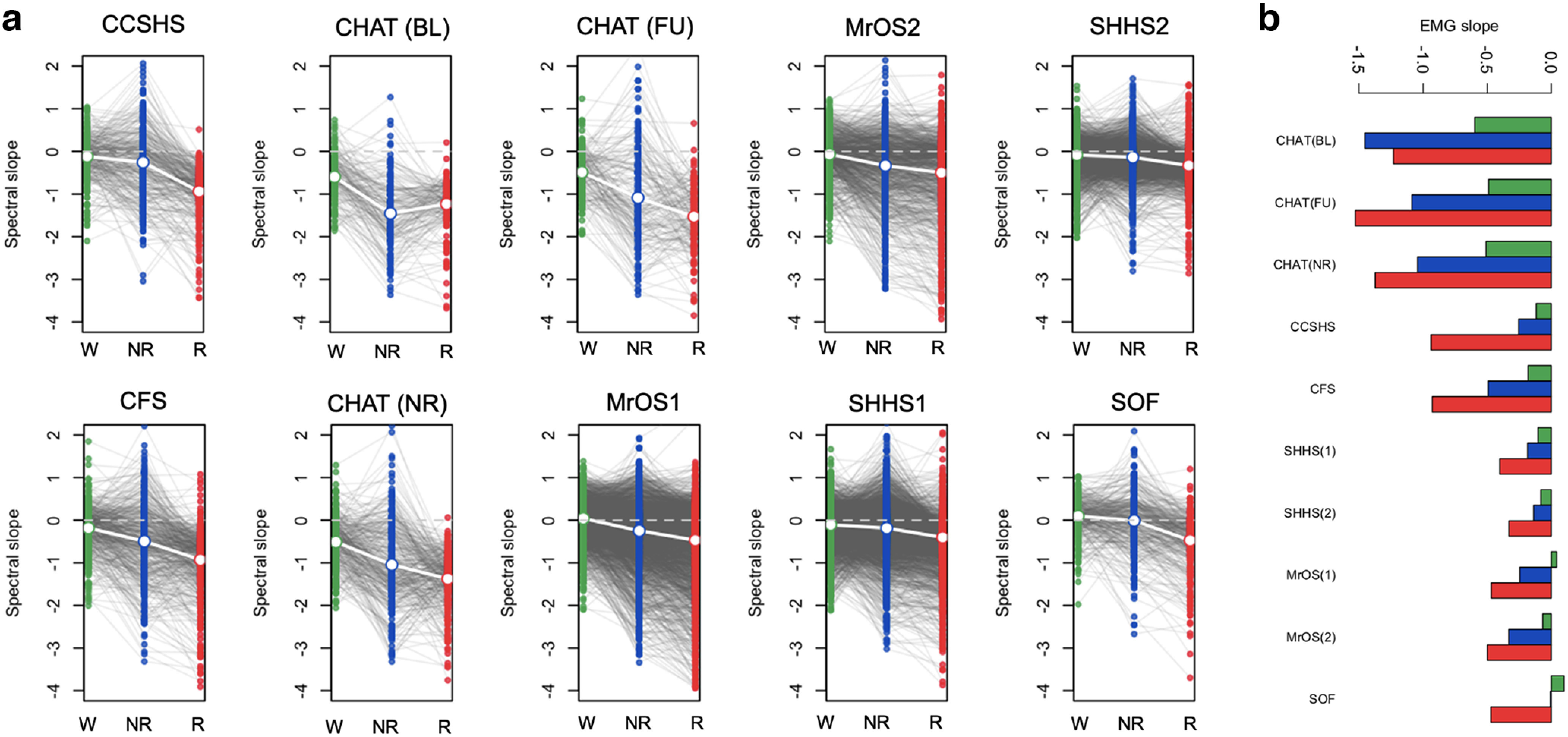
Spectral slope of the EMG. ***a***, Distributions of EMG 30- to 45-Hz spectral slopes, stratified by state and cohort (for statistical information, see Extended Data Fig. 2-1). ***b***, The mean EMG slopes (identical to those in panel ***a***) plotted differently, to emphasize the age-related flattening (for the slope associations with sex and BMI, see Extended Data Figs. 2-4, 2-5, 2-6). Green, blue, and red indicate wake, NREM, and REM, respectively. Also see Extended Data Figures 2-2, 2-3, 2-7, and 2-8 for correlations between EMG and EEG slopes and EMG-EEG coherence. See Extended Data Figure 2-9 for the illustration of reference choice and ECG artifacts.

10.1523/ENEURO.0094-22.2022.f1-2 Extended Data Figure 1-2 **EEG spectral slopes (CM-referenced dataset)**. See legend for **Figure 1** for details: similar methods were applied to generate these plots, but here listed for both central channels, and separately for all ten samples. Green, blue and red indicate wake, NREM and REM respectively. Download Figure 1-2, TIF file.

**Figure 3.**
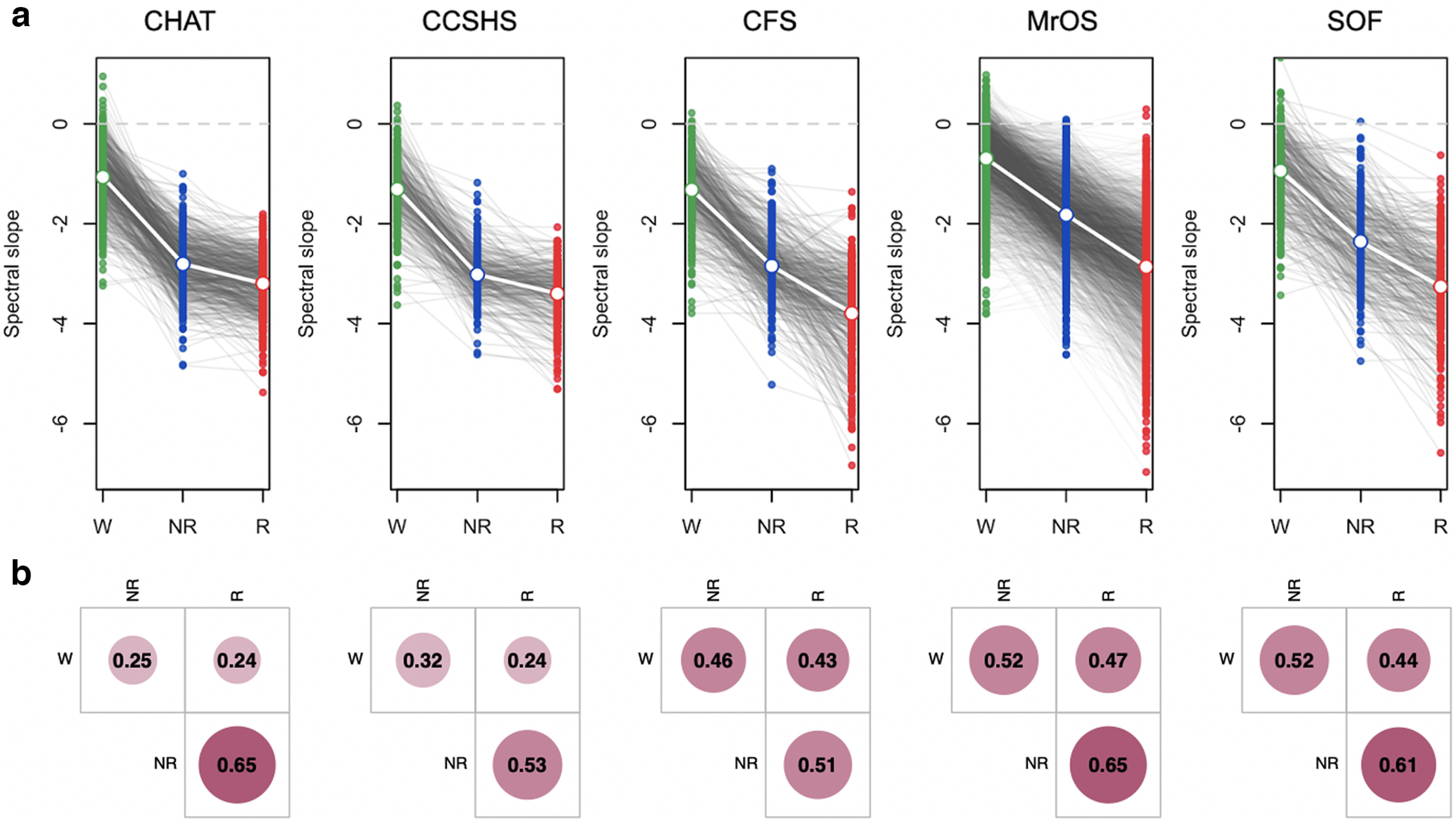
Spectral power based on LM referencing, excluding SHHS. ***a***, Plots as for Figure 1*b* but based on the LM referencing scheme (all state differences, matched pair *t* test *p *<* *10^−15^) at C3LM (for C4LM channel, see Extended Data Figs. 3-1, 3-2). ***b***, Pearson correlation coefficients in slope between the three sleep states considered (all *p *<* *10^−5^) at C3LM (for C4LM channel, see Extended Data Fig. 3-3). Green, blue, and red indicate wake, NREM, and REM, respectively.

10.1523/ENEURO.0094-22.2022.f1-3 Extended Data Figure 1-3 **Mean EEG slopes stratified by state and differences between slopes: CM-referenced dataset.** See the **Methods** for details on the calculation of spectral slopes. Download Figure 1-3, DOC file.

Evident in the raw power spectra, all cohorts other than the SHHS showed steeper 30- to 45-Hz slopes in NREM and REM than wake. Figure 1*b* shows estimated state-specific slopes for the six baseline cohorts. Briefly, given a power spectrum *PSD(f)* ∼ *1/f ^α^*, the spectral exponent β = –α was estimated as the linear slope of the log-log regression of power on frequency (see Materials and Methods). All three pairwise within-individual differences between wake, NREM, and REM slopes were highly significant (*p *<* *10^−15^ matched-pair *t* tests) and similar results were obtained for C3-M2 (Extended Data Figs. 1-2, 1-3).

10.1523/ENEURO.0094-22.2022.f2-1 Extended Data Figure 2-1 **Mean EMG slopes by state and cohort.** See **Methods** for details on the calculation of EMG spectral slopes. Download Figure 2-1, DOC file.

10.1523/ENEURO.0094-22.2022.f2-2 Extended Data Figure 2-2 **Associations between EEG and EMG spectral slopes in the CM-referenced dataset.** Standardized regression coefficients and associated p-values from a regression of EEG slope on EMG controlling for age (up to 5th-order polynomials), sex, cohort, race, BMI, AHI and AI. All analyses were performed in the CM-referenced dataset. Download Figure 2-2, DOC file.

10.1523/ENEURO.0094-22.2022.f2-3 Extended Data Figure 2-3 **EEG-EMG coherence and EEG spectral slopes**. a) For beta and gamma bands, median EEG-EMG coherence stratified by state and cohort. All analyses used CM-referencing. b) Correlations between EEG slope and EEG-EMG coherence, by state and cohort. Particularly during NREM, individuals with higher EEG-EMG crosstalk (i.e. coherence) tended to have steeper slopes. Green, blue and red indicate wake, NREM and REM respectively. Download Figure 2-3, TIF file.

**Figure 4.**
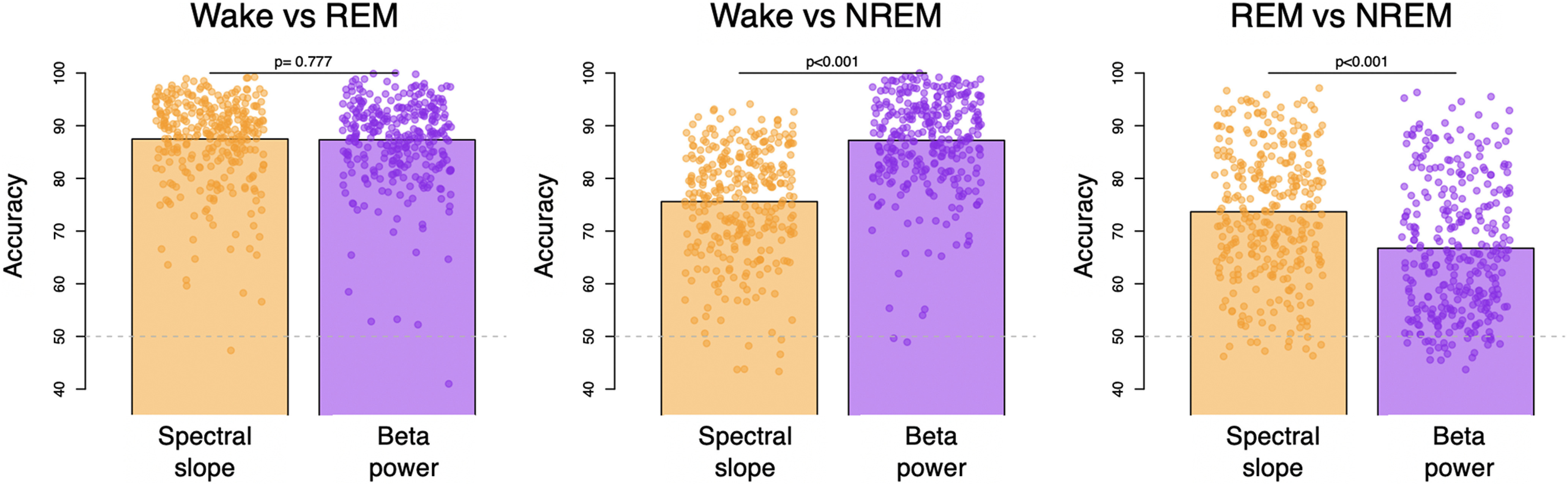
State classification using LDA based on spectral slope or absolute β band power. Three bar plots illustrate mean accuracies across individuals of W versus R, W versus N2, and R versus N2 classification and dots represent individual accuracies (orange, spectral slope as a predictor and purple β power as a predictor; see Extended Data Figure 4-1 with power of other classic frequency bands as a predictor); *P*-values above the bars indicate whether there was a significant difference between accuracies produced by LDA based on spectral slope versus β power. Dashed gray line illustrated chance level performance. For details, see Materials and Methods.

**Figure 5.**
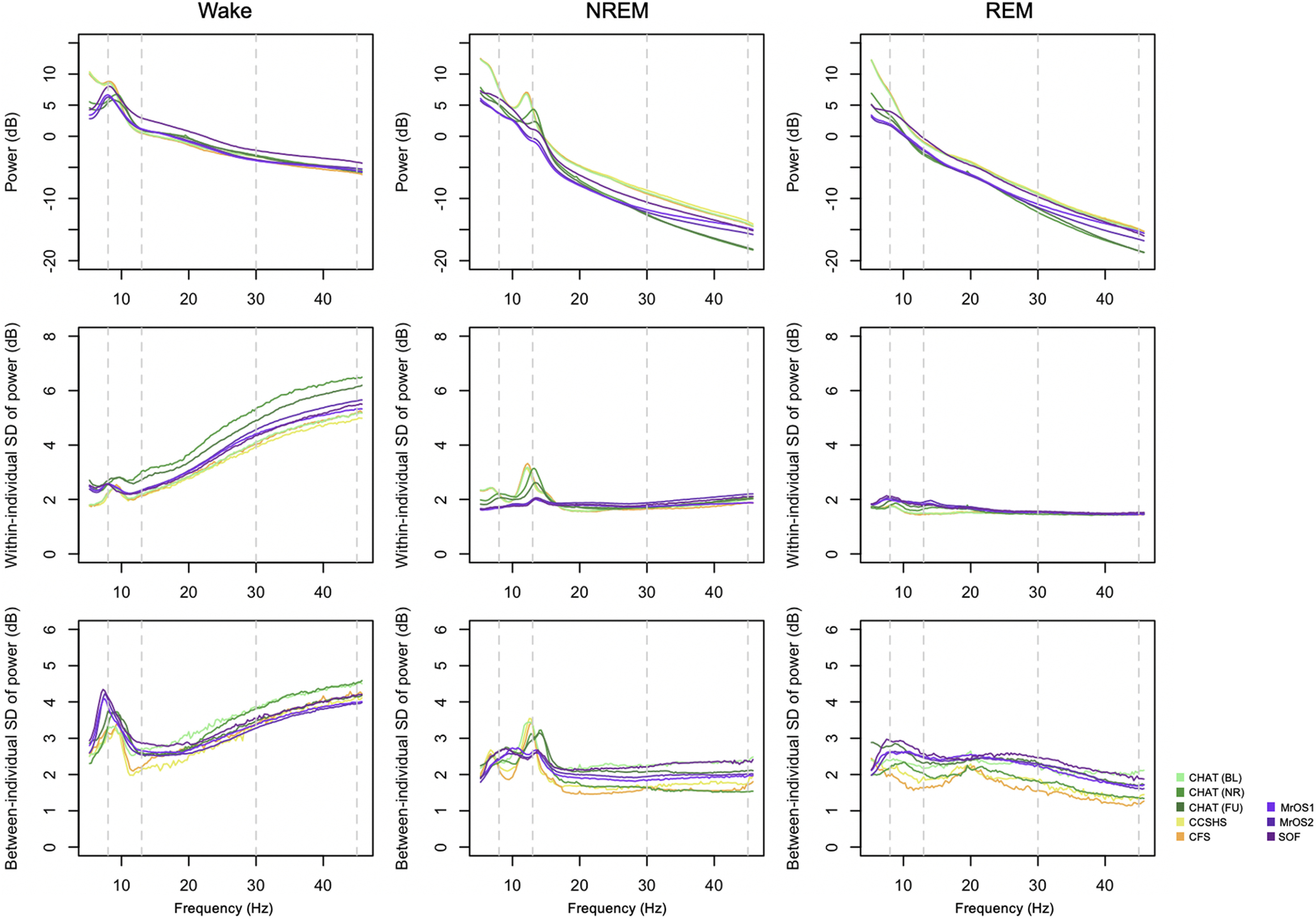
Means, within-person and between-person variability in state-specific spectral power. All analyses based on the LM-referenced dataset, with the SHHS studies excluded. Within-individual variability was based on the SD of epoch-to-epoch differences, calculated for each individual separately and then averaged over all individuals in each cohort. In contrast, between-individual variability was the SD based on differences between individuals’ mean power, calculated once for each cohort. See Extended Data Figure 5-1 for these data plotted individually for each cohort.

10.1523/ENEURO.0094-22.2022.f2-4 Extended Data Figure 2-4 **EEG spectral slope associations with sex and BMI in the CM-referenced dataset.** Coefficients and p-values from linear regression models of slope on sex and BMI, additionally controlling for age (and higher-order terms), race and cohort. Also see **Figure 2-5**. Download Figure 2-4, DOC file.

**Figure 6.**
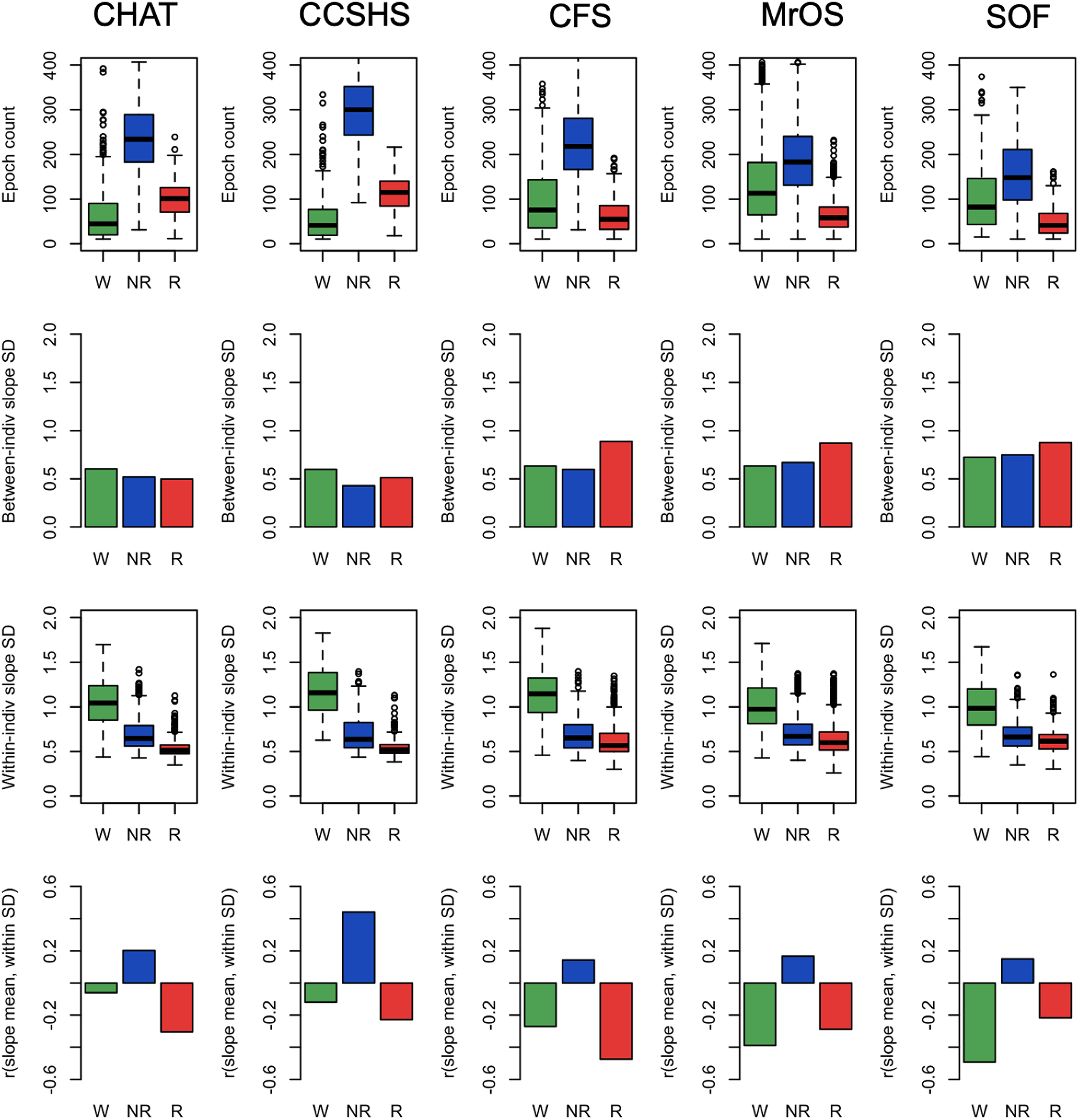
Epoch counts, variability in spectral power (within-individual and between-individual), and correlations between mean slope and within-individual slope SD. All analyses based on the LM referencing; all epoch counts refer to the number of epochs passing the stringent QC procedures. Green, blue, and red indicate wake, NREM, and REM, respectively. For details, see Materials and Methods.

10.1523/ENEURO.0094-22.2022.f2-5 Extended Data Figure 2-5 **Summary of sex && BMI associations with the EEG spectral slope.** Results indicate the signed -log10 p-value (as an index of relative effect size) from regressions of slope on sex, BMI and other covariates (see **Methods**). Only associations with *p*<0.01 are shown. Green, blue and red indicate wake, NREM and REM respectively. Also see tables Figure 2-4 & Figure 2-6. Download Figure 2-5, TIF file.

10.1523/ENEURO.0094-22.2022.f2-6 Extended Data Figure 2-6 **EEG spectral slope associations with sex and BMI in the LM-referenced dataset.** Coefficients and p-values from linear regression models of slope on sex and BMI, additionally controlling for age (and higher-order terms), race and cohort. The SHHS was excluded from all LM-reference analyses. Download Figure 2-6, DOC file.

**Figure 7.**
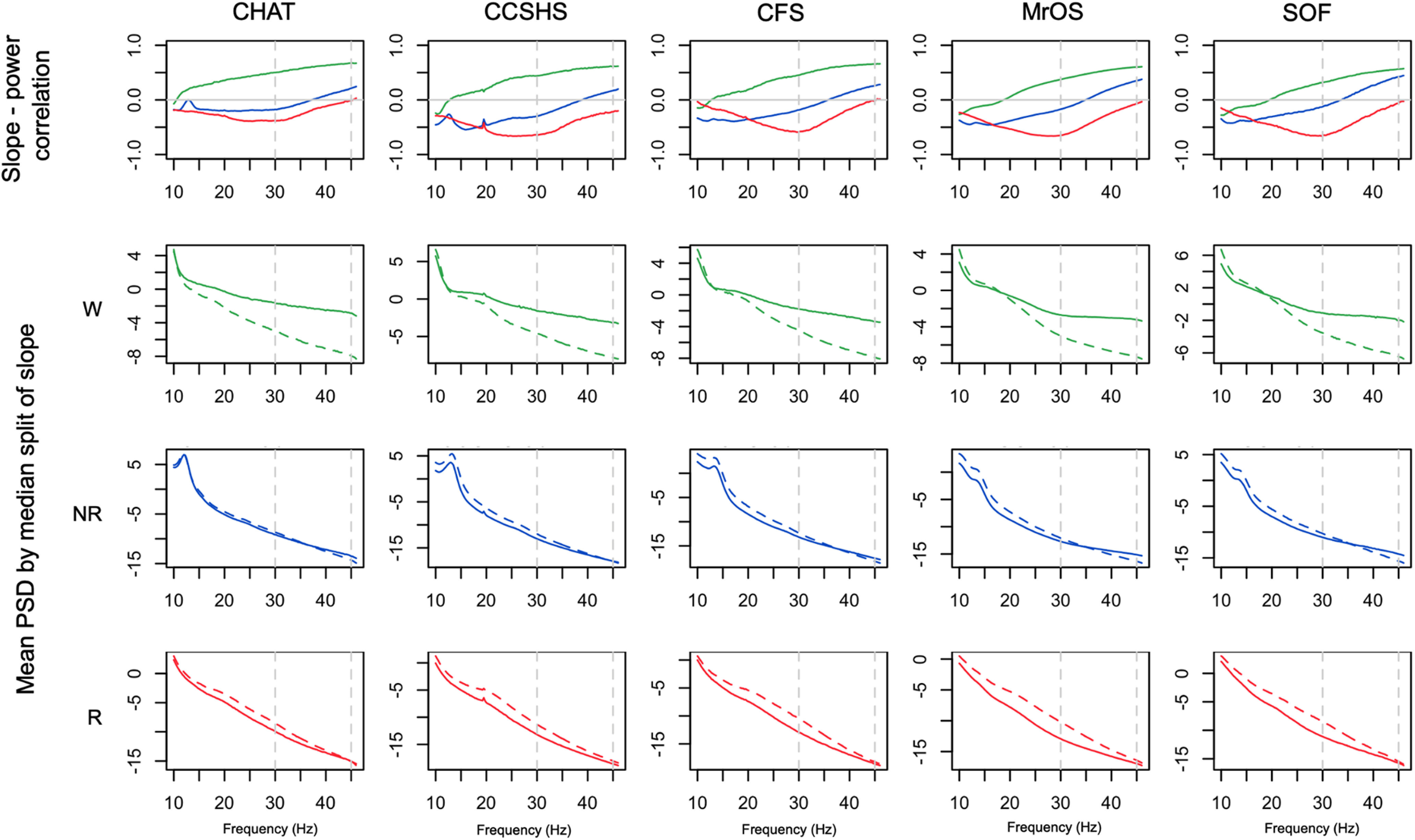
Relationships between spectral slopes and spectral power. Top row shows Pearson correlation coefficients between individuals’ mean spectral power and mean spectral slope, conditional on sleep state and cohort (for coherence, see Extended Data Fig. 7-1). Extended Data Figure 7-2 provides similar information for both central channels and extended frequency range. The lower three rows show mean power stratified by a median split on spectral power: means for the group of individuals with steeper slopes are represented by dashed (vs solid) lines. All analyses were based on the LM-referenced dataset. Green, blue, and red indicate wake, NREM, and REM, respectively.

10.1523/ENEURO.0094-22.2022.f2-7 Extended Data Figure 2-7 **Coherence between EEG and EMG/ECG in the CFS cohort.** Blue/red lines indicate mean coherence for males/females. Note that the use of linked mastoid referencing appeared to reduce EMG/ECG contamination, as indexed by spectral coherence with the EEG. Download Figure 2-7, TIF file.

**Figure 8.**
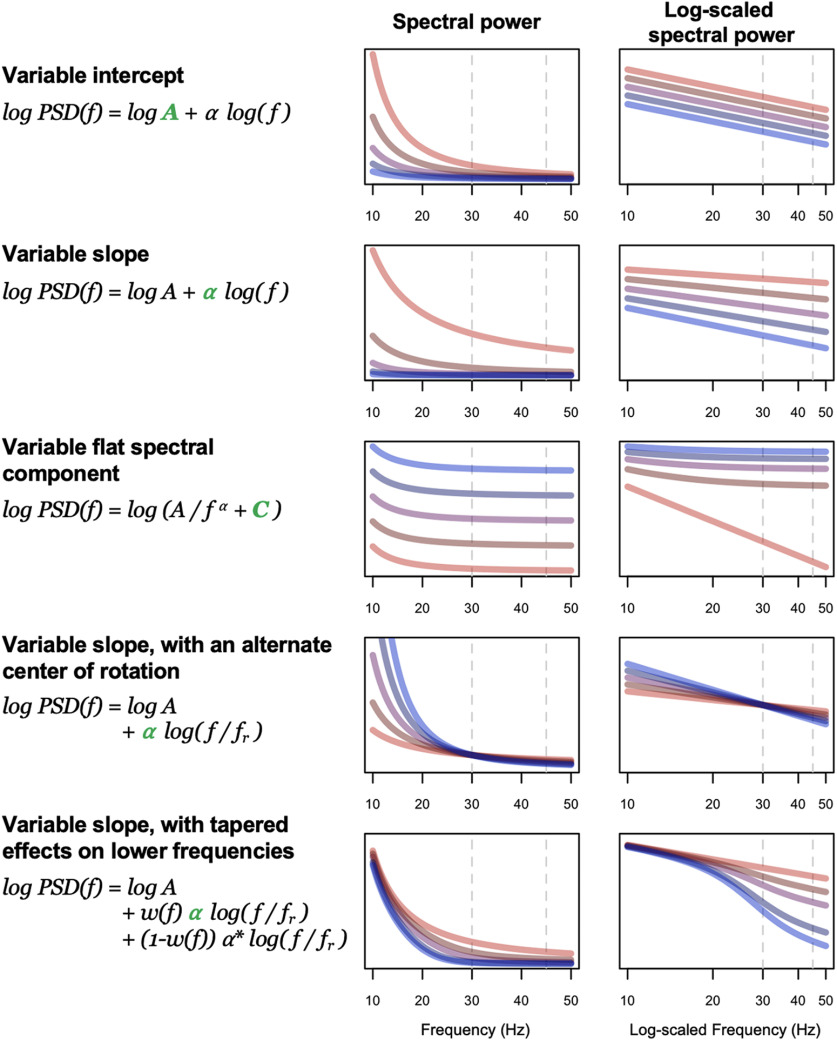
Model parameters. These cartoons illustrate the parameterizations of the aperiodic component of the power spectrum we considered in the simulations. In each case, the green term indicates the aspect of the model that was varied, and the corresponding plots show the impact of power spectra (in linear-linear and log-log coordinates, left and right figures, respectively). For illustrative purposes only, the five lines (from blue to red) show the expected power spectrum for five different parameter values (e.g., α = 1, 1.5, 2, 2.5, and 3). Power absolute values/units (*y*-axes) are arbitrary and so not shown: these figures are intended only to show some of the qualitative patterns of differences that can arise because of variation in a given model parameter. The *w(f)* function was similar to those depicted in Extended Data Figure 8-1*b*, with a 50% value at 25 Hz in this example (lower row). Beyond these factors, the model also allowed slope and intercept to be correlated and specified a stochastic error term (smoothed with respect to frequency). For details, see the text and Materials and Methods.

10.1523/ENEURO.0094-22.2022.f2-8 Extended Data Figure 2-8 **Associations between EEG and EMG slopes in the LM-referenced dataset.**Correlation coefficients and p-values for associations between EEG and EMG slopes in the LM-referenced dataset. Equivalent results are also reported for the CM-derived EEG slopes (in this same dataset). EEG-EMG correlations are attenuated comparing LM-derived to CM-derived estimates (albeit still significantly larger than zero). Similar results were obtained when controlling for age, sex, cohort and other covariates (see legend for **Figure 2-2** for details; note: **Figure 2-2** shows standardized regression coefficients from the adjusted model, and so the CM results are not directly comparable to the correlation coefficient presented here). Download Figure 2-8, DOC file.

**Figure 9.**
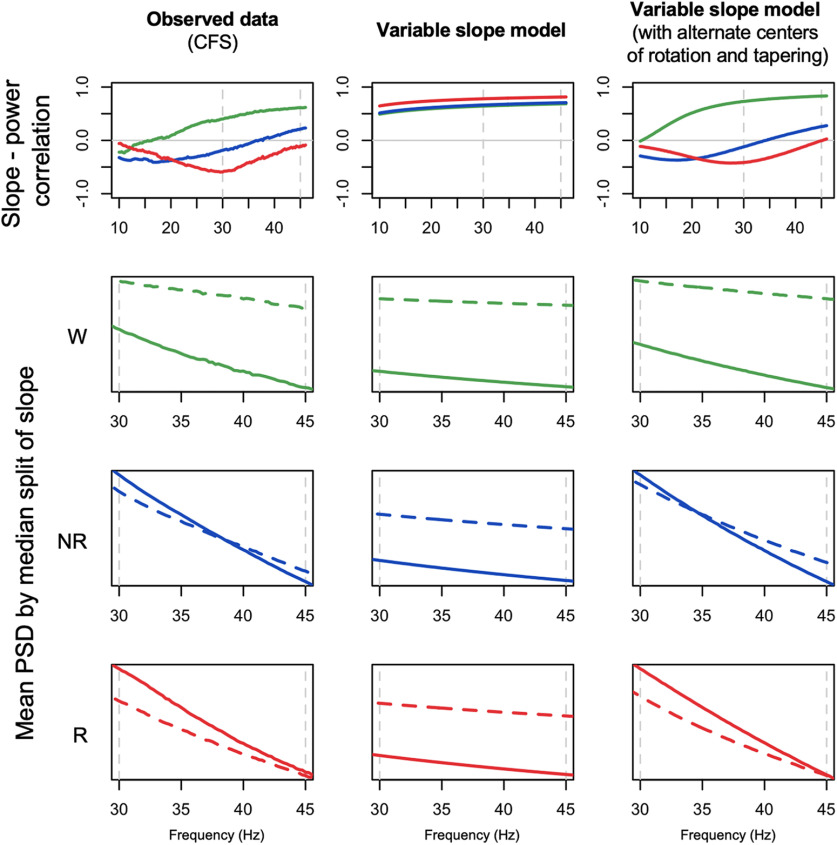
Observed data, initial and revised model simulation-based predictions. The left column of plots reproduces the observed results from the CFS cohort, for state-dependent slope-power correlations (top row) and mean power stratified by a median-split on slope (lower three rows). Green, blue, and red indicate wake, NREM, and REM, respectively. Based on *N *= 5000 simulated spectra, the middle and right columns show the equivalent simulation-based results, from the original model parameterization (“variable slope model”, the middle column), assuming a strict power law model with mean α = 1, 2.5, and 3 for wake, NREM, and REM, respectively (and SDs of 0.5, 0.5, and 0.75, approximately following the observed between-individual estimates from Fig. 6), and a revised model (“variable slope model with alternate centers of rotation and tapering”, the right column), with similar population parameters for slope means and variances but allowing different centers of rotation (*f_r_* = 10, 35, and 45 Hz for wake, NREM, and REM, respectively) and setting *w(f)* such that variation in *α* had less influence on the slope at lower frequencies (see Extended Data Fig. 8-1*b*). Whereas the initial model (simply varying mean spectral slope by state) could not recapitulate the observed results, the revised model could. For further details, see text and Materials and Methods.

10.1523/ENEURO.0094-22.2022.f2-9 Extended Data Figure 2-9 **Choice of reference and ECG artifacts.** The upper plot illustrates 10s of N2 from M1-Fpz, M2-Fpz, M1-M2 (the ‘cross-mastoid’) and (M1+M2)/2 (linked mastoid) for a subject from CFS with extreme cardiac interference in C**3**-M2. Fpz was the recording reference electrode. Both M1 and M2 time series are severely affected by ECG artifacts. Due to the opposite polarity, however, the linked mastoid (M1+M2)/2 signal almost fully eliminates this issue. The bottom plot shows the C**3** channel with different referencing (the recording reference Fpz, M2 and (M1+M2)/2) during the same period. Note how the contralateral mastoid reference introduced cardiac artifacts into the C**3** channel; however, if C**3** in fact contained strong cardiac artifacts prior re-referencing, using the contralateral mastoid would have helped to cancel it out. The vertical black bar on the left corresponds to 100 uV. Download Figure 2-9, TIF file.

### Technical factors in the SHHS datasets

As implied by the spectra in Figure 1*a*, the SHHS exhibited marked differences in mean power spectra and corresponding spectral slopes were markedly steeper in SHHS compared with other cohorts (Fig. 1*b* note the different *y*-axis scaling). Furthermore, most evidently during wake, the SHHS showed a bimodal distribution of slopes. presumably reflecting technical issues with the recordings. Indeed, SHHS wave 1 data collection occurred 1995–1998 (wave 2, 2001–2003) and relied on an early generation of amplifiers, Compumedics P-series Sleep Monitoring devices, that were more prone to artifact; for example, Compumedics noted potential issues with amplifier grounding. The P-series was not a truly digital system and, instead of being collected against a common reference, EEG inputs were hardwired into the amplifier interface and labeled EEG (C4-M1) and EEG2 (C3-M2), and so could not be re-referenced offline. Proprietary algorithms were applied to signals in a manner that was not readily transparent to SHHS study staff. Although the SHHS are described in the NSRR as having only a high-pass hardware filter set at 0.15 Hz, the power spectra in Figure 1*a* suggest these devices had a nonuniform frequency response over this range, leading to the supralinear spectral slope on the log-log scale across the 30- to 45-Hz range (with a “knee” around 30 Hz). The estimates of spectral slope were highly deviant for both SHHS waves: for example, for C4-M1, the mean wake β values were −8.1 and −7.6 for waves 1 and 2, respectively, in contrast to values close to −1.0 for all other cohorts (as expected for wake; Colombo et al., 2019).

We also observed a second type of difference within the SHHS: although still discrepant compared with other cohorts, estimated slopes for C3-M2 were not as steep as for C4-M1 (wake β = −5.5 and −5.5 for waves 1 and 2, respectively; Extended Data Fig. 1-1*a*). Furthermore, the bimodality in spectral slope noted for C4-M1 was not present for C3-M2 (Extended Data Fig. 1-1*a*). In SHHS1, ∼50 recording units were used, each for ∼100 participants; after refurbishment, the same physical devices were used in the smaller SHHS2. We found that device ID had a marked impact on the spectral slope for C4-M1 but not C3-M2, accounting for the bimodal spectral slope distribution (Extended Data Fig. 1-1*b*). Most individuals would not have had the same physical unit across both studies: we noted it was the same units (rather than the same individuals) who were outliers in each wave (Extended Data Fig. 1-1*b*).

These results suggest that (1) technical factors (presumably related to device frequency response characteristics, or subsequent low-pass filtering) impacted SHHS recordings in a manner that greatly biased the 30- to 45-Hz spectral slope, although (2) the effect differed between (hardwired) C4-M1 and C3-M2 channels, and (3) a further layer of device-to-device variability specifically impacted C4-M1 for ∼6–7 of the ∼50 devices used. As well as the 30- to 45-Hz spectral slope, these technical factors could potentially bias any analyses that (implicitly or explicitly) rely on the high (>30 Hz) frequency content of the SHHS EEG, e.g., machine learning on raw EEG time series for automatic staging. For these reasons, as well as the inability to re-reference to LMs, we excluded the SHHS from the majority of the analyses.

Nonetheless, although not a focus here, we note that the SHHS may still provide usable data for certain types of spectral slope analysis if one assumes that any technical differences were constant throughout the recording. Inasmuch as filtering reduced power by a frequency-dependent constant multiplicative factor, relative differences in log-scaled slopes may be expected to be preserved. For example, the REM – N2 relative slope distribution was unimodal and broadly similar across channels and SHHS waves, and also in comparison to other cohorts. Furthermore, motivated by the prior work on anesthesia, we hypothesized that benzodiazepine use (sedatives that lower brain activity) might be associated with steeper (more negative) slopes. Indeed, the 181 individuals in SHHS1 who reported regular benzodiazepine use had significantly steeper C3-M2 slopes, with similar effects across wake (*b* = −0.25, *p *=* *1 × 10^−5^), NREM (*b* = −0.30, *p *=* *2 × 10^−7^), and REM (*b* = −0.33, *p *=* *2 × 10^−4^), controlling for age, sex, BMI, race, Apnea-Hypopnea Index (AHI), and arousal index.

### Potential non-neural confounders of the spectral slope

The spectral slope in the γ range can be affected by muscle activity as the scalp EEG is inevitably sensitive to frontalis (peak frequency between 20 and 30 Hz) and temporalis (between 40 and 80 Hz; Goncharova et al., 2003; Muthukumaraswamy, 2013) as well as extraocular muscles (30–120 Hz with peak at 64 Hz) executing saccadic eye movements (Yuval-Greenberg et al., 2008; Carl et al., 2012). In a previous study comparing resting state EEG with and without paralysis induced by complete neuromuscular blockade, power above 20 Hz was attenuated 10- to 200-fold under paralysis, suggesting that most scalp EEG above 20 Hz is of EMG origin (Whitham et al., 2007). It also has been reported that during wake, resting state EEG recordings contaminated by muscle activity expressed flatter slopes than EEG with no EMG interference (Freeman et al., 2003). Neither are intracranial recordings necessarily completely free of muscle activity interference (Otsubo et al., 2008; Jerbi et al., 2009; Kovach et al., 2011; Ren et al., 2019). There are well-known differences in muscle tone and ocular movements between wake, NREM, and REM sleep; muscle atonia typical to REM sleep was reported to affect frontalis muscle to the same extent as submental muscle – a standard site of PSG EMG recording (Levendowski et al., 2018) but there is also evidence of increased facial muscle contractions because of limbic activation during REM (Rivera-García et al., 2011).

In the five cohorts excluding SHHS (CHAT, CCSHS, CFS, MrOS, and SOF), we therefore investigated possible sources of bias and/or noise, first estimating state-specific slopes from 30- to 45-Hz chin EMG spectra. Briefly, we observed multiple, potentially nontrivial linkages between state-dependent differences in EEG and EMG slopes: (1) similar wake > NREM > REM mean differences (Fig. 2; Extended Data Fig. 2-1) and age-related flattening (Fig. 2*b*); (2) positive associations in EEG and EMG slopes within state (Extended Data Fig. 2-2); (3) mean differences in EMG-EEG coherence (taken to index potential contamination) between states, primarily REM > NREM > wake (Extended Data Fig. 2-3*a*); (4) significant associations between EMG-EEG coherence and EEG slope (Extended Data Fig. 2-3*b*), and (5) modest but significant associations with BMI for both EEG and EMG NREM slopes (Extended Data Figs. 2-4, 2-5).

In the CFS, we additionally estimated 30- to 45-Hz slopes from the ECG, finding that wake slopes were less steep compared with NREM and REM slopes (matched pairs *t* test *p *<* *10^−15^, with means of −4, −5.1, and −5.2 for wake, NREM, and REM, respectively). ECG slopes were significantly correlated with EEG slopes, e.g., for C3-M1 *r *=* *0.15, 0.20, and 0.18 for wake, NREM, and REM (*p *=* *0.002, 4 × 10^−5^, and 2 × 10^−5^); these ECG-EEG slope associations persisted when additionally controlling for age, sex, race, BMI, AHI, and AI (data not shown).

In the CFS cohort, we estimated state-specific magnitude squared coherence between all EEG derivations and either the EMG or ECG (Extended Data Fig. 2-7). Particularly during sleep, there was high coherence between EMG/ECG and M1-M2, e.g., over 0.4 for EMG, and over 0.6 for ECG, with peaks in the σ/β band, but extending into γ frequencies. Males tended to show higher EEG-EMG/ECG coherence than females. Whereas the C3-C4 did not show strong coherence with EMG/ECG channels, both CM channels (C3-M2 and C4-M1) showed relatively high coherences (up to ∼0.4), presumably reflecting the extent to which EMG/ECG artifact was picked up at the mastoids.

Similar to Lendner and colleagues, conditioning on EMG slope was not sufficient to fully explain the observed, within-individual state-dependent differences in EEG slope – although we note that peaks in Lendner’s EMG power spectra at exactly 20 and 40 Hz suggest that their EMG slope estimates were themselves subject to bias/noise (see Lendner et al., 2020, their Fig. 2, supplement 2). Nonetheless, sources of between-individual, within-state variation (including noise and bias) may be quantitatively and qualitatively different from the sources of within-individual, between-state variation, meaning that non-neural confounds could still bias (either attenuating, or spuriously inducing) individual differences in the spectral slope.

Lendner and colleagues considered alternate referencing schemes including bipolar/local referencing: in our limited montage context, this corresponded to only the bipolar C3-C4 derivation. As a control, we also considered the “cross-mastoid” derivation M1-M2: although mastoid electrodes are not truly independent of neural activity, we expected any signatures of cortical arousal to be greatly attenuated, compared with the derivations involving a central scalp electrode. However, M1-M2 slopes often had greater effect sizes than C3-C4 slopes with respect to state differences (Extended Data Fig. 1-3) and, within state, were very highly correlated with EMG slopes (Extended Data Fig. 2-2) and BMI (Extended Data Figs. 2-4, 2-5). Given the potential role of mastoids introducing non-neural sources that might bias slope estimates, we alternatively considered a linked (i.e., averaged) mastoid (LM) referencing scheme. Although LM-derived slopes were correlated highly with contralateral (CM) slopes (e.g., for C3 *r *=* *0.93, 0.88, and 0.90 for wake, NREM, and REM, respectively) and exhibited comparable state-related differences (Extended Data Fig. 3-2), LM referencing (as originally employed by Lendner and colleagues) dramatically reduced the above mentioned markers of potential EMG/ECG-driven bias in EEG spectral slopes, e.g., as indexed by (1) EEG and EMG/ECG coherence (Extended Data Fig. 2-7); (2) correlation between EEG and EMG/ECG spectral slopes (Extended Data Fig. 2-8); and (3) likely spurious associations with BMI (Extended Data Figs. 2-5, 2-6). With regard to cardiac activity at mastoid channels because of their proximity to the carotid arteries, ECG potentials on the scalp have opposite polarities in the left and right hemispheres and are slightly asymmetric because of heart position, with higher amplitude on the left (Hamaneh et al., 2014). As illustrated in Extended Data Figure 2-9, LM referencing takes advantage of this to effectively cancel out much of the cardiac activity seen at each mastoid (conversely, the cross-mastoid M1-M2 derivation exacerbates it).

### LM analyses of the spectral slope by sleep state

Given concerns over (1) technical factors in the SHHS and (2) differences between LM-derived and CM-derived slopes, we based our primary analyses of the EEG spectral slope on LM referencing, with the SHHS dataset removed. We re-ran all outlier exclusions on this new dataset, yielding a final QC+ dataset of *N *=* *4459 recordings from *N *=* *3543 distinct individuals, of whom *N *=* *690 (primarily in MrOS) had a second PSG.

We observed unambiguous support for statistically different slope means, namely, wake > NREM > REM (Fig. 3; Extended Data Figs. 3-1*a* and 3-2). Averaging over cohort means, overall β = −1.11, −2.58 and −3.3 for wake, NREM, and REM, respectively [N1 and N3 exhibited similar slopes to N2 (−2.58), albeit typically marginally less steep, mean β = −2.4 and −2.34, respectively; Extended Data Fig. 3-2]. We also compared a goodness of fit in W, N2, and R and the average *R*^2^ estimates across all studies and stages were *R*^2^ = 0.7 or above. Although, *R*^2^ for wake was significantly lower than for sleep stages (*R*^2^ = 0.72 in wake, *R*^2^ = 0.98 in N2, and *R*^2^ = 0.98 in REM), in a sample of individuals with relatively high goodness of fit (*R*^2^ > 0.9 for all stages, 38% of all subjects), we still observed same pattern of slope significant differences between stages (wake > NREM > REM).

10.1523/ENEURO.0094-22.2022.f3-1 Extended Data Figure 3-1 **EEG spectral slope for C4-LM and using IRASA method. a.** See legend of **Figure 3** for details: this plot provides the same analysis but for C4-LM instead of C3-LM. Green, blue and red indicate wake, NREM and REM respectively. Also see Figure 3-2. b. EEG spectral slope in MrOS cohort for C3 referenced to linked mastoids. From left to right: slopes estimated: between 30 - 45 Hz using original method; between 30 - 45 Hz using IRASA method; between 1 - 30 Hz using IRASA method; between 5 - 30 Hz using IRASA method. Download Figure 3-1, TIF file.

10.1523/ENEURO.0094-22.2022.f3-2 Extended Data Figure 3-2 **Mean EEG slopes and state differences in the LM-referenced dataset.** Comparable state-specific slope means, and tests of state differences as shown in Figure 1-3 (for the CM-dataset/channels), but here for the LM-derived slopes. Download Figure 3-2, DOC file.

10.1523/ENEURO.0094-22.2022.f3-3 Extended Data Figure 3-3 **Cross-state correlations in EEG spectral slopes.** All statistics based on the LM-reference datasets (slopes for C3-LM and C4-LM). Correlations stratified by cohort, with outliers (+/- 3 SD units) removed. All correlations are significantly greater than 0 (p < 10-10). Download Figure 3-3, DOC file.

Despite mean differences, slopes were significantly correlated across states (Extended Data Fig. 3-3), suggesting systematic and state-independent factors influenced slope, other than arousal level per se. To a first approximation, N1, N2, and N3 slopes correlated *r* ∼ 0.7; for REM and NREM, *r* ∼ 0.5–0.7; for wake and sleep, *r* ∼ 0.2–0.5. As noted, given the broad similarity of N1, N2, and N3 slopes, all NREM analyses below used N2 sleep only, to ensure greater homogeneity in NREM sleep across individuals.

### Within-individual epoch-level discrimination of sleep state based on the spectral slope

Lendner and colleagues evaluated the extent to which the EEG spectral slope could be used to classify epochs as wake, REM, or NREM (N3). In comparison to slow oscillation (SO) power, the spectral slope enhanced discrimination of wake versus REM, and was comparable for wake versus NREM. Here, we adopted a similar LDA approach to classify epoch-level data in the CFS cohort (chosen because it contained the most diverse age range).

Given state definitions and scoring rules, it is not clear why one would expect SO power to be a particularly strong predictor of wake versus REM, however. We therefore focused on what we considered a more relevant comparison (for the question of discriminating REM from wake): different parameterizations of the higher frequency EEG, namely, β (15–30 Hz) and γ (30–45 Hz) band power, as well as other classic frequency bands.

Using LDA to discriminate wake and REM in the CFS cohort, the spectral slope did not perform differently compared with β power (*p *=* *0.78, with an average accuracy of 87.5% vs 87.3% for the slope; Fig. 4) and performed only marginally better than γ power (*p *=* *0.048, with 86.7%). In contrast, while the spectral slope performed better than β power for classifying REM versus NREM (*p *=* *10^−15^, 73.7% vs 66.8%), it was worse for classifying wake versus NREM (*p *<* *10^−43^, 75.6% vs 87.2%). Although higher frequency EEG activity may, as others have suggested (Liu et al., 2020), be an informative (and often overlooked) feature for distinguishing REM from wake, potentially driven by the EMG content of the high-frequency EEG, we did not find evidence that the spectral slope per se is an optimal parameterization for this particular goal. Indeed, here β power alone performed similarly (see Extended Data Fig. 4-1 for results with all classic frequency bands). Equivalent results were observed for relative power with respect to total 0.5- to 50-Hz power (data not shown).

10.1523/ENEURO.0094-22.2022.f4-1 Extended Data Figure 4-1 **State classification based on the spectral slope or absolute power across classic frequency bands.** Six bar plots on the left illustrate mean accuracies across individuals of W vs R, W vs N2, W vs N3, R vs N2, R vs N3 and N2 vs N3 classification and the error bars represent standard deviation in accuracies across individuals. Dashed grey lines illustrate the performance of spectral slope. The bar plot on the right represents the average goodness of fit (R2) of a linear model based on all stages together for a particular spectral metric ( spectral metric ∼ stage [W, N2, N3, R] + error). Download Figure 4-1, TIF file.

### Variability in spectral slope by sleep state

In addition to between-state differences in means, we also characterized state-dependent differences in the variability of spectral power and slope, considering both within-individual (epoch-to-epoch) and between-individual (person-to-person differences in means) sources of variation. Analogous to Figure 1*a*, but based on LM referencing and excluding SHHS, Figure 5 shows mean power spectra (top row) but also the variability (SD units) in power, partitioned into within-individual and between-individual components (middle and lower rows, respectively). Across all cohorts there was consistently greater variability in waking spectral power at higher frequencies, e.g., >20 Hz, increasing up to 45 Hz, consistent for both estimates of variability. There was also a tendency for greater variability at the points of canonical oscillatory activity for wake (i.e., ∼8 Hz) and NR (i.e., ∼13 Hz). In contrast, variability in REM power spectra was approximately uniform across this frequency range (Extended Data Fig. 5-1 shows these data plotted separately for each cohort).

10.1523/ENEURO.0094-22.2022.f5-1 Extended Data Figure 5-1 **Spectral power means and variability, by sleep state and cohort.** See legend of Figure 5 for details: here, figures are plotted separately by cohort rather than super-imposed (as in Figure 5). Green, blue and red indicate wake, NREM and REM respectively. Download Figure 5-1, TIF file.

We next considered the components of variability in the spectral slope (Fig. 6). Because the number of epochs observed for a given individual/state may influence the variance/error in estimated slopes (which will influence between-individual variability in mean slopes), we plotted the mean number of epochs for each state (Fig. 6, top row). As expected, individuals typically had more NREM epochs; there was also a tendency for older cohorts to have relatively more wake than REM epochs. In general, between-individual variability in slope (Fig. 6, second row) was similar across states, with perhaps the exception of greater variance for REM in the older cohorts (potentially reflecting their reduced REM duration). With respect to within-individual variability (Fig. 6, third row), there was a clear pattern of greater epoch-to-epoch variability in slopes during wake compared with REM, with intermediate levels observed for NREM, which may reflect greater heterogeneity and noise in the waking data.

We also considered the correlation between an individual’s mean slope and the corresponding epoch-to-epoch variability in slope (for that same individual). If, for a given state/individual, epoch-level estimates of slope followed a single normal distribution, one would not expect any significant correlation between mean and variance. Fig. 6, bottom row, shows the mean/variance correlations, averaged over individuals, separately by sleep state and cohort. Correlations were typically significantly different from 0.0 and followed a distinct pattern across cohorts: wake and REM slopes exhibited negative mean/variance correlations, whereas NREM slopes exhibited positive correlations. In other words, for REM and wake, individuals with more epoch-to-epoch variability in slope tended to have steeper (more negative) slopes, on average. The opposite was true for NREM: individuals with greater variability tended to have flatter slopes. These results suggest that looking only at a single value of the spectral slope (i.e., for one individual, an estimate based on all epochs) will miss other forms of state-dependent distributional differences in slope dynamics.

### Relationships between the 30- to 45-Hz spectral slope and power

As expected, the spectral slope was not independent of absolute (or relative) power across the spectrum. Consistently across all cohorts, we found that slope-power correlations (as well as slope-coherence correlations; see Extended Data Fig. 7-1) showed qualitatively different patterns between wake, NREM, and REM; however; Figure 7, top row, shows state-specific correlations between spectral slope (based on the 30- to 45-Hz interval) and power across a broader spectrum (10–46 Hz in 0.25-Hz bins; also see Extended Data Fig. 7-2). For example, considering power at 30 Hz we observed highly significant (*p *<* *10^−10^) negative correlations during REM, but positive correlations during wake. To visualize slope/power relationships more directly, Figure 7, lower three rows, also shows log-scaled absolute power stratified by a median split on spectral slope. Dashed (vs solid) lines represent mean power for individuals with steeper (vs shallower) slopes. During wake (Fig. 7, second row), steeper slopes resulted from greater divergence in power at higher frequencies (e.g., toward 45 Hz). In contrast, during REM (Fig. 7, bottom row) steeper slopes resulted from greater divergence at lower frequencies (10–30 Hz), which was attenuated at higher frequencies, i.e., up to 45 Hz. During NREM we observed an intermediate profile (Fig. 7, third row). The larger differences in wake higher frequency power echoed the increased variance in power and slope (Figs. 5, 6). That is, individual differences in waking power and slope were driven by factors that particularly influenced γ band power, whereas this was not the case during REM.

10.1523/ENEURO.0094-22.2022.f7-1 Extended Data Figure 7-1 **State-specific relationships between inter-hemispheric coherence, power and spectral slope.** Analyses based on CFS data only, all analyses based on the LM-referenced dataset. Green, blue and red indicate wake, NREM and REM respectively. Coherence was estimated using magnitude squared coherence, see Methods for details. a. Absolute coherence values were generally high (reflecting the common reference) but for beta and gamma frequencies we observed significantly lower coherence during REM compared to wake, with NREM showing an intermediate pattern. b. Coherence values were not independent of spectral power (here averaged across C3-LM and C4-LM), although we observed qualitatively different relationships between states. NREM exhibited a peak in power/coherence correlation in the sigma range (presumably driven by spindle activity), but also increased coherence/power correlation above 30 Hz. In contrast, during REM sleep there was an inflection point at 30 Hz, after which individual differences in coherence and power decoupled. c reproduces the slope/power correlation for CFS (as shown in Figure 7, but here based on the slope and power averaged over the two central channels). Finally, d shows the correlations between average slope (30-45 Hz) and coherence: as for slope and power, there were qualitatively different patterns between all three states. During REM, individuals with steeper slopes tended to show higher C3-C4 coherence, particularly around 30 Hz. In contrast, during NREM, individuals with steeper slopes tended to show lower coherence at higher (>20 Hz) frequencies, whereas for wake, individuals with steeper slopes tended to show lower coherence at slower (<20 Hz) frequencies. These results - alongside the prior results for the spectral power - underscore the types of qualitative state-dependent differences in measures related to the spectral slope, which appear to extend beyond simply differences in means. Download Figure 7-1, TIF file.

10.1523/ENEURO.0094-22.2022.f7-2 Extended Data Figure 7-2 **Correlations between EEG spectral slope and power.** All analyses based on the LM-referenced dataset. These Figures provide similar information as Figure 7 (top row) in the main text: here, all non-SHHS cohorts are plotted, results for both LM-referenced central channels are given; also, the x-axis extends < 10 Hz whereas Figure 7 excluded that portion of the power spectrum. Green, blue and red indicate wake, NREM and REM respectively. Download Figure 7-2, TIF file.

### Modelling state-dependent changes in the EEG power spectrum

We adopted a simplified, model-based approach to determine if, by themselves, changes in the spectral slope (assuming a strict power law model) were sufficient to account for the (qualitatively different) slope/power relationships depicted above, here focusing on spectral power/slope relationships (primarily Fig. 7). We directly simulated power spectra for *N *=* *5000 individuals, initially in the form PSD(*f*) = *A*/*f^α^* + *e*, where *A* and *α* were normally distributed random variables representing the spectral intercept and exponent (slope β = –*α*), respectively (see Materials and Methods). Loosely following others (Miller et al., 2009; Podvalny et al., 2015; Gao, 2016), we then extended the model in several ways; schematically depicted in Figure 8, these (nonmutually exclusive) parameterizations were as follows: (1) allowing spectral slope and intercept to be correlated (positively or negatively); (2) including a flat spectral component *C*, such that PSD(*f*) = *A*/*f^α^* + *C*; (3) allowing an alternate center of rotation *f_r_* such that log PSD(*f*) = log *A* + *α* log (*f*/*f_r_*); and/or 4) allowing for the slope to vary across the power spectrum. The latter was implemented by modeling two independent slopes (*α* and α*) along with a frequency-dependent sigmoidal weight function *w(f)* (Extended Data Fig. 8-1, first column), such that the spectrum was in the form log PSD(*f*) = log *A *+* w(f) α* log (*f*/*f_r_*) + (1-*w(f)*) α* log (*f*/*f_r_*) . Although the second slope α* could in principle be parameterized differently from *α* (i.e., in terms μ, σ, *f_r_* and correlation with the intercept, to model a “knee” in the mean spectral slope), here these were fixed to the same values for *α*. The relevant point is simply that, under these “tapered” models, changes in *α* will tend to influence the slope only at higher frequencies (e.g., >30 Hz) but not at lower frequencies (e.g., <20 Hz), as modelled by *w(f)*.

10.1523/ENEURO.0094-22.2022.f8-1 Extended Data Figure 8-1 **Simulated power spectra with estimated power, spectral slopes, and their correlations.** Based on N = 5,000 simulated spectra, derived statistics (columns 2 to 6) for a) the original model parameterization, assuming a strict power law model with mean α = 1, 2.5 and 3 for wake, NREM and REM respectively (and SDs of 0.5, 0.5 and 0.75, approximately following the observed between-individual estimates from Figure 6), and b) a revised model, with similar population parameters for slope means and variances but allowing different centers of rotation (*f*_r_ = 10, 35 and 45 Hz for wake, NREM and REM respectively) and setting *w(f)* such that variation in α had less influence on the slope at lower frequencies. Green, blue and red indicate wake, NREM and REM respectively. See Methods for details. Download Figure 8-1, TIF file.

We initially set population slope parameters *α* ∼ *N*(μ, σ^2^) to μ = 1, 2.5, and 3 to reflect typical observed means for wake, NREM, and REM, respectively. In the simulated power spectra, we followed the analysis procedures as above, estimating state-dependent variability in power, the mean slope, the correlation between slope and power, and mean power stratified by a median split on slope (Fig. 9; Extended Data Fig. 8-1*a*). Given the consistency across studies evident in Figure 7, here we reproduce only the CFS results in Figure 9, first column, to reflect the empirical, observed values for key statistics, focusing on the state-dependent slope-power correlation and slope-stratified mean power.

Our initial model, which assumed a strict power law implying a rotation of the slope around 1 Hz as a function of *α* (Fig. 9, second column; Extended Data Fig. 8-1*a*), was unable to recapitulate the qualitatively different patterns of slope-power correlation we observed across states. Namely, slope and power were always positively correlated, meaning that individuals with steeper slopes tended to have lower power at all frequencies above 1 Hz. In a revised model, we observed two alterations that were sufficient to account for the major, state-specific pattern of results (Fig. 9, third column): changing the center of rotation (*f_r_*) and allowing for a frequency-dependent tapering of the influence the *α* parameter on the spectral slope, via a nonuniform *w(f)*. Specifically, we set *f_r_* to 10, 35, and 45 Hz for wake, NREM, and REM, respectively. Changing the center of rotation allowed for the qualitatively different slope-power relationships observed across states. However, by itself, changing only *f_r_* yielded slope-power correlations that grew very negative at lower frequencies for NREM and REM. To achieve the attenuated correlations we observed (i.e., trending back toward *r *=* *0 by ∼10 Hz), it was sufficient to assume *w(f)* functions as shown in Extended Data Figure 8-1*b*, first column, implying that influences on the slope at these lower frequencies were independent of factors impacting the 30- to 45-Hz slope.

Fully exploring these parameterizations is beyond the scope of this report and other modifications of the basic model may yield predictions that are equally consistent with our observations. These analyses were intended only to provide qualitative insights rather than quantitative fits to the data; these models also did not consider within-individual patterns of variability as documented above, parameterizing only the mean slope and spectra for each individual. Further, these models only considered the aperiodic component of the power spectrum. In real data, oscillatory activity during wake and NREM will impact observed slope-power correlations at lower frequencies (e.g., in Extended Data Fig. 7-2, the childhood cohorts show dips in absolute slope-power correlations near α and σ frequencies for wake and NREM, respectively, presumably reflecting individual differences in α/spindle rhythms not strongly related to the spectral slope). In our supplementary analysis, we aimed to eliminate any potential impact of oscillatory activity using IRASA method (Extended Data Fig. 3-1*b*) and observed similar stage-specific patterns of spectral slope as in our original analysis. These results suggest that it was not simply reflecting unaccounted-for periodic components. Slopes estimated based on <30-Hz frequencies did not yield the same NR > R pattern, however. Assuming that these differences are not driven by oscillatory activity (i.e., as per the premise of IRASA), these results are indeed consistent with our simulations, which pointed to the likelihood of variable slopes across the frequency spectrum and analyses that estimate a single slope across a large frequency range may therefore be difficult to interpret.

### Demographic sources of variation in state-specific spectral slope

**Figure 10.**
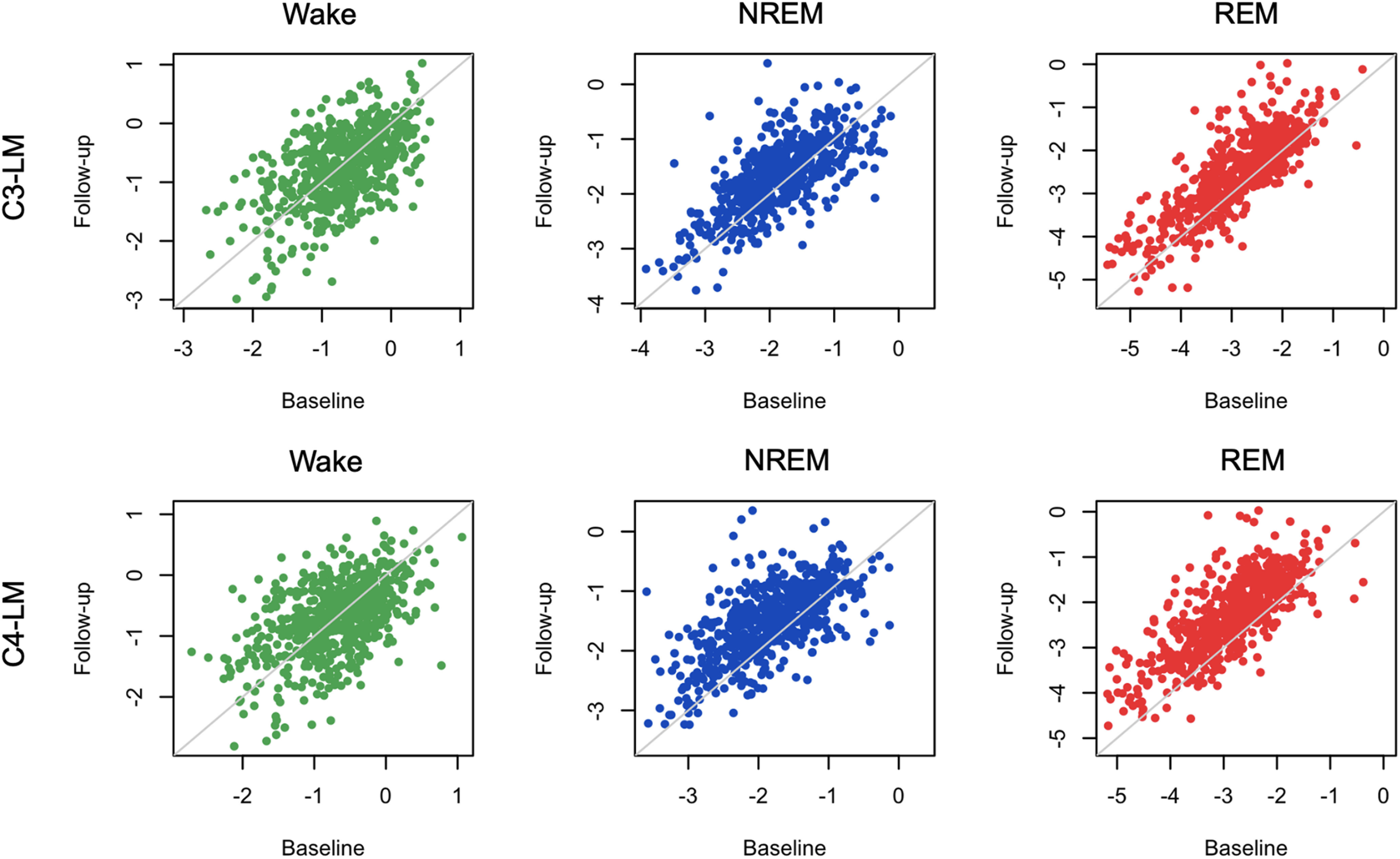
Repeated spectral slope assessment in the MrOS cohorts (waves 1 and 2). All analyses were based on the LM-referenced dataset. *N *=* *610 individuals had QC+ recordings for all states in both MrOS1 and MrOS2. Visits were typically approximately five years apart (mean ages of 76.4 and 81.1 for waves 1 and 2, respectively). Green, blue, and red indicate wake, NREM, and REM, respectively. See Extended Data Figures 10-1 and 10-2 for same in CHAT dataset. Age-related effects in a cross-sectional analysis are presented in Extended Data Figure 10-3.

Finally, we considered how the spectral slope varied with age and sex, and whether those effects varied between wake, NREM, and REM. First, we leveraged the repeated PSGs from the MrOS cohort of older men (approximately five years between visits) to estimate test-retest reliability as well as age-related changes in a longitudinal/within-individual context. Spectral slopes showed moderate to high test-retest correlations (Fig. 10; Extended Data Fig. 10-1). For example, for C4-LM, the test-retest *r *=* *0.51, 0.63, and 0.75 for wake, NREM, and REM, respectively (all *p *<* *10^−15^; Extended Data Fig. 10-1). We further observed significant flattening of slopes over time, albeit only for NREM and REM, with differences of 0.00, 0.32, and 0.57 for wake, NREM, and REM, respectively (matched pairs *t* test *p *=* *0.98 for wake, and *p *<* *10^−15^ for NREM and REM). Similar patterns held in MrOS for C3-LM (Extended Data Fig. 10-1).

10.1523/ENEURO.0094-22.2022.f10-1 Extended Data Figure 10-1 **Test-retest correlations and mean differences in the EEG spectral slope (MrOS and CHAT).** Also see **Figure 10** (MrOS) and **Figure 10-2** (CHAT) for plots of test-retest EEG slope distributions. Download Figure 10-1, DOC file.

10.1523/ENEURO.0094-22.2022.f10-2 Extended Data Figure 10-2 **Longitudinal analyses of the CHAT cohort.** Based on the LM-referenced dataset, scatter-plots show the EEG spectral slope for baseline and follow-up CHAT (childhood) studies (N = 80 pairs post QC, ∼6 months interval) stratified by sleep state and channel (C_3_-LM and C_4_-LM). Green, blue and red indicate wake, NREM and REM respectively. See Figure 10-1 for statistical results. Download Figure 10-2, TIF file.

10.1523/ENEURO.0094-22.2022.f10-3 Extended Data Figure 10-3 **Cross-sectional analysis of age-related flattening of the spectral slope.** All statistics are based on the LM-reference datasets, using a multiple linear regression model of EEG slope (here average of C3-LM and C4-LM) on age (linear) plus covariates, performed within cohort. CCSHS was excluded as there was effectively no variation in age (most participants were either 17 or 18 years of age). Similar patterns of results were obtained for analyses of each individual LM-referenced channel. Download Figure 10-3, DOC file.

Furthermore, all pairwise between-state differences in slope (i.e., R-W, R-NR, and NR-W) had similarly high test-retest correlations (e.g., *r *=* *0.67 for R-NR; Extended Data Fig. 10-1). In absolute terms, the magnitude of state differences grew smaller over time (all *p *<* *10^−15^), meaning that states with initially steeper slopes showed greater age-related decline (i.e., REM > NREM > wake). As noted, MrOS is an elderly cohort (mean ages were 76.4 and 81.1 years in waves 1 and 2, respectively). Potentially because of either increased noise in estimated slopes during wake, or a form of floor/ceiling effect in age-related change, we did not observe significant flattening of the wake slope in this cohort. In contrast, however, REM slopes showed the greatest age-related flattening, potentially suggesting that the sleep-based spectral slope is a more sensitive measure of aging in this population.

Extended Data Figure 10-2 (and Extended Data Fig. 10-1) shows the comparable results for the longitudinal component of the CHAT cohort. Although, because of the extended WASO inclusion criterion, the portion of CHAT passing QC in both waves was relatively small (*N *=* *80 pairs), we still observed significant test-retest correlations, but only during sleep (for C4-LM, *r *=* *0.08, 0.71, and 0.79 for wake, NREM, and REM, respectively, with *p *=* *0.46 for wake and *p *<* *10^−10^ for both sleep states). CHAT PSGs were only separated by approximately six months on average, and so mean differences may not necessarily be interpretable. With that said, a six-month interval during childhood represents a greater developmental window, and previously we did find significant within-individual differences in spindle activity in CHAT consistent with broader cross-sectional trends seen in larger datasets (Purcell et al., 2017). In the present longitudinal CHAT analysis, we observed nominally significant (*p *<* *0.01) age-related change, but only for a modest flattening of REM slopes (Extended Data Fig. 10-1).

We also evaluated age-related changes in the spectral slope cross-sectionally, separately for each cohort (taking only the first recording for individuals with a repeated PSG). Results broadly pointed to age-related flattening of slopes, and particularly for REM, although there was some degree of ambiguity and potentially inconsistency across studies. Both CHAT cohorts (prepubertal children, ∼6–10 years) cross-sectionally showed strong age-related flattening for NREM and REM slopes, with a weaker flattening of the wake slopes (Extended Data Fig. 10-3). Cross-sectionally, MrOS showed a flattening only of the REM slope (Extended Data Fig. 10-3); this stronger REM effect was consistent with the longitudinal MrOS analysis above (Extended Data Fig. 10-1). In contrast, the smaller SOF cohort of women of very advanced age (75–95 years) did not show any age-related changes (Extended Data Fig. 10-3), whereas the CFS cohort (which has the broadest age range, from 7 to 89 years) showed an inconsistent pattern, with a flattening of NREM slope (*b *=* *0.005 slope units per year, *p *=* *0.006), but a steepening of REM slope (*b* = −0.01, *p *=* *10^−4^) and no change in wake slope (*p *= 0.68). Secondary analyses pointed to possible nonlinear age-related change for REM slopes in CFS (e.g., *p *=* *5 × 10^−6^ for a second order, orthogonal polynomial age term, in a regression of REM slope controlling for sex, BMI, race and AHI/AI). Fuller exploration of possible nonlinear age-related trends (incorporating potential cohort-specific effects also, given that these NSRR cohorts do not overlap greatly in age ranges) is beyond the scope of the current manuscript.

Finally, we observed consistent sex differences in spectral slopes, whereby males tended to have flatter slopes than females (Extended Data Figs. 2-4, 2-5). In the primary analyses, we pooled MrOS (all males) and SOF (all females) as a single cohort to facilitate the analyses of sex differences while controlling for cohort effects as best as possible. In the combined analysis (CHAT, CCSHS, CFS, and MrOS+SOF, controlling for age, race, cohort and BMI) male slopes differed (i.e., were less steep) by 0.18, 0.34 and 0.32 units for wake, NREM, and REM (*p *=* *3 × 10^−8^ for wake, and *p *<* *10^−15^ for NREM and REM; Extended Data Fig. 2-4). As illustrated in Extended Data Figure 2-5, sex differences were statistically stronger for the final LM-referenced analysis compared with the original CM-referenced analysis), although sex differences were observed consistently across all channel derivations, all cohorts and all states (Extended Data Figs. 2-4, 2-5).

## Discussion

Using a diverse collection of cohorts from the NSRR, we replicated Lendner and colleagues’ report of progressively steeper spectral slopes (going from wake, to NREM, to REM sleep), based on the 30- to 45-Hz EEG. We further pointed to several technical issues that had the potential to impact estimation of the spectral slope, with a focus on potential EMG and ECG contamination, and the choice of mastoid referencing scheme. Based on thousands of diverse studies across multiple settings and with different equipment, our core results, the qualitative pattern of within-individual between-state differences in average slope, appeared to be robust to these factors.

The 30- to 45-Hz spectral slope is an appealing metric: as well as being easy to compute, it explicitly focuses on two components of the sleep EEG that, historically, have often been ignored: the aperiodic component, and “high”-frequency activity (in our context of a typical PSG, here meaning >30 Hz). With regard to the latter point, following AASM guidelines for staging, many analyses of the sleep EEG begin by bandpass filtering in the 0.3- to 35-Hz frequency range. This is often warranted: the most easily recognizable (and oscillatory) features of the sleep EEG are <20 Hz, and it is well documented that the lower amplitude, higher frequency EEG is more susceptible to artifact (Hipp and Siegel, 2013; Muthukumaraswamy, 2013). Nonetheless, as others have noted, even if, or precisely because, the high-frequency EEG contains muscle information, it may still be informative for staging, and particularly for discriminating wake versus REM. While our study does not directly address the question as to whether including high-frequency EEG improved automated staging in our cohorts, we did not find any evidence to suggest that the spectral slope per se was an optimal parameterization for state discrimination, compared with other simple summaries such as band power. That is, the spectral slope may remain a conceptually distinct and powerful biomarker to be used in other contexts, including within-state, between-individual analysis, but its comparative utility for sleep staging has not, in our opinion, been proven yet.

Although Lendner and colleagues demonstrated that slopes could be reliably estimated with different referencing schemes, our study suggested that CM referencing was more likely to be prone to bias and/or noise from non-neural sources. Adopting a LM referencing scheme appeared to largely (but not completely) mitigate these biases, for example, as indexed by spectral coherence between EEG and EMG/ECG, or correlation between spectral slopes derived from these different modalities. Dependencies between EEG, EMG, and ECG could arise because of physiologically driven artifact: for example, the potential for muscle artifact being picked up in the high-frequency (here >30 Hz) EEG. Alternatively, sources of noise shared across sensors (e.g., electrical noise, movement) could lead to coordinated changes in (mis-)estimated slopes. Beyond these factors, however, it remains a possibility that nonspurious, physiological linkages lead to partially coordinated slopes. There are well characterized differences between states in neural, cardiac and muscle activity, and so state-dependent changes in cortical arousal could drive and/or co-occur with other central and peripheral nervous system changes.

Importantly, the sources of variation (either physiological or artefactual) that drive individual differences in spectral slopes may differ (both quantitatively and qualitatively) from those that generate within-individual differences. That is, even if changes in slope are reliable indicators of changes in arousal levels within individuals, estimated slopes are not necessarily unbiased biomarkers of individual differences in the same phenomena. For example, if body mass index had a systematic bias on the slope (e.g., via differential cardiac/muscle contamination of the EEG), this could lead to spurious interpretations of linkages between cortical arousal and BMI, even if slope were an unbiased marker of cortical arousal level within-individual. In this spirit, we therefore sought a better understanding of the sources of variability in this metric, to realize its potential as a biomarker in research or clinical contexts, especially those involving comparisons between groups, such as neuropsychiatric patient populations.

We also discovered several issues with the EEG data in the SHHS datasets, including channel and device-specific differences. Other studies that use SHHS data, collected using very early portable EEG monitoring devices, should be aware of these effects (which we will document via the NSRR website, including the device IDs associated with differences in the spectral slopes). While these issues very directly impact analyses based on the spectral slope, other forms of analysis that implicitly use high-frequency information (e.g., applying machine learning to the raw time series) could presumably be susceptible to noise and/or bias for these same reasons.

As well as replicating the differences in the means, we reported differences in other aspects of the state-specific slope distributions, including its variability (both between and within individuals) and covariation with spectral power and coherence. It is noteworthy that, despite the common characterization of REM as “paradoxical” sleep (i.e., brain “active/wake-like” but muscles “inactive/asleep”), we found that REM and wake often showed the most divergent EEG metrics, with NREM sleep being an intermediate. Specifically, as well as the mean spectral slope, we observed this pattern for (1) within-individual slope variability, (2) frequency-dependent covariation between spectral power and slope, and (3) interhemispheric γ coherence. With respect to the relationships between slope and power, the observed state-dependent effects could not be accounted for by only a change in the spectral slope, under a strict power law model. Using a simplified model to provide qualitative insight (Miller et al., 2009; BJ He et al., 2010; Gao et al., 2020), the state-specific power-slope correlations we observed were consistent with (1) changes in the center of rotation of the slope and (2) a restricted influence at lower frequencies of the factors that determined 30- to 45-Hz slopes (even accounting for oscillatory activity, that below 30 Hz activity shows sources of variation in the spectral slope that are distinct from > 30 Hz, and particularly during R). Based on a cursory initial evaluation, altering the mean and/or variance of the spectral intercept, its correlation with the spectral slope, and/or the presence of a flat spectral component (Gao, 2016) were not sufficient to account for our observations, although more work to quantitatively model our data (including any individual differences, e.g., with respect to age and sex) may be warranted.

Finally, we considered individual differences in state-specific spectral slopes, in particular test-retest stability and age-related change. In longitudinal analyses based on a subset of individuals with a repeated PSG (typically around five years later), we observed moderate to high stability of spectral slopes, particularly during sleep. This is consistent with a prior report on a smaller sample where intrasubject reliability of spectral slope (2–25 Hz) was measured on two separate resting state recordings performed on the same day (Pathania et al., 2021). We also observed statistically significant age-related reduction (flattening) of slopes during sleep. In cross-sectional analyses, we observed broadly consistent age-related effects in most but not all cohorts, perhaps suggesting unaccounted for between-cohort sources of variability, or nonlinear age-related effects in the CFS, the cohort with the widest age range. Flatter slopes (2–24 Hz) in older adults in cross-sectional samples were previously reported based on wake recordings during task performance (Voytek et al., 2015; Dave et al., 2018), and similar effects of age-related flattening were observed in children (approximately eight years old) whose slopes were steeper during rest than in adults (W He et al., 2019). Our results, however, suggested that slopes during REM appear to be particularly sensitive to age-related change. Further, the differences in slope between NREM and REM (or wake and REM) showed significant test-retest and age-related changes. Future work might investigate potential linkages between REM spectral slopes and biological aging, including the emergence of synucleinopathies including Parkinson’s disease, that are often preceded by changes in REM sleep.

Although large, this study is not without limitations. Perhaps most obviously, analyses were restricted to a very limited montage: two central channels (although Lendner and colleagues noted that state-related changes in the 1/*f* slope were broadly reflected across the scalp). A second caveat is that, although Lendner and colleagues also included wake epochs after sleep onset in their final analyses, our studies, many of which were conducted in participants’ own homes, did not systematically include any “quiet rest” periods before sleep, and wake periods were often noisy, especially at the starts and ends of recordings (potentially reflected in higher variability, and lower goodness-of-fit measures in slope during wake). We therefore imposed a relatively strict epoch-wise filtering scheme; as such, although our results generally show a high degree of consistency, findings that point to attenuated effects during wake (namely, test-retest reliability and age-related flattening) should be interpreted with this caveat in mind. Finally, the few instances of inconsistency between studies (i.e., age-related trends in the CFS cohort, which had a very broad age range and was enriched for individuals with sleep apnea) might point to factors that require different approaches (e.g., use of nonlinear modeling or more stringent QC).

Overall, as seen in other areas of electrophysiological research, theoretically-inspired alternative parameterizations of the sleep EEG have much promise, although better characterizing the sources of variation in these measures, whether from artifact, from state-related changes in arousal, or from demographically and medically relevant differences in physiology, remains an important challenge.

## Synthesis

Reviewing Editor: Christina Zelano, Northwestern University - Chicago Decisions are customarily a result of the Reviewing Editor and the peer reviewers coming together and discussing their recommendations until a consensus is reached. When revisions are invited, a fact-based synthesis statement explaining their decision and outlining what is needed to prepare a revision will be listed below. The following reviewer(s) agreed to reveal their identity: Thomas Donoghue, Janna Lendner.

The reviewers and I agree that this manuscript is useful, and timely, and that the study was well-executed. Both reviewers had some relatively minor suggestions which will improve the manuscript, and should be addressed, see below:

Reviewer 1:

In this paper, the authors do a deep dive investigating best practices for measuring the spectral slope in sleep EEG data, and evaluate the findings from pursuing this analysis across multiple large datasets. I find this investigation to be useful, important, extremely thoroughly executed, and to reflect a very well done project. Overall, I wish to commend the authors, both for doing this kind of project - digging deep into potential methodological issues and doing due diligence to validated their findings across such large datasets - and for executing it so well. I find nothing in this paper with which I take issue, and see, to my eyes, no issues with any of the analyses or main interpretations in this paper.

I have two minor comments that could potentially be noted in this paper:

One comment I had as I read the article was whether the authors examined any goodness-of-fit measures of the spectral slope estimates in their analyses. This is often examined, for example, in terms of the r-squared fit between the fit line and the underlying data. This is used, for example, in the Lendner paper, including in some of the analyses they do comparing different methods and pre-processing, which are conceptually similar to some of the analyses in this paper. I don’t see any goodness-of-fit measures in this manuscript. I wonder if this is something that the authors explored at all? I understand that this paper employs stringent quality control procedures, and it is not that I generally doubt that the quality of the fits here. In general, however, I do think this kind of control check can be useful for these kinds of analyses, that could perhaps be noted if it is something the authors explored, and/or perhaps simply mentioned in the Discussion section, if this is something the authors which to note as a potential recommendation for other work applying these measures.

The second comment I had is regarding the chosen frequency range and how to fit it. This paper analyzes the 30-45 Hz range, and does so with a robust linear fit, following the Lendner paper. However, in this paper the simulations also note that a “variable slope model", reflecting power spectra with multiple exponents and a knee, seems to explain the overall changes in the spectrum. Given this, do the authors think that future work should perhaps investigate using different methods and fitting ranges to, for example, fit a broader range with a model that includes the knee? This is now available in other fitting approaches, such as the spectral parameterization method (Donoghue et al, 2020), which can parameterize knees as well as extend to broader frequency ranges while controlling for any oscillatory peaks. Alternatively, are there reasons to prefer the 30-45 Hz range as is the focus here? For this comment, I don’t expect or think that the paper needs additional analyses applying different ranges and models, but it was a question left in my mind that could perhaps be addressed in the discussion section, in particular in terms of thinking about the implications of the simulation results, and whether the authors recommend that other fitting methods and models could and should be applied in future work, and/or if the recommendation would be to stick with the exact measures used here.

Minor description notes:

- In the classification analyses, on p.20, and in Figure 3, it is not particularly clear if and when you are using absolute or relative power measures. It’s described, for example, as “beta and gamma band power, as either an absolute or relative metric", but which is used in the main analyses is not clearly indicated.

- The acronym WASO is used (starting page 8), but never spelled out

- There are a couple typos:

o Figure 6 legend: “Peason” should be “Pearson"

o p. 27 “broadly point to age-related flattening of slopes across,"across seems to be an extra word here

- The supplemental materials reference articles beyond what is listed in the reference list of the main article, but I couldn’t find these references listed anywhere

Reviewer 2:

The authors set out to replicate and extent recent findings that indicated that the spectral slope reflects an electrophysiological marker of arousal in human sleep in a large dataset comprising 6 independent datasets and 11000 subjects. Moreover, they evaluated potential sources of variation of the spectral slope within and between subjects such as age and gender and systematically evaluated potential confounds.

The manuscript addresses a timely issue with a large sample with high statistical power enabling to disentangle the influence of variables such as age. With strict inclusion criteria and in a methodological and thorough way, the authors work their way through potential confounds such as EMG or reference schemes. Overall, the manuscript is clearly written and provides other researchers with a valuable resource to navigate the analysis of the EEG spectral slope.

A few queries remain and several issues need to be addressed:

1. (P4 and P26) The authors state that “Under this model, more synchronized activity results in steeper (more negative) 1/f slopes” and “the spectral slope can be interpreted to reflect the degree of local synchronous neural activity”. However, this wording is confusing as in the literature they cite (e.g. Gao 2017, Donoghue 2020, He 2014), the slope is often referred to as a measure of asynchronous/ non-oscillatory activity. Although there certainly is a relationship between synchronous and asynchronous activity, it is commonly assumed that true periodic brain activity would occur as “bumps” above 1/f drop-off of the PSD (paper see above).

2. (P10) The authors should note that, power estimates (and with them slope estimates) can be inflated when the mean is taken first as in log10(mean(X))) (Izhekevich, Voytek biorxiv). Thus, we might expect suboptimal (and not comparable) results for this calculation. Using mean(log10(X)) or log10(median(X)) mitigated the overestimation issues.

3. (P20) As the authors noted, Lendner et al. used N3 sleep in the LDA to discriminate between sleep states, a state which is characterized by SO. Therefore, if the LDA discrimination between N3 and wake is comparable between SO and slope this means the slope is as helpful as SO to distinguish between states. Moreover, the good LDA performance of the slope in the differentiation between wake and REM, makes it a valuable (additional) marker. Here, the authors use N2 sleep and beta/gamma power for their LDA, therefore their results are not directly comparable with Lendner et al.

Moreover, the use of the beta range (15-30 Hz) might be influenced by the occurrence of spindles which are a typical characteristic of N2 sleep (12-16 Hz). Thus, a better discrimination of slope vs beta power in R/N2 comparison supports an additional use of the spectral slope. In addition, it makes sense that beta power (which might contain some spindle power) outperforms the slope in the wake/N2 differentiation. Furthermore, the overlap in the frequency range as well as the absence of oscillations in the gamma range could potentially explain an overlap in LDA performance. The authors should revisit their interpretation of the results and could complement it with an analysis of unique explained variance to better compare the markers. In addition, the authors could compare the LDA performance when all three states are included in the classification (W, R, N2). This might be a better indicator of overall benefit of the markers for between state classifications.

4. (P22) The authors state that “The spectral slope was not independent of absolute (or relative) power across the spectrum.” As the slope is calculated from the power spectrum, how could these markers be independent? Please discuss.

5. (P26) This comment also pertains comment 2. The authors compute inter-hemispheric coherence in the gamma range. First, I was not able to find where in the methods or in the supplements the authors reveal how exactly the coherence was calculated. If not yet included, please add. Moreover, coherence measures are also influenced by referencing scheme (e.g. Bastos 2016) and volume spread (Nunez et al 1997 1999), power (Nolte 2004, as the results from the current study also revealed). With only two available sensors and a common reference (LM) a potential influence on coherence estimation seems plausible. Regarding power, if REM sleep exhibits the lowest gamma power, coherence should be smallest in REM (ref). If the authors are interested in coherence, other measures such as described by Hipp 2011 (orthogonalized power correlations), Nolte 2004 (imaginary coherence) or mutual information (Quian Quiroga and Panzeri, 2009) might be better suited. Overall, the coherence results do not add much to the story of the manuscript (sources of variability of spectral slope). The authors should either explain their rationale a bit more in details or consider moving the coherence results to the supplements.

6. (P32) The authors state in the discussion that they “reported novel differences in other aspects of the state-specific slope distributions, including its variability”. However, Lendner et al showed a higher variability in wakefulness compared to quiescent rest (Fig.2 Supp 1), possibly due to the fact that (same as in the current datasets) analyzed wake periods occurred shortly before sleep or as arousals during the night when patients were drowsy. Moreover, Hill et al 2021 reported a change of spectral slope during early to middle childhood. Therefore, the author might want to consider revisiting their novelty claim.

7. (P33) Regarding their model, please refer to literature about knee frequencies and neuronal timescales (e.g. Gao 2020, Miller 2009, He 2010).

Minor:

P4 L1 - there is still (ref) in brackets

P4 - second to last line - maybe include C3/C4 after “central scalp EEG channels"

P8 - please explain abbreviation WASO

P8 - please introduce QC as abbreviation

P23 - still (ref) in brackets

Fig 1.: Please add errorbars for PSD (e.g. SEM shading) and errorbars around slope mean. Please space single subjects like in Fig. 3. Although the depiction of the PSD for each state enables comparison between studies, it is hard to compare between states. I would recommend to put W, R, NR within a panel and show them in log-log space (where the spectral slope is calculated) for an easier visual comparison. Otherwise it depicts exactly the same as row 1, Fig. 4.

Fig. 2: please add errorbars to mean, please space single subjects.

Fig. 3: Please add the results for the gamma range.

Fig. 4: Again please add errorbars.

Fig. 5: Row 2 and 4, add errorbars.

Fig, 6: Errorbars are missing.

Fig. 8: errorbars

Fig. 9: errorbars, if referring to a,b,c subpanels in text, please add letters to figures

Overall, the manuscript is well written and is an extensive and methodological sound analysis of sources of variation of the spectral slope. I am sure it will be of great interest to readers from diverse scientific backgrounds that are interested in evaluating the spectral slope as a potential biomarker. For instance, their results of a flattening of the REM slope with aging are promising and open up a line for further research into age-related cognitive decline.

Overall, this paper constitutes and important replication and extension and I am looking forward to seeing thus published.

## Author Response

Computational Neuroscience Model Code Accessibility Comments for Author (Required):

Please be sure to include a statement in the Materials and Methods section, under the heading “Code Accessibility", indicating whether and how the code can be accessed, including any accession numbers or restrictions, as well as the type of computer and operating system used to run the code

We have added the appropriate lines to the Data and Code Accessibility section of Materials and Methods on p.13.

Synthesis Statement for Author (Required):

The reviewers and I agree that this manuscript is useful, and timely, and that the study was well-executed. Both reviewers had some relatively minor suggestions which will improve the manuscript, and should be addressed, see below:

Thank you: we value the feedback and have made several changes to the manuscript to address these suggestions. Please, note that the figures and tables and their references have been changed to accommodate “no supplemental material policy” of eNeuro.

Reviewer 1:

In this paper, the authors do a deep dive investigating best practices for measuring the spectral slope in sleep EEG data, and evaluate the findings from pursuing this analysis across multiple large datasets. I find this investigation to be useful, important, extremely thoroughly executed, and to reflect a very well done project. Overall, I wish to commend the authors, both for doing this kind of project - digging deep into potential methodological issues and doing due diligence to validated their findings across such large datasets - and for executing it so well. I find nothing in this paper with which I take issue, and see, to my eyes, no issues with any of the analyses or main interpretations in this paper.

I have two minor comments that could potentially be noted in this paper:

1. One comment I had as I read the article was whether the authors examined any goodness-of-fit measures of the spectral slope estimates in their analyses. This is often examined, for example, in terms of the r-squared fit between the fit line and the underlying data. This is used, for example, in the Lendner paper, including in some of the analyses they do comparing different methods and pre-processing, which are conceptually similar to some of the analyses in this paper. I don’t see any goodness-of-fit measures in this manuscript. I wonder if this is something that the authors explored at all? I understand that this paper employs stringent quality control procedures, and it is not that I generally doubt that the quality of the fits here. In general, however, I do think this kind of control check can be useful for these kinds of analyses, that could perhaps be noted if it is something the authors explored, and/or perhaps simply mentioned in the Discussion section, if this is something the authors which to note as a potential recommendation for other work applying these measures.

Upon the reviewer’s excellent suggestion, we investigated the goodness-of-fit in slope across all samples. On average it was high (R2 > 0.7 for all stages and cohorts), but markedly lower for wake than sleep (R2 = 0.72 in W versus R2= 0.98 in N2 and R2 = 0.98 in R). This is consistent with the caveat stated in the Summary section of the original manuscript: “... wake periods were often noisy ... findings that point to attenuated effects during wake (namely test-retest reliability and age-related flattening) should be interpreted with this caveat in mind.” This is also consistent with the originally reported higher within-individual (epoch-wise) variability in the waking slope, compared to NR and R sleep (original Figure 6, third row). In the revised manuscript, we now report these goodness-of-fit metrics (p.21), and reference them in discussing (potential issues with) the wake data.

Second, we repeated the main analysis including only individuals with high (R2 > 0.9) goodness-of-fit metrics for all stages (retaining 38% of the full sample), which yielded the same pattern of changes in the mean slope between W, NR and R (see figure below). We now mention this in the manuscript on p. 21.

2. The second comment I had is regarding the chosen frequency range and how to fit it. This paper analyzes the 30-45 Hz range, and does so with a robust linear fit, following the Lendner paper. However, in this paper the simulations also note that a “variable slope model", reflecting power spectra with multiple exponents and a knee, seems to explain the overall changes in the spectrum. Given this, do the authors think that future work should perhaps investigate using different methods and fitting ranges to, for example, fit a broader range with a model that includes the knee? This is now available in other fitting approaches, such as the spectral parameterization method (Donoghue et al, 2020), which can parameterize knees as well as extend to broader frequency ranges while controlling for any oscillatory peaks. Alternatively, are there reasons to prefer the 30-45 Hz range as is the focus here? For this comment, I don’t expect or think that the paper needs additional analyses applying different ranges and models, but it was a question left in my mind that could perhaps be addressed in the discussion section, in particular in terms of thinking about the implications of the simulation results, and whether the authors recommend that other fitting methods and models could and should be applied in future work, and/or if the recommendation would be to stick with the exact measures used here.

Although our original focus was to provide a well-powered replication of Lendner et al, explicitly adopting their analytic approach to reduce sources of “methodological variability” (and also noting that Lendner et al extensively assessed the impact of frequency range), we agree that this is an important question (albeit not necessarily within the scope of the current manuscript). To partially address this, we adopted a second analytic approach to estimate the spectral slope (IRASA, Wen & Liu, 2016), that was specifically developed to eliminate periodic components from spectral estimates of neurophysiological signals, and thereby allow for unbiased estimation of the slope in frequency ranges known to include oscillatory activity (peaks). Although this approach does not model possible knees in the slope, which is beyond the scope of the current work, it does allow for estimation of a single slope over a broader range of frequencies, i.e. by attenuating bias from oscillatory peaks.

Using IRASA to derive slopes from the aperiodic components of power spectra, we investigated three frequency ranges: 1) 30-45 Hz, to validate the results of our original analysis, 2) 1-30 Hz, including known oscillatory peaks, e.g. sigma, and 3) 5-30 Hz, to remove slow wave activity. These auxiliary analyses were performed only in the MrOS cohort, chosen due to the higher sampling frequency (256 Hz vs 128 Hz for most other studies) which was necessary for optimal IRASA performance, focusing on C3 referenced to linked mastoids.

For the first application of IRASA (in the same 30-45 Hz range), we confirmed the spectral slope pattern of W > NR > R as our original analysis (although noting that while targeting 30-45 Hz, due to resampling up to a factor of h = 2.0, IRASA also includes information between 15 and 90 Hz). This first result suggests that our original analysis was not simply reflecting unaccounted-for periodic components.

The two other applications of IRASA including < 30 Hz frequencies did not yield the same NR > R pattern, however. Assuming that these differences are not driven by oscillatory activity (i.e. as per the premise of IRASA), these results are indeed consistent with our simulations, which pointed to the likelihood of variable slopes across the frequency spectrum. Indeed, inspection of our original Figure 6 implied that during R, people with steeper 30-45 Hz slopes tended to have less steep 10-30 Hz slopes. Analyses that estimate a single slope across a large frequency range may therefore be difficult to interpret. We agree with the reviewer’s general comments and have added text to the Discussion to highlight some of these issues: i.e. even accounting for oscillatory activity, that (roughly speaking) < 30 Hz activity shows sources of variation in the spectral slope that are distinct from > 30 Hz, and particularly during R. We also have added this figure as a part of Figure 3-1b and briefly describe the results on p 27.

Minor description notes:

3. In the classification analyses, on p.20, and in Figure 3, it is not particularly clear if and when you are using absolute or relative power measures. It’s described, for example, as “beta and gamma band power, as either an absolute or relative metric", but which is used in the main analyses is not clearly indicated.

We have modified the legend of the Figure 3 (Figure 4 in the revised version) stating that the results are presented for absolute beta power.

4. The acronym WASO is used (starting page 8), but never spelled out

The explanation for WASO abbreviation has been added on p. 7.

5. There are a couple typos:

- Figure 6 legend: “Peason” should be “Pearson"

- p. 27 “broadly point to age-related flattening of slopes across,"across seems to be an extra word here

Thank you for pointing out to those typos. They now have been fixed.

6. The supplemental materials reference articles beyond what is listed in the reference list of the main article, but I couldn’t find these references listed anywhere

Due to eNeuro policy of no supplementary material, we have restructured the manuscript and incorporated the most important information from supplementary material in the main text. All references are now listed in the references section of the main text.

Reviewer 2:

The authors set out to replicate and extent recent findings that indicated that the spectral slope reflects an electrophysiological marker of arousal in human sleep in a large dataset comprising 6 independent datasets and 11000 subjects. Moreover, they evaluated potential sources of variation of the spectral slope within and between subjects such as age and gender and systematically evaluated potential confounds.

The manuscript addresses a timely issue with a large sample with high statistical power enabling to disentangle the influence of variables such as age. With strict inclusion criteria and in a methodological and thorough way, the authors work their way through potential confounds such as EMG or reference schemes. Overall, the manuscript is clearly written and provides other researchers with a valuable resource to navigate the analysis of the EEG spectral slope.

A few queries remain and several issues need to be addressed:

1. (P4 and P26) The authors state that “Under this model, more synchronized activity results in steeper (more negative) 1/f slopes” and “the spectral slope can be interpreted to reflect the degree of local synchronous neural activity”. However, this wording is confusing as in the literature they cite (e.g. Gao 2017, Donoghue 2020, He 2014), the slope is often referred to as a measure of asynchronous/ non-oscillatory activity. Although there certainly is a relationship between synchronous and asynchronous activity, it is commonly assumed that true periodic brain activity would occur as “bumps” above 1/f drop-off of the PSD (paper see above).

Agreed. To avoid the confusion between large-scale oscillatory and local synchronization we have removed those statements.

2. (P10) The authors should note that, power estimates (and with them slope estimates) can be inflated when the mean is taken first as in log10(mean(X))) (Izhekevich, Voytek biorxiv). Thus, we might expect suboptimal (and not comparable) results for this calculation. Using mean(log10(X)) or log10(median(X)) mitigated the overestimation issues.

In the original Methods section on p 10, we reviewed three approaches for spectral slope estimation and provided the reasoning for our choice for main analysis, and made the above point about the likely suboptimal properties of log(mean(X)). From the original paper (bold added for emphasis):

"Power spectra were estimated individually for each epoch. To derive the spectral slope, we averaged log-scaled spectra, and then calculated a single slope estimate. As a sensitivity analysis, we also calculated two other estimates: i) averaging untransformed spectra, then taking the log and estimating the slope; alternatively, ii) averaging slopes calculated per epoch on the log-transformed spectra. That is, the three approaches can be labelled: slope(mean(log(X))), slope(log(mean(X))) and mean(slope(log(X))) respectively, where X are the epoch-level power spectra. An advantage of the third approach is that it also provides a convenient estimate of the variability in slope across epochs. We expect qualitatively similar results from all approaches, although the second approach may be more sensitive to outlier epochs not otherwise filtered out. Work on the resting state eyes open/closed alpha rhythm differences suggested that log-transforming power at the epoch level prior to averaging was optimal in that context 23. Based on an initial analysis in the CFS cohort, all three metrics were effectively equivalent, both in terms of correlation with each other (r > 0.95) and the magnitude of tests of mean differences between stages (data not shown). The one exception was that slope(log(mean(X))) was less correlated with the other two metrics during wake (here r ∼0.7), which presumably reflects a greater sensitivity to noisy/outlier epochs that occur more often during wake. As noted, our final analyses were based on slope(mean(log(X))), although we do not expect any substantive differences in results if these alternatives were used. “

3. (P20) As the authors noted, Lendner et al. used N3 sleep in the LDA to discriminate between sleep states, a state which is characterized by SO. Therefore, if the LDA discrimination between N3 and wake is comparable between SO and slope this means the slope is as helpful as SO to distinguish between states. Moreover, the good LDA performance of the slope in the differentiation between wake and REM, makes it a valuable (additional) marker. Here, the authors use N2 sleep and beta/gamma power for their LDA, therefore their results are not directly comparable with Lendner et al.

Moreover, the use of the beta range (15-30 Hz) might be influenced by the occurrence of spindles which are a typical characteristic of N2 sleep (12-16 Hz). Thus, a better discrimination of slope vs beta power in R/N2 comparison supports an additional use of the spectral slope. In addition, it makes sense that beta power (which might contain some spindle power) outperforms the slope in the wake/N2 differentiation. Furthermore, the overlap in the frequency range as well as the absence of oscillations in the gamma range could potentially explain an overlap in LDA performance. The authors should revisit their interpretation of the results and could complement it with an analysis of unique explained variance to better compare the markers. In addition, the authors could compare the LDA performance when all three states are included in the classification (W, R, N2). This might be a better indicator of overall benefit of the markers for between state classifications.

Our primary objective in performing the epoch-based stage classification was to test the more general question of whether the spectral slope was superior relative to other commonly used spectral metrics, rather than replicate the specific contrasts presented by Lendner et al.

For transparency, however, below we present an extended analysis that includes absolute power across all classical frequency bands for binary classifications between wake, N2, N3 and REM epochs. Further, we also present goodness of fit (R2) metrics from linear models based on all stages for a particular spectral metric (i.e. metric ∼ stage [W, N2, N3, R] + error).

We confirmed our primary conclusion that while spectral slope exhibits optimal performance in W vs REM classification, it is substantively similar to beta and gamma power. For other contrasts, the spectral slope is the least effective in such task (e.g. N2 vs N3). We have added this as a supplementary figure 4-1

4. (P22) The authors state that “The spectral slope was not independent of absolute (or relative) power across the spectrum.” As the slope is calculated from the power spectrum, how could these markers be independent? Please discuss.

Agreed. The spirit of this sentence was a (misguided) attempt to introduce the topic of the section (by restating a near-obligatory definitional fact), rather than an attempt to present a novel result. We have changed the wording to reflect this:

"As expected, the spectral slope was not independent of absolute (or relative) power across the spectrum.”

5. (P26) This comment also pertains comment 2. The authors compute inter-hemispheric coherence in the gamma range. First, I was not able to find where in the methods or in the supplements the authors reveal how exactly the coherence was calculated. If not yet included, please add. Moreover, coherence measures are also influenced by referencing scheme (e.g. Bastos 2016) and volume spread (Nunez et al 1997 1999), power (Nolte 2004, as the results from the current study also revealed). With only two available sensors and a common reference (LM) a potential influence on coherence estimation seems plausible. Regarding power, if REM sleep exhibits the lowest gamma power, coherence should be smallest in REM (ref). If the authors are interested in coherence, other measures such as described by Hipp 2011 (orthogonalized power correlations), Nolte 2004 (imaginary coherence) or mutual information (Quian Quiroga and Panzeri, 2009) might be better suited. Overall, the coherence results do not add much to the story of the manuscript (sources of variability of spectral slope). The authors should either explain their rationale a bit more in details or consider moving the coherence results to the supplements.

Agreed. We now present the coherence analysis results as a supplemental figure 7-1.

6. (P32) The authors state in the discussion that they “reported novel differences in other aspects of the state-specific slope distributions, including its variability”. However, Lendner et al showed a higher variability in wakefulness compared to quiescent rest (Fig.2 Supp 1), possibly due to the fact that (same as in the current datasets) analyzed wake periods occurred shortly before sleep or as arousals during the night when patients were drowsy. Moreover, Hill et al 2021 reported a change of spectral slope during early to middle childhood. Therefore, the author might want to consider revisiting their novelty claim.

We have removed the word “novel” from the quoted line: “...reported differences in other aspects of the state-specific slope distributions, including its variability...”. Further, we now reference Hill et al. (2021) (which was not published when we drafted the original manuscript).

7. (P33) Regarding their model, please refer to literature about knee frequencies and neuronal timescales (e.g. Gao 2020, Miller 2009, He 2010).

We have added these references as suggested.

Minor:

P4 L1 - there is still (ref) in brackets

Fixed.

P4 - second to last line - maybe include C3/C4 after “central scalp EEG channels"

We have added C3/C4 as suggested.

P8 - please explain abbreviation WASO

The explanation for WASO abbreviation has been added on p. 7.

P8 - please introduce QC as abbreviation

The explanation for QC abbreviation has been added on p. 8.

P23 - still (ref) in brackets

This have now been fixed.

Fig 1.: Please add errorbars for PSD (e.g. SEM shading) and errorbars around slope mean. Please space single subjects like in Fig. 3. Although the depiction of the PSD for each state enables comparison between studies, it is hard to compare between states. I would recommend to put W, R, NR within a panel and show them in log-log space (where the spectral slope is calculated) for an easier visual comparison. Otherwise it depicts exactly the same as row 1, Fig. 4.

Following the reviewer’s suggestion, we have reoriented Figure 1 to place stages (W, NR & R) within the same panel for a given study.

We address all comments related to error bars (below) in this response. Our strategy for key metrics of interest (i.e. spectral slope observed in W, NR and R) was to plot original, individual-level data (i.e. original Figure 1b, Figure 2a, Figure 3 and Figure 10), i.e. to show full distributions rather than rely on summary statistics. Given the large numbers of subjects in each cohort, a) not all forms of graphical representation are viable, and/or b) the standard errors are typically vanishingly small. For example, here are panels from Figure 1 with 95% confidence intervals included through shading:

We feel that including these plots (which do not address the core hypotheses tested related to spectral slope in any case), is more likely to confuse readers, and adds no meaningful information. Beyond means, not all metrics have readily available general closed forms for their standard errors (e.g. the between individual standard deviations in Figure 4 [Figure 5 in current version of the manuscript]), without resorting to bootstrap or jackknife methods. We note that Figure 5 [Figure 6 in current version of the manuscript] top and third rows reflect box-and-whisker plots showing interquartile ranges and outliers, not SEM/CI intervals.

We are not sure what ’space single subjects’ means (especially in the context of cohorts with 1000s of subjects) but suggest that the individual-level data-points (translucent gray lines) related to slope might convey the equivalent information.

Fig. 2: please add errorbars to mean, please space single subjects.

Please see above.

Fig. 3: Please add the results for the gamma range.

We have now added gamma range results (as well as in other classical bands) in supplementary figure 4-1.

Fig. 4: Again please add errorbars.

Please see above.

Fig. 5: Row 2 and 4, add errorbars.

Please see above.

Fig, 6: Errorbars are missing.

Please see above.

Fig. 8: errorbars

Please see above.

Fig. 9: errorbars, if referring to a,b,c subpanels in text, please add letters to figures

Please see above. (it is a supplementary figure 7-1 now)

Overall, the manuscript is well written and is an extensive and methodological sound analysis of sources of variation of the spectral slope. I am sure it will be of great interest to readers from diverse scientific backgrounds that are interested in evaluating the spectral slope as a potential biomarker. For instance, their results of a flattening of the REM slope with aging are promising and open up a line for further research into age-related cognitive decline.

Overall, this paper constitutes and important replication and extension and I am looking forward to seeing thus published.

References

Wen, H., Liu, Z. Separating Fractal and Oscillatory Components in the Power Spectrum of Neurophysiological Signal. Brain Topogr 29, 13-26 (2016). https://doi.org/10.1007/s10548-015-0448-0
