# Supplementary material for: Sources of Variation in the Spectral Slope of the Sleep EEG
Source: eNeuro. 2022 Sep 21;9(5):ENEURO.0094-22.2022. doi: 10.1523/ENEURO.0094-22.2022 (PMC9512622; doi:10.1523/ENEURO.0094-22.2022)
Supplement: Extended Data Table 1-1 — Pre-QC and post-QC sample sizes and durations of sleep state by cohort. For each cohort, the average duration of each state, following the initial round of QC (based on removing individuals without sufficient duration (at least 10 minutes) after performing various epoch-level exclusions (QC1), e.g. only retaining epochs flanked by similarly staged epochs, rejecting epochs with annotated arousals or respiratory events, as well as signal outliers, etc. The second round of QC (QC2) excluded individuals based upon statistical properties of the derived metrics (e.g. power, slope). See Methods for details. These procedures were collectively designed to be stringent: that they removed large proportions of some cohorts for the final analysis more reflects the choices of QC rather than inherent issues with the data (i.e. many childhood recordings were removed due to low rates of WASO, as we excluded leading and trailing wake epochs from all recordings, but required all studies to have sufficient duration of wake as well as sleep epochs). No SHHS individuals were retained in the linked mastoid dataset, as it was not possible to re-reference the hardwired contralateral mastoid channels; also see ‘Technical factors in the SHHS datasets’ in Results for other technical issues encountered in the SHHS cohorts. Download Table 1-1, DOC file. [file enu-eN-NWR-0094-22-s25.doc]

|  |  |  |  |  |  |  |  |  |  |  |  |  |  |  |  |  |  |  |  |  |
| --- | --- | --- | --- | --- | --- | --- | --- | --- | --- | --- | --- | --- | --- | --- | --- | --- | --- | --- | --- | --- |
|  |  | Average minutes per stage (post QC1) | | | | |  | Proportion of individuals with at least 10 minutes | | | | |  | *Sample sizes* |  | *Contralateral mastoids* | |  | *Linked mastoids* | |
| **Cohort** |  | **N1** | **N2** | **N3** | **R** | **W** |  | **N1** | **N2** | **N3** | **R** | **W** |  | **Original** |  | **Post QC2** | **% retained** |  | **Post QC2** | **% retained** |
|  |  |  |  |  |  |  |  |  |  |  |  |  |  |  |  |  |  |  |  |  |
| CCSHS |  | 4.2 | 154.7 | 81.9 | 60.8 | 25.1 |  | 36.7% | 99.8% | 100.0% | 99.8% | 74.8% |  | 515 |  | 288 | 55.9% |  | 276 | 53.6% |
| CFS |  | 4.6 | 105.8 | 64.8 | 35.8 | 52.4 |  | 18.5% | 98.2% | 91.6% | 89.3% | 89.2% |  | 730 |  | 440 | 60.3% |  | 370 | 50.7% |
| CHAT(BL) |  | 8.3 | 118.7 | 111.0 | 50.3 | 20.4 |  | 72.3% | 99.8% | 99.8% | 99.3% | 54.4% |  | 453 |  | 163 | 36.0% |  | 187 | 41.3% |
| CHAT(FU) |  | 7.5 | 131.1 | 109.4 | 55.1 | 17.2 |  | 64.7% | 100.0% | 100.0% | 99.3% | 60.7% |  | 407 |  | 164 | 40.3% |  | 151 | 37.1% |
| CHAT(NR) |  | 9.9 | 123.5 | 110.3 | 55.8 | 22.4 |  | 73.9% | 100.0% | 99.9% | 98.4% | 67.8% |  | 779 |  | 309 | 39.7% |  | 283 | 36.3% |
| MrOS1 |  | 4.6 | 94.9 | 30.8 | 33.3 | 73.3 |  | 14.4% | 99.5% | 70.4% | 95.3% | 99.0% |  | 2907 |  | 2353 | 80.9% |  | 2146 | 73.8% |
| MrOS2 |  | 9.6 | 95.8 | 37.7 | 38.5 | 75.9 |  | 41.0% | 99.8% | 56.2% | 96.5% | 98.3% |  | 1025 |  | 829 | 80.9% |  | 765 | 74.6% |
| SHHS1 |  | 7.0 | 84.9 | 41.2 | 24.2 | 28.1 |  | 14.4% | 98.4% | 76.5% | 84.0% | 84.5% |  | 5793 |  | 3435 | 59.3% |  | 0 | 0.0% |
| SHHS2 |  | 4.3 | 93.3 | 40.1 | 25.5 | 49.0 |  | 13.1% | 98.6% | 80.2% | 87.2% | 95.6% |  | 2647 |  | 1950 | 73.7% |  | 0 | 0.0% |
| SOF |  | 3.6 | 71.8 | 41.3 | 23.3 | 64.1 |  | 10.4% | 97.8% | 89.2% | 81.9% | 97.3% |  | 453 |  | 324 | 71.5% |  | 281 | 62.0% |
|  |  |  |  |  |  |  |  |  |  |  |  |  |  |  |  |  |  |  |  |  |
|  |  |  |  |  |  |  |  |  |  |  |  |  |  |  |  |  |  |  |  |  |
|  |  |  |  |  |  |  |  |  |  |  |  | Tot |  | **15709** |  | 10255 |  |  | 4459 |  |
|  |  |  |  |  |  |  |  |  |  |  |  | Indiv |  | **11630** |  | **7312** |  |  | **3543** |  |

**Table 1-1. Pre-QC and post-QC sample sizes and durations of sleep state by cohort.** For each cohort, the average duration of each state, following the initial round of QC (based on removing individuals without sufficient duration (at least 10 minutes) after performing various epoch-level exclusions (QC1), e.g. only retaining epochs flanked by similarly staged epochs, rejecting epochs with annotated arousals or respiratory events, as well as signal outliers, etc. The second round of QC (QC2) excluded individuals based upon statistical properties of the derived metrics (e.g. power, slope). See **Methods** for details. These procedures were collectively designed to be stringent: that they removed large proportions of some cohorts for the final analysis more reflects the choices of QC rather than inherent issues with the data (i.e. many childhood recordings were removed due to low rates of WASO, as we excluded leading and trailing wake epochs from all recordings, but required all studies to have sufficient duration of wake as well as sleep epochs). No SHHS individuals were retained in the linked mastoid dataset, as it was not possible to re-reference the hardwired contralateral mastoid channels; also see ‘Technical factors in the SHHS datasets’ in Results for other technical issues encountered in the SHHS cohorts.
