# Supplementary figures and images for: Sources of Variation in the Spectral Slope of the Sleep EEG
Source: eNeuro. 2022 Sep 21;9(5):ENEURO.0094-22.2022. doi: 10.1523/ENEURO.0094-22.2022 (PMC9512622; doi:10.1523/ENEURO.0094-22.2022)

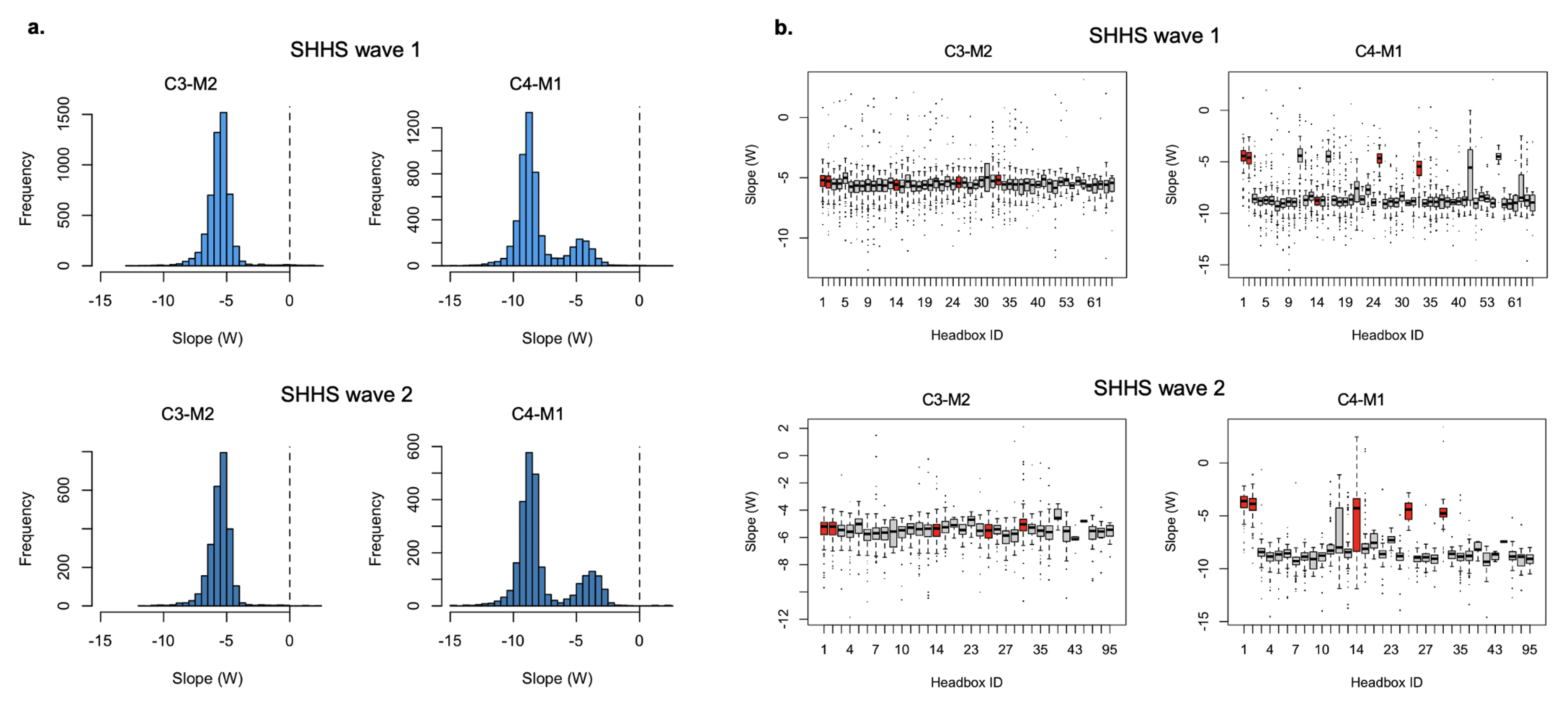

Supplement: Extended Data Figure 1-1 — Spectral slopes in SHHS. a) Histograms of W spectral slopes for C3-M2 and C4-M1, for both wave 1 and 2, which indicate a bimodal distribution for C4-M1 only. b) Mean spectral slopes (separately for C3-M2 and C4-M1 in wave 1 and 2) stratified by the ID of the physical recording device (headbox). The same units and IDs were preserved across waves 1 and 2 (albeit with fewer individuals/devices used, as well as a handful of new devices introduced for wave 2). The devices that were outliers for C4-M1 in wave 2 were also outliers in wave 1. Download Figure 1-1, TIF file. [file enu-eN-NWR-0094-22-s02.tif]

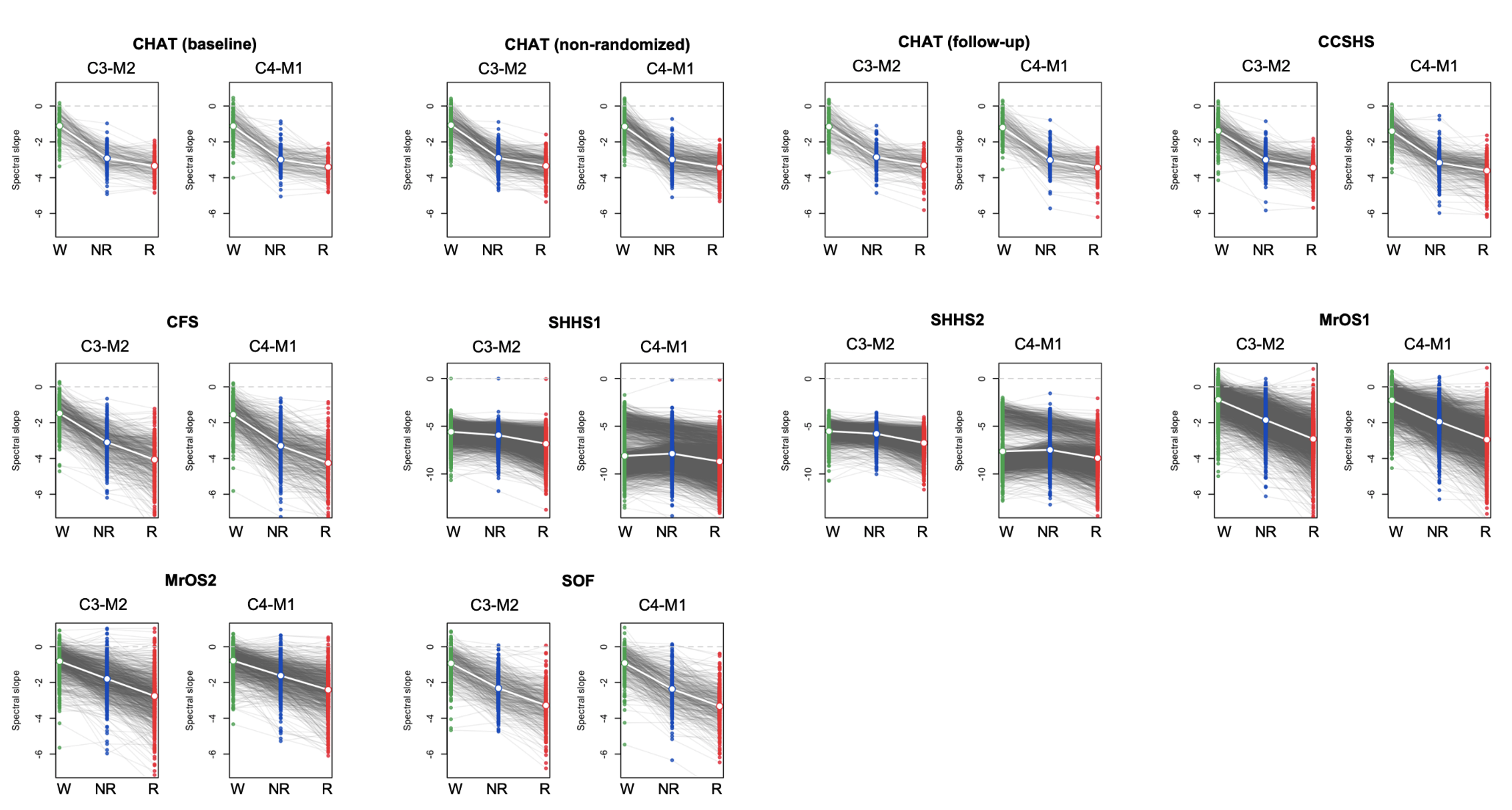

Supplement: Extended Data Figure 1-2 — EEG spectral slopes (CM-referenced dataset). See legend for Figure 1 for details: similar methods were applied to generate these plots, but here listed for both central channels, and separately for all ten samples. Green, blue and red indicate wake, NREM and REM respectively. Download Figure 1-2, TIF file. [file enu-eN-NWR-0094-22-s03.tif]

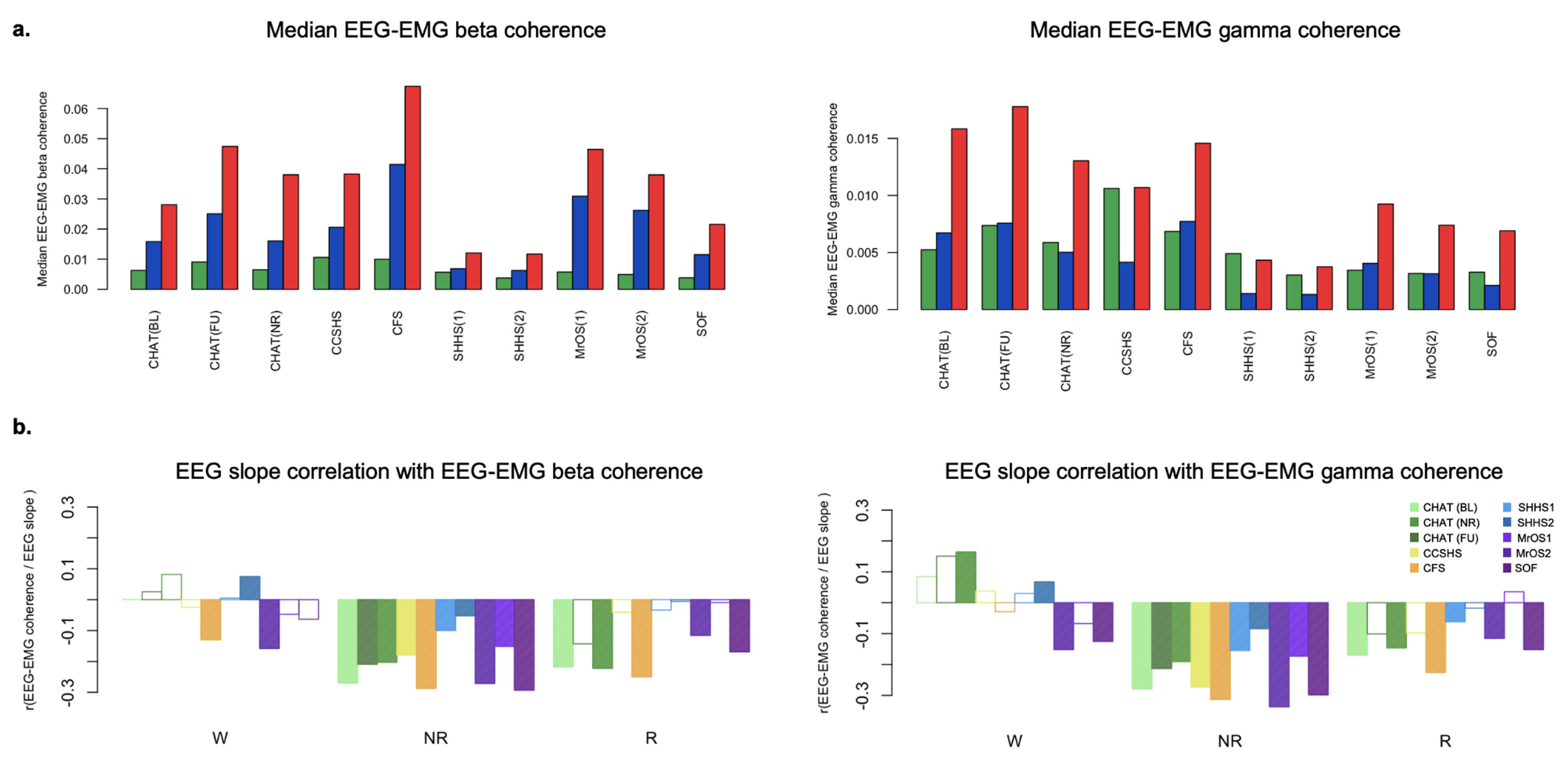

Supplement: Extended Data Figure 2-3 — EEG-EMG coherence and EEG spectral slopes. a) For beta and gamma bands, median EEG-EMG coherence stratified by state and cohort. All analyses used CM-referencing. b) Correlations between EEG slope and EEG-EMG coherence, by state and cohort. Particularly during NREM, individuals with higher EEG-EMG crosstalk (i.e. coherence) tended to have steeper slopes. Green, blue and red indicate wake, NREM and REM respectively. Download Figure 2-3, TIF file. [file enu-eN-NWR-0094-22-s04.tif]

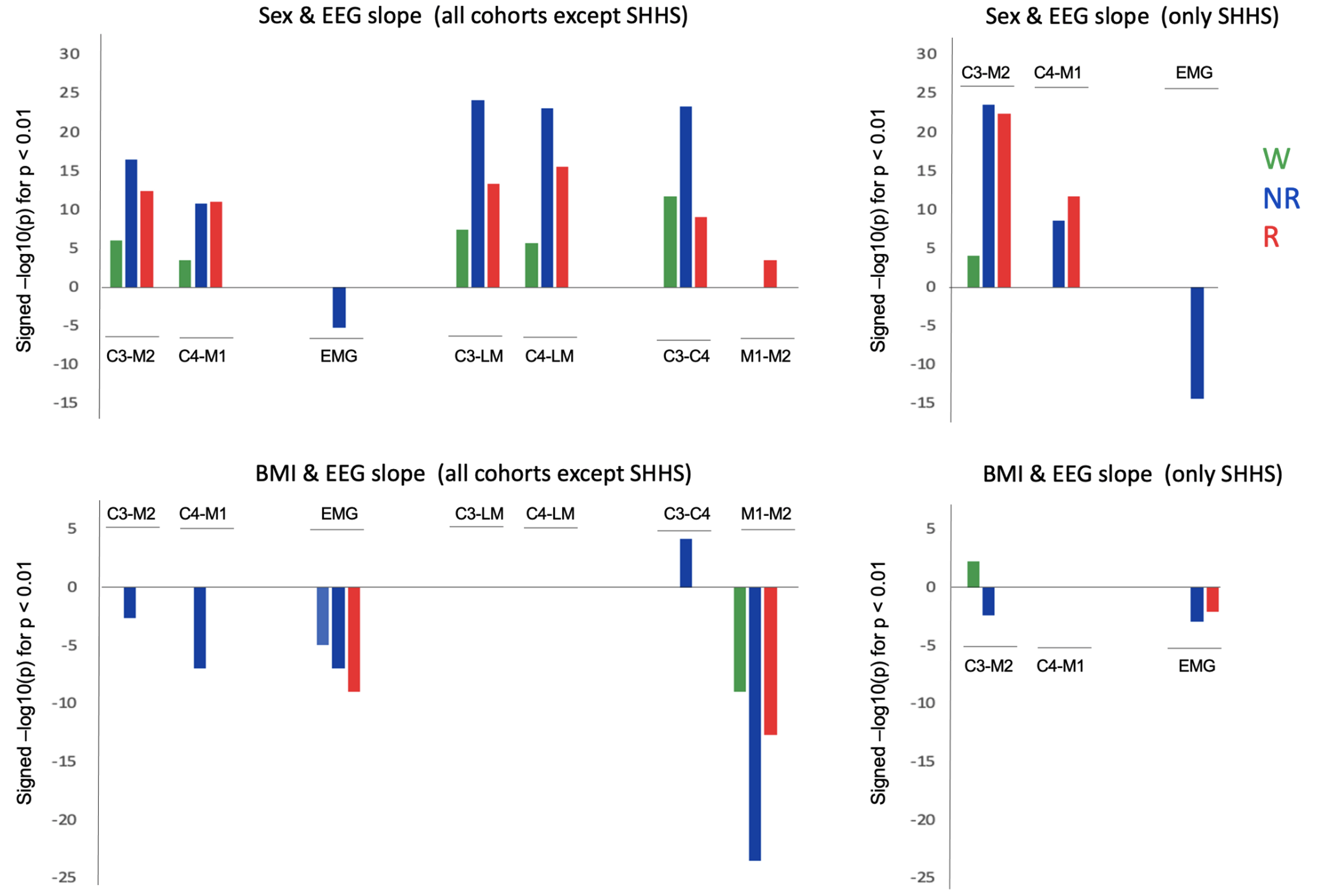

Supplement: Extended Data Figure 2-5 — Summary of sex && BMI associations with the EEG spectral slope. Results indicate the signed -log10 p-value (as an index of relative effect size) from regressions of slope on sex, BMI and other covariates (see Methods). Only associations with p<0.01 are shown. Green, blue and red indicate wake, NREM and REM respectively. Also see tables Figure 2-4 & Figure 2-6. Download Figure 2-5, TIF file. [file enu-eN-NWR-0094-22-s05.tif]

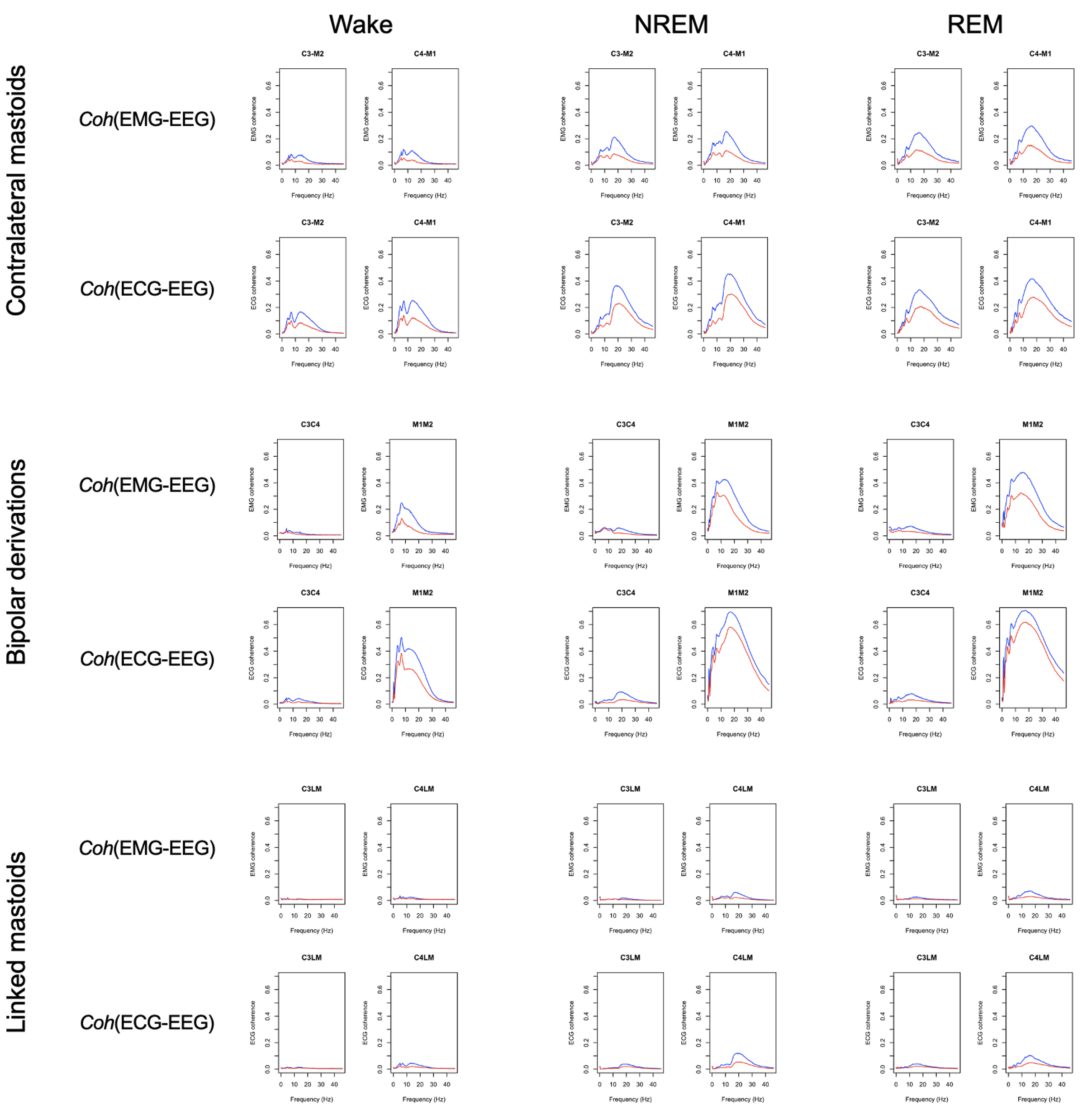

Supplement: Extended Data Figure 2-7 — Coherence between EEG and EMG/ECG in the CFS cohort. Blue/red lines indicate mean coherence for males/females. Note that the use of linked mastoid referencing appeared to reduce EMG/ECG contamination, as indexed by spectral coherence with the EEG. Download Figure 2-7, TIF file. [file enu-eN-NWR-0094-22-s06.tif]

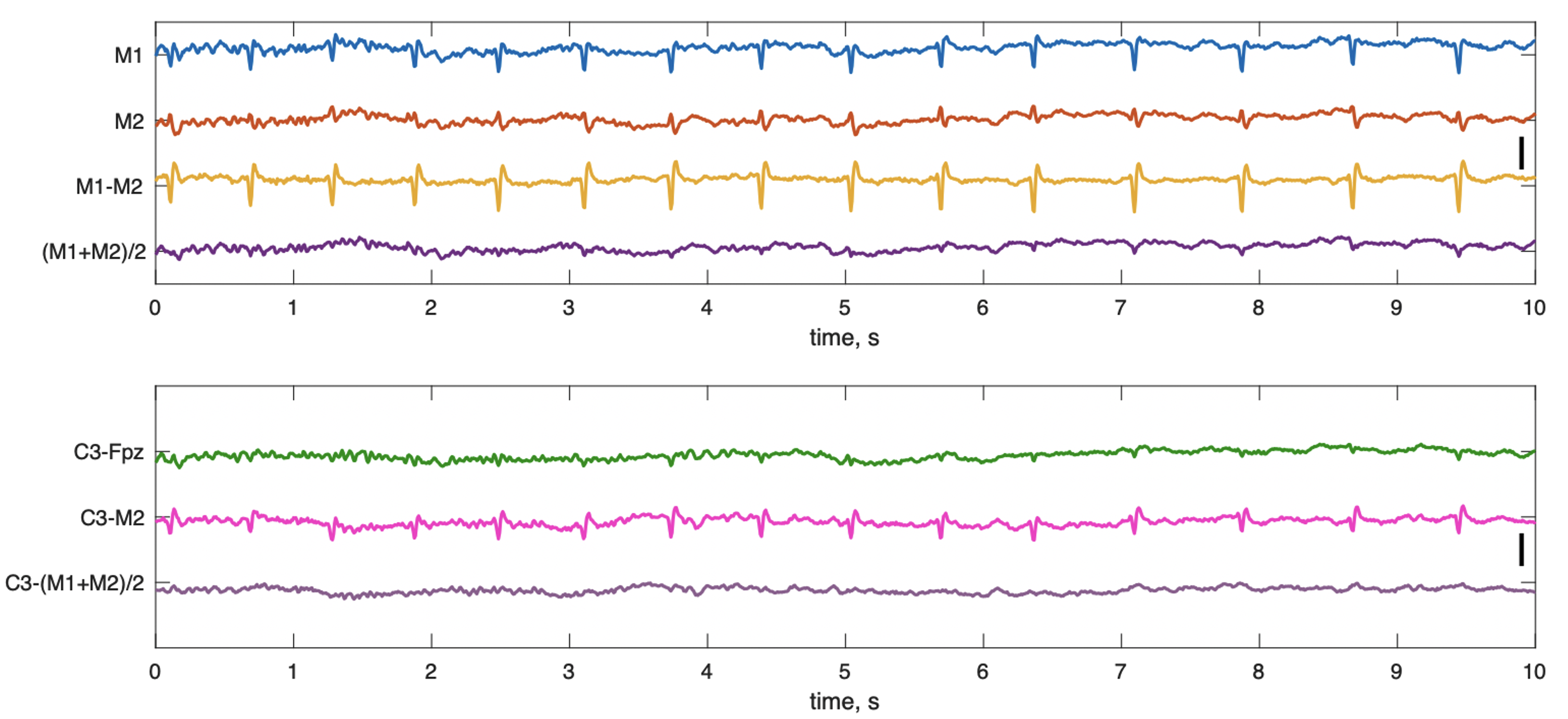

Supplement: Extended Data Figure 2-9 — Choice of reference and ECG artifacts. The upper plot illustrates 10s of N2 from M1-Fpz, M2-Fpz, M1-M2 (the ‘cross-mastoid’) and (M1+M2)/2 (linked mastoid) for a subject from CFS with extreme cardiac interference in C3-M2. Fpz was the recording reference electrode. Both M1 and M2 time series are severely affected by ECG artifacts. Due to the opposite polarity, however, the linked mastoid (M1+M2)/2 signal almost fully eliminates this issue. The bottom plot shows the C3 channel with different referencing (the recording reference Fpz, M2 and (M1+M2)/2) during the same period. Note how the contralateral mastoid reference introduced cardiac artifacts into the C3 channel; however, if C3 in fact contained strong cardiac artifacts prior re-referencing, using the contralateral mastoid would have helped to cancel it out. The vertical black bar on the left corresponds to 100 uV. Download Figure 2-9, TIF file. [file enu-eN-NWR-0094-22-s07.tif]

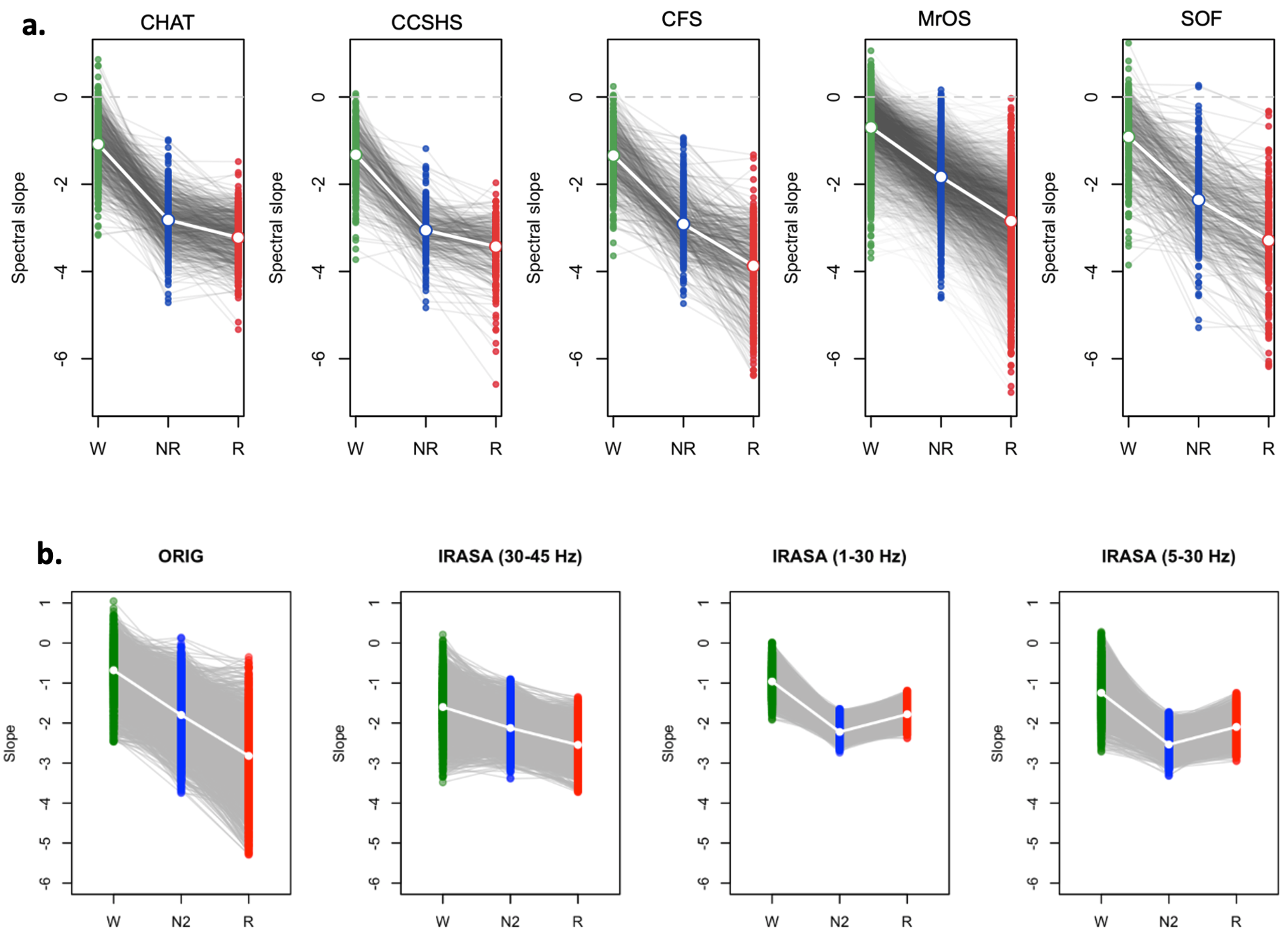

Supplement: Extended Data Figure 3-1 — EEG spectral slope for C4-LM and using IRASA method. a. See legend of Figure 3 for details: this plot provides the same analysis but for C4-LM instead of C3-LM. Green, blue and red indicate wake, NREM and REM respectively. Also see Figure 3-2. b. EEG spectral slope in MrOS cohort for C3 referenced to linked mastoids. From left to right: slopes estimated: between 30 - 45 Hz using original method; between 30 - 45 Hz using IRASA method; between 1 - 30 Hz using IRASA method; between 5 - 30 Hz using IRASA method. Download Figure 3-1, TIF file. [file enu-eN-NWR-0094-22-s08.tif]

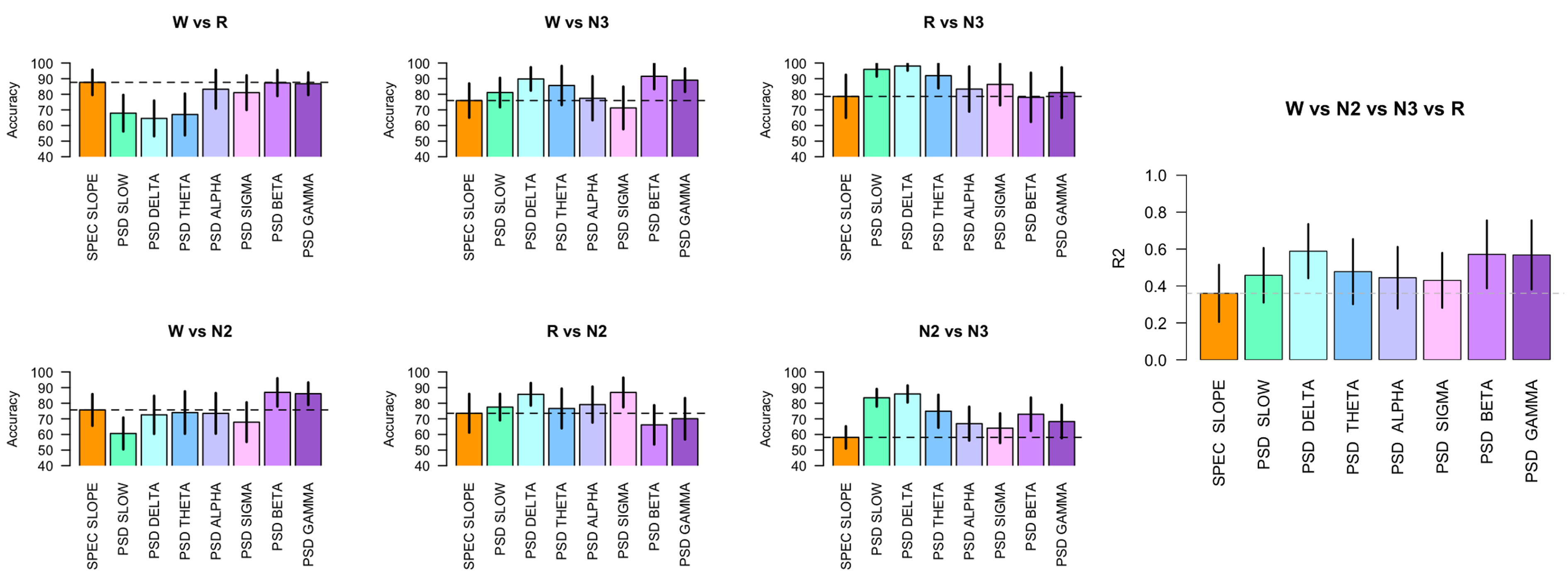

Supplement: Extended Data Figure 4-1 — State classification based on the spectral slope or absolute power across classic frequency bands. Six bar plots on the left illustrate mean accuracies across individuals of W vs R, W vs N2, W vs N3, R vs N2, R vs N3 and N2 vs N3 classification and the error bars represent standard deviation in accuracies across individuals. Dashed grey lines illustrate the performance of spectral slope. The bar plot on the right represents the average goodness of fit (R2) of a linear model based on all stages together for a particular spectral metric ( spectral metric ∼ stage [W, N2, N3, R] + error). Download Figure 4-1, TIF file. [file enu-eN-NWR-0094-22-s09.tif]

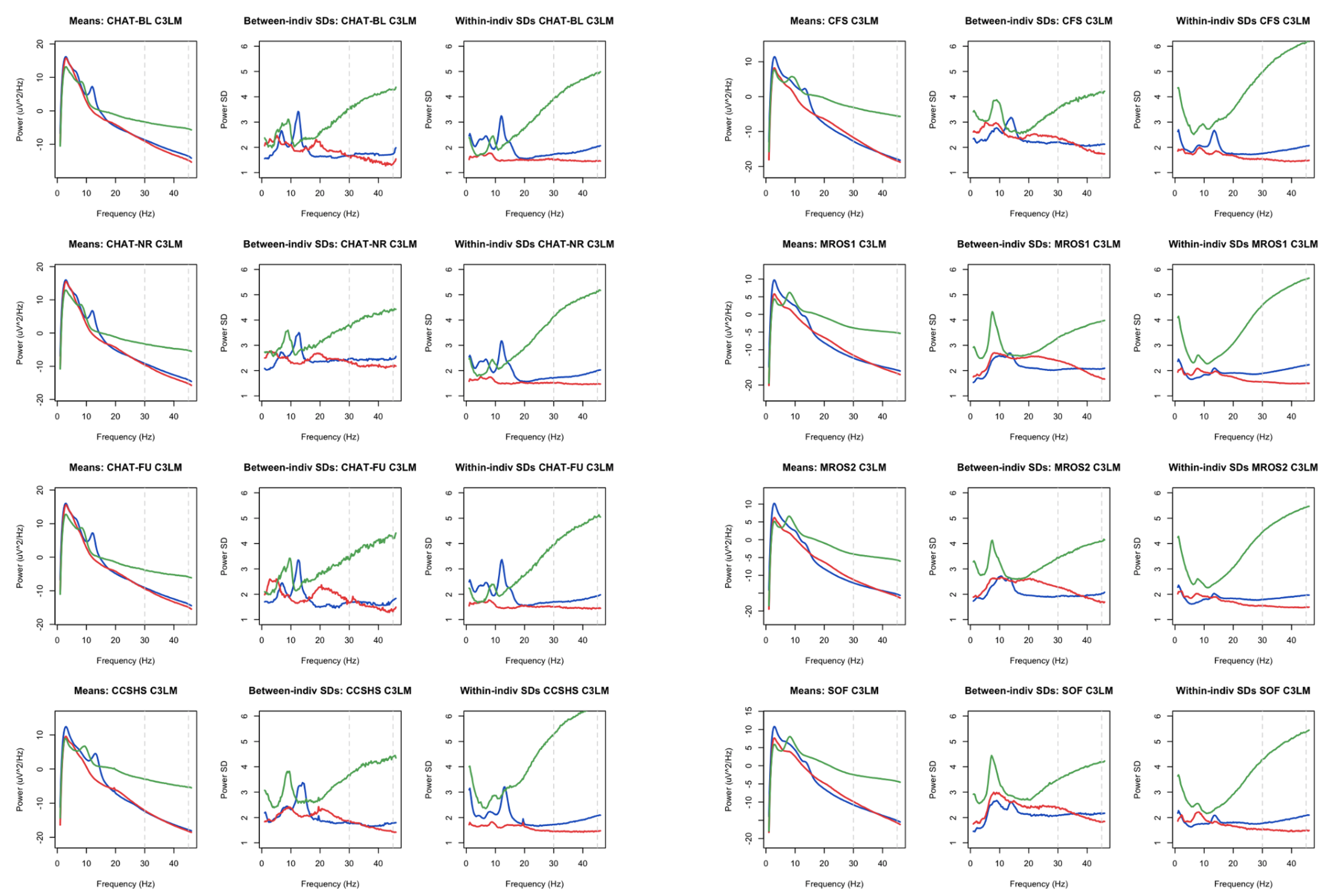

Supplement: Extended Data Figure 5-1 — Spectral power means and variability, by sleep state and cohort. See legend of Figure 5 for details: here, figures are plotted separately by cohort rather than super-imposed (as in Figure 5). Green, blue and red indicate wake, NREM and REM respectively. Download Figure 5-1, TIF file. [file enu-eN-NWR-0094-22-s10.tif]

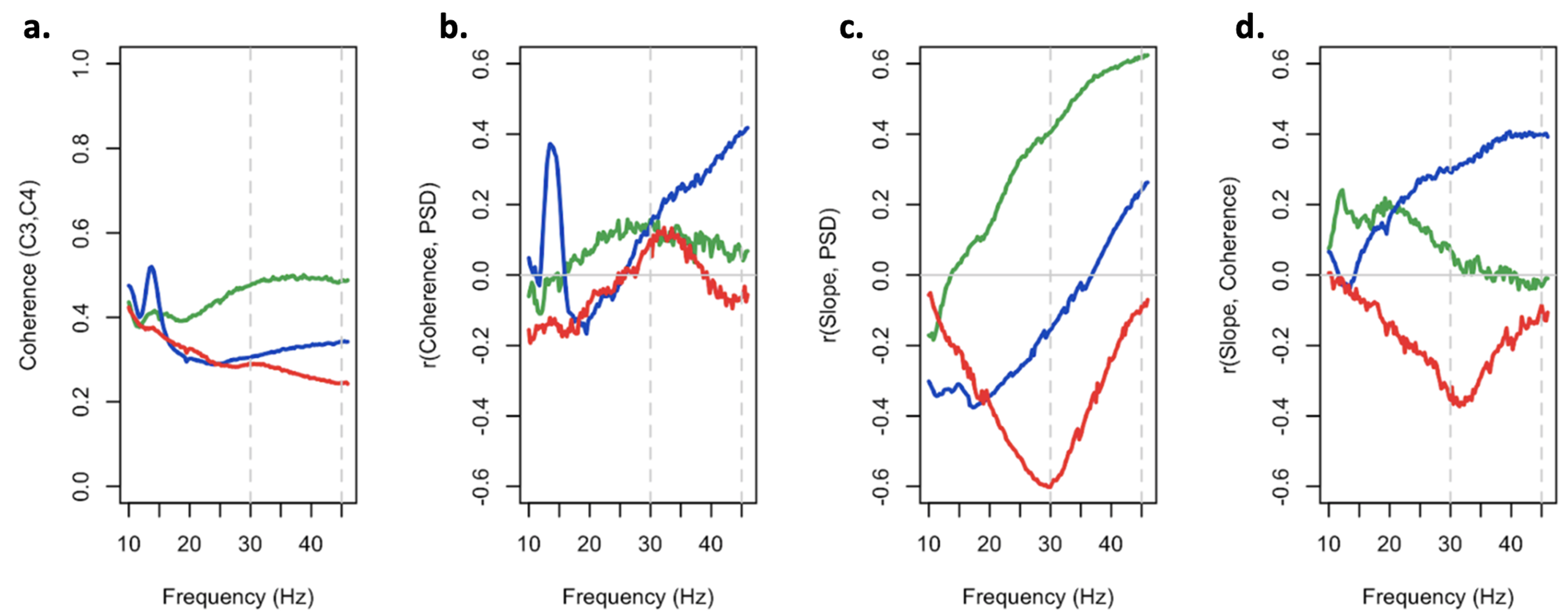

Supplement: Extended Data Figure 7-1 — State-specific relationships between inter-hemispheric coherence, power and spectral slope. Analyses based on CFS data only, all analyses based on the LM-referenced dataset. Green, blue and red indicate wake, NREM and REM respectively. Coherence was estimated using magnitude squared coherence, see Methods for details. a. Absolute coherence values were generally high (reflecting the common reference) but for beta and gamma frequencies we observed significantly lower coherence during REM compared to wake, with NREM showing an intermediate pattern. b. Coherence values were not independent of spectral power (here averaged across C3-LM and C4-LM), although we observed qualitatively different relationships between states. NREM exhibited a peak in power/coherence correlation in the sigma range (presumably driven by spindle activity), but also increased coherence/power correlation above 30 Hz. In contrast, during REM sleep there was an inflection point at 30 Hz, after which individual differences in coherence and power decoupled. c reproduces the slope/power correlation for CFS (as shown in Figure 7, but here based on the slope and power averaged over the two central channels). Finally, d shows the correlations between average slope (30-45 Hz) and coherence: as for slope and power, there were qualitatively different patterns between all three states. During REM, individuals with steeper slopes tended to show higher C3-C4 coherence, particularly around 30 Hz. In contrast, during NREM, individuals with steeper slopes tended to show lower coherence at higher (>20 Hz) frequencies, whereas for wake, individuals with steeper slopes tended to show lower coherence at slower (<20 Hz) frequencies. These results - alongside the prior results for the spectral power - underscore the types of qualitative state-dependent differences in measures related to the spectral slope, which appear to extend beyond simply differences in means. Download Figure 7-1, TIF file. [file enu-eN-NWR-0094-22-s11.tif]

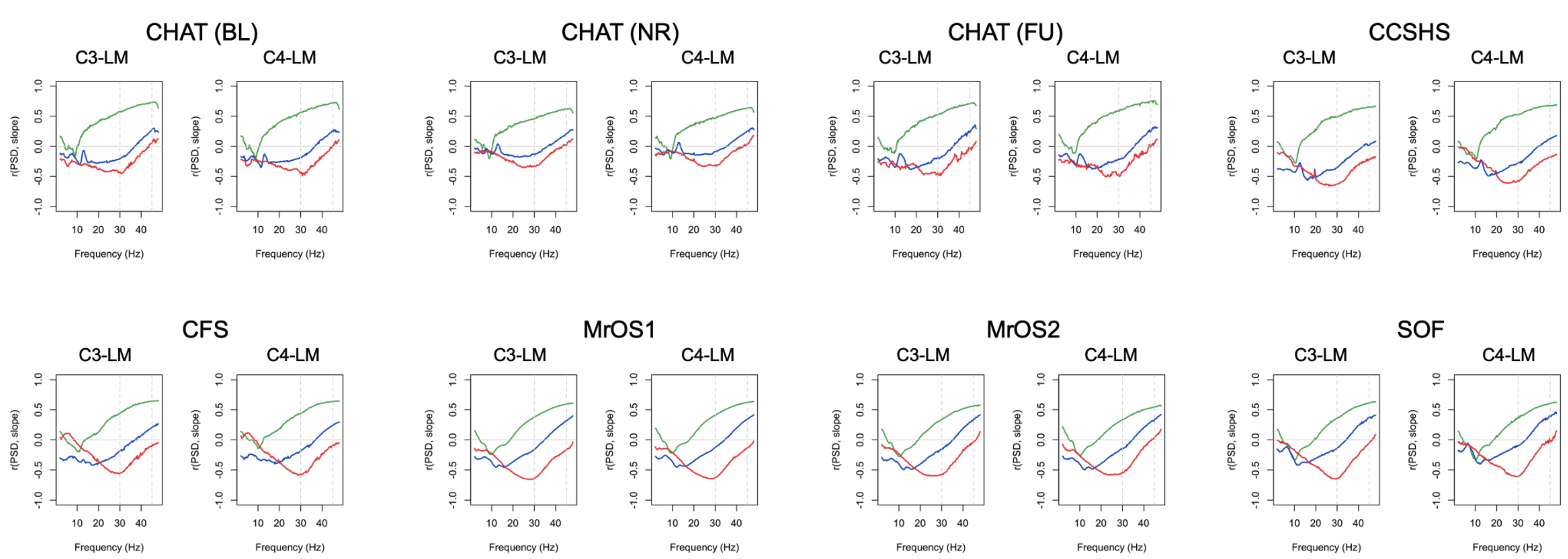

Supplement: Extended Data Figure 7-2 — Correlations between EEG spectral slope and power. All analyses based on the LM-referenced dataset. These Figures provide similar information as Figure 7 (top row) in the main text: here, all non-SHHS cohorts are plotted, results for both LM-referenced central channels are given; also, the x-axis extends < 10 Hz whereas Figure 7 excluded that portion of the power spectrum. Green, blue and red indicate wake, NREM and REM respectively. Download Figure 7-2, TIF file. [file enu-eN-NWR-0094-22-s12.tif]

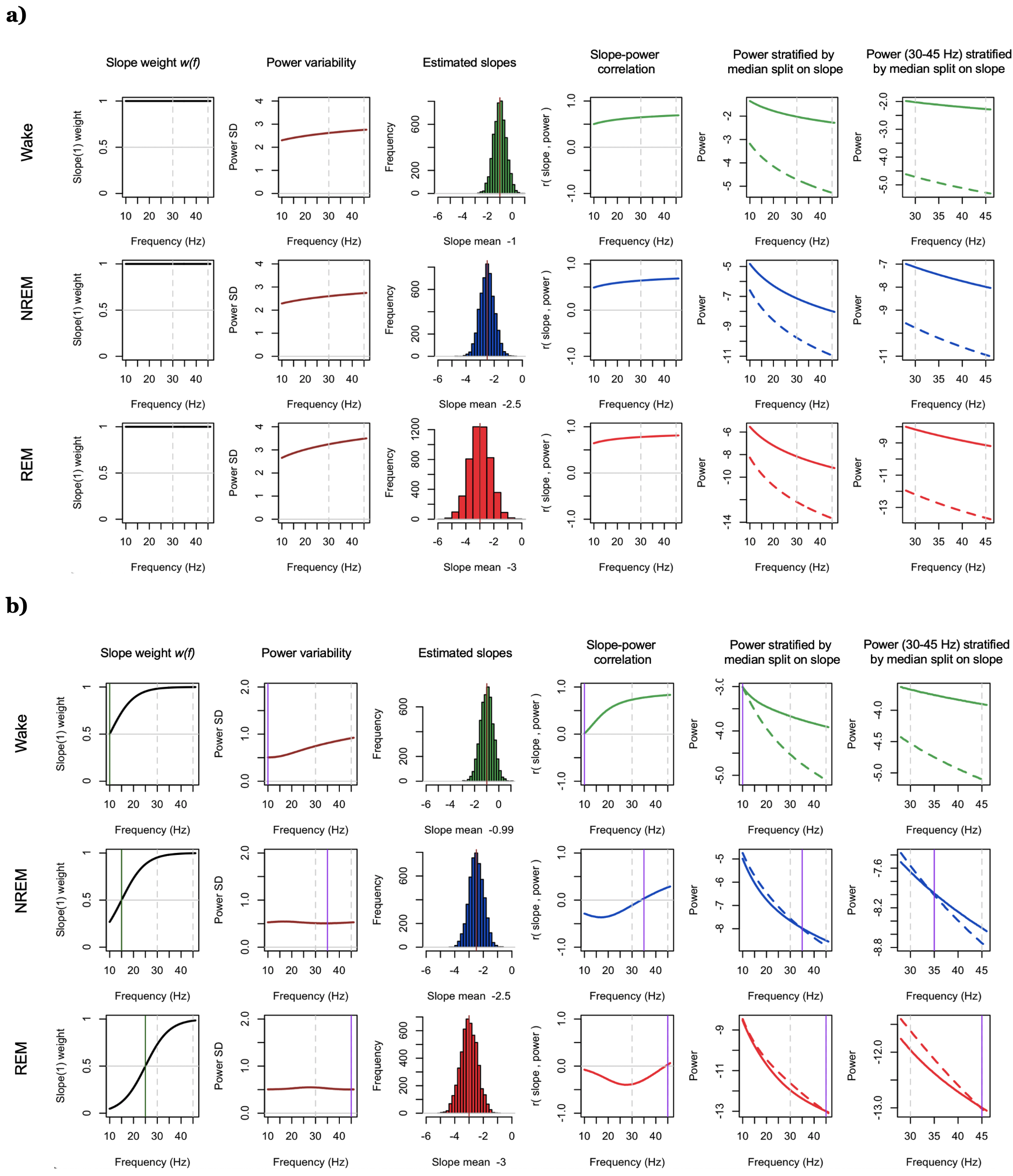

Supplement: Extended Data Figure 8-1 — Simulated power spectra with estimated power, spectral slopes, and their correlations. Based on N = 5,000 simulated spectra, derived statistics (columns 2 to 6) for a) the original model parameterization, assuming a strict power law model with mean α = 1, 2.5 and 3 for wake, NREM and REM respectively (and SDs of 0.5, 0.5 and 0.75, approximately following the observed between-individual estimates from Figure 6), and b) a revised model, with similar population parameters for slope means and variances but allowing different centers of rotation (fr = 10, 35 and 45 Hz for wake, NREM and REM respectively) and setting w(f) such that variation in α had less influence on the slope at lower frequencies. Green, blue and red indicate wake, NREM and REM respectively. See Methods for details. Download Figure 8-1, TIF file. [file enu-eN-NWR-0094-22-s13.tif]

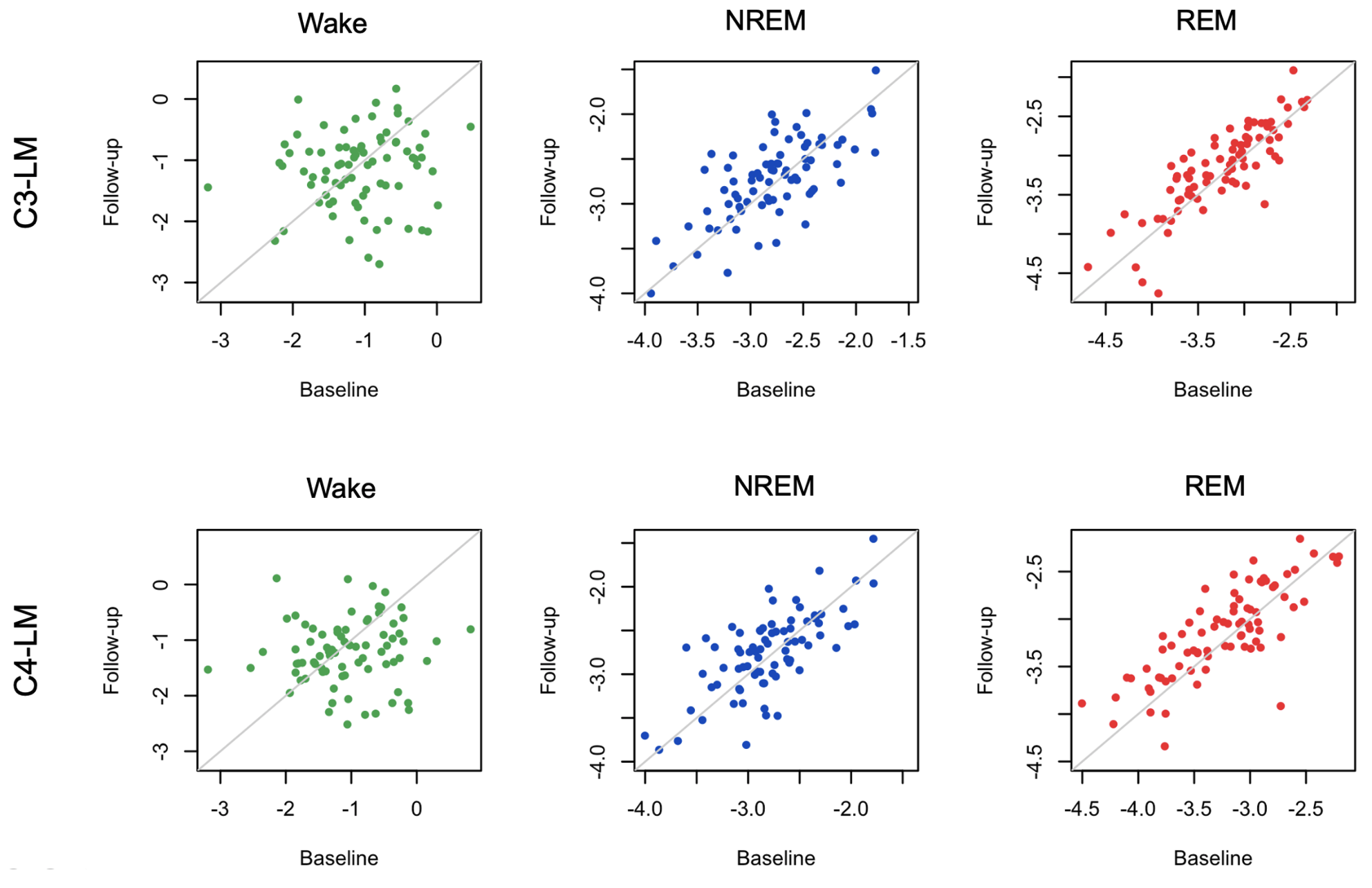

Supplement: Extended Data Figure 10-2 — Longitudinal analyses of the CHAT cohort. Based on the LM-referenced dataset, scatter-plots show the EEG spectral slope for baseline and follow-up CHAT (childhood) studies (N = 80 pairs post QC, ∼6 months interval) stratified by sleep state and channel (C3-LM and C4-LM). Green, blue and red indicate wake, NREM and REM respectively. See Figure 10-1 for statistical results. Download Figure 10-2, TIF file. [file enu-eN-NWR-0094-22-s14.tif]
