# Supplementary material for: Sources of Variation in the Spectral Slope of the Sleep EEG
Source: eNeuro. 2022 Sep 21;9(5):ENEURO.0094-22.2022. doi: 10.1523/ENEURO.0094-22.2022 (PMC9512622; doi:10.1523/ENEURO.0094-22.2022)
Supplement: Extended Data Figure 1-3 — Mean EEG slopes stratified by state and differences between slopes: CM-referenced dataset. See the Methods for details on the calculation of spectral slopes. Download Figure 1-3, DOC file. [file enu-eN-NWR-0094-22-s15.doc]

|  |  | *Mean slope* | | |  | *Mean difference* | |  | *t-statistic* | | |
| --- | --- | --- | --- | --- | --- | --- | --- | --- | --- | --- | --- |
| **Cohort** | **Channel** | **W** | **NR** | **R** |  | **NR - W** | **R - W** |  | **NR - W** | **R - NR** | **R - W** |
|  |  |  |  |  |  |  |  |  |  |  |  |
| CHAT | C3-M2 | -1.10 | -2.91 | -3.34 |  | -1.81 | -2.24 |  | -53.41 | -19.08 | -65.94 |
|  | C4-M1 | -1.15 | -2.98 | -3.43 |  | -1.83 | -2.27 |  | -51.99 | -18.62 | -64.39 |
|  | C3-C4 | -1.73 | -2.81 | -3.08 |  | -1.08 | -1.36 |  | -29.72 | -13.37 | -36.15 |
|  | M1-M2 | -0.96 | -3.08 | -3.75 |  | -2.12 | -2.80 |  | -49.06 | -21.37 | -64.09 |
|  |  |  |  |  |  |  |  |  |  |  |  |
| CCSHS | C3-M2 | -1.42 | -3.01 | -3.44 |  | -1.59 | -2.03 |  | -42.49 | -14.71 | -48.00 |
|  | C4-M1 | -1.41 | -3.17 | -3.61 |  | -1.76 | -2.19 |  | -40.04 | -13.53 | -46.16 |
|  | C3-C4 | -1.50 | -3.07 | -3.23 |  | -1.56 | -1.73 |  | -33.52 | -6.09 | -33.21 |
|  | M1-M2 | -1.34 | -2.70 | -3.29 |  | -1.36 | -1.95 |  | -24.12 | -15.58 | -32.08 |
|  |  |  |  |  |  |  |  |  |  |  |  |
| CFS | C3-M2 | -1.51 | -3.12 | -4.03 |  | -1.62 | -2.52 |  | -42.78 | -23.76 | -55.04 |
|  | C4-M1 | -1.58 | -3.31 | -4.20 |  | -1.73 | -2.62 |  | -39.82 | -22.97 | -56.67 |
|  | C3-C4 | -1.67 | -3.10 | -3.67 |  | -1.43 | -2.00 |  | -37.11 | -19.49 | -42.73 |
|  | M1-M2 | -1.45 | -3.12 | -3.85 |  | -1.67 | -2.40 |  | -28.39 | -17.65 | -37.07 |
|  |  |  |  |  |  |  |  |  |  |  |  |
| MrOS | C3-M2 | -0.77 | -1.87 | -2.87 |  | -1.10 | -2.11 |  | -75.63 | -68.93 | -120.59 |
|  | C4-M1 | -0.79 | -1.96 | -2.91 |  | -1.17 | -2.12 |  | -76.45 | -65.11 | -119.58 |
|  | C3-C4 | -1.09 | -2.42 | -2.88 |  | -1.33 | -1.79 |  | -78.59 | -42.75 | -93.89 |
|  | M1-M2 | -0.55 | -1.43 | -2.38 |  | -0.88 | -1.83 |  | -47.15 | -65.07 | -85.60 |
|  |  |  |  |  |  |  |  |  |  |  |  |
| SOF | C3-M2 | -0.97 | -2.32 | -3.24 |  | -1.35 | -2.27 |  | -30.81 | -22.01 | -46.88 |
|  | C4-M1 | -0.94 | -2.36 | -3.30 |  | -1.42 | -2.36 |  | -30.79 | -22.89 | -47.27 |
|  | C3-C4 | -1.52 | -2.77 | -3.09 |  | -1.26 | -1.57 |  | -25.50 | -9.74 | -28.24 |
|  | M1-M2 | -0.48 | -1.58 | -2.68 |  | -1.10 | -2.20 |  | -20.40 | -23.82 | -34.43 |
|  |  |  |  |  |  |  |  |  |  |  |  |

**Figure1-3. Mean EEG slopes stratified by state and differences between slopes: CM-referenced dataset.** See the **Methods** for details on the calculation of spectral slopes.
