# Supplementary material for: Sources of Variation in the Spectral Slope of the Sleep EEG
Source: eNeuro. 2022 Sep 21;9(5):ENEURO.0094-22.2022. doi: 10.1523/ENEURO.0094-22.2022 (PMC9512622; doi:10.1523/ENEURO.0094-22.2022)
Supplement: Extended Data Figure 2-1 — Mean EMG slopes by state and cohort. See Methods for details on the calculation of EMG spectral slopes. Download Figure 2-1, DOC file. [file enu-eN-NWR-0094-22-s16.doc]

|  |  | *Mean EMG slope* | | | | *t-test p-value (matched pairs)* | | |
| --- | --- | --- | --- | --- | --- | --- | --- | --- |
| **Cohort** |  | **W** | **NR** | **R** |  | **W - NR** | **NR - R** | **W - R** |
|  |  |  |  |  |  |  |  |  |
| CHAT(BL) |  | -0.597 | -1.453 | -1.231 |  | 1E-27 | 1E-03 | 1E-24 |
| CHAT(FU) |  | -0.489 | -1.087 | -1.528 |  | 2E-11 | 4E-07 | 4E-37 |
| CHAT(NR) |  | -0.509 | -1.044 | -1.374 |  | 2E-21 | 5E-09 | 4E-63 |
| CCSHS |  | -0.119 | -0.256 | -0.937 |  | 3E-03 | 5E-45 | 1E-67 |
| CFS |  | -0.182 | -0.493 | -0.929 |  | 2E-17 | 1E-30 | 3E-78 |
| SHHS1 |  | -0.105 | -0.185 | -0.403 |  | 3E-19 | 3E-184 | 9E-252 |
| SHHS2 |  | -0.083 | -0.138 | -0.331 |  | 2E-08 | 2E-110 | 3E-134 |
| MrOS1 |  | 0.041 | -0.247 | -0.469 |  | 1E-103 | 3E-78 | 1E-206 |
| MrOS2 |  | -0.067 | -0.333 | -0.500 |  | 2E-27 | 2E-19 | 3E-49 |
| SOF |  | 0.098 | -0.010 | -0.472 |  | 2E-03 | 2E-37 | 4E-40 |
|  |  |  |  |  |  |  |  |  |
| EMG slope (CM dataset) |  | -0.201 | -0.525 | -0.817 |  |  |  |  |

**Figure 2-1. Mean EMG slopes by state and cohort.**  See **Methods** for details on the calculation of EMG spectral slopes.
