# Supplementary material for: Sources of Variation in the Spectral Slope of the Sleep EEG
Source: eNeuro. 2022 Sep 21;9(5):ENEURO.0094-22.2022. doi: 10.1523/ENEURO.0094-22.2022 (PMC9512622; doi:10.1523/ENEURO.0094-22.2022)
Supplement: Extended Data Figure 2-2 — Associations between EEG and EMG spectral slopes in the CM-referenced dataset. Standardized regression coefficients and associated p-values from a regression of EEG slope on EMG controlling for age (up to 5th-order polynomials), sex, cohort, race, BMI, AHI and AI. All analyses were performed in the CM-referenced dataset. Download Figure 2-2, DOC file. [file enu-eN-NWR-0094-22-s17.doc]

|  |  | *b(EMG slope)* | | |  | *p(EMG slope)* | | |
| --- | --- | --- | --- | --- | --- | --- | --- | --- |
| **Channel** |  | **W** | **NR** | **R** |  | **W** | **NR** | **R** |
|  |  |  |  |  |  |  |  |  |
| C3-M2 |  | 0.040 | 0.078 | 0.074 |  | 9E-18 | 1E-40 | 4E-30 |
| C4-M1 |  | 0.029 | 0.058 | 0.058 |  | 1E-21 | 2E-42 | 2E-35 |
| C3-C4 |  | 0.072 | 0.064 | 0.079 |  | 7E-06 | 7E-06 | 4E-08 |
| M1-M2 |  | 0.184 | 0.251 | 0.330 |  | 1E-40 | 4E-89 | 2E-145 |
|  |  |  |  |  |  |  |  |  |

**Figure 2-2. Associations between EEG and EMG spectral slopes in the CM-referenced dataset.** Standardized regression coefficients and associated *p*-values from a regression of EEG slope on EMG controlling for age (up to 5th-order polynomials), sex, cohort, race, BMI, AHI and AI. All analyses were performed in the CM-referenced dataset.
