# Supplementary material for: Sources of Variation in the Spectral Slope of the Sleep EEG
Source: eNeuro. 2022 Sep 21;9(5):ENEURO.0094-22.2022. doi: 10.1523/ENEURO.0094-22.2022 (PMC9512622; doi:10.1523/ENEURO.0094-22.2022)
Supplement: Extended Data Figure 2-4 — EEG spectral slope associations with sex and BMI in the CM-referenced dataset. Coefficients and p-values from linear regression models of slope on sex and BMI, additionally controlling for age (and higher-order terms), race and cohort. Also see Figure 2-5. Download Figure 2-4, DOC file. [file enu-eN-NWR-0094-22-s18.doc]

|  |  |  | **Male sex** | | | | |  | **BMI** | | | | |
| --- | --- | --- | --- | --- | --- | --- | --- | --- | --- | --- | --- | --- | --- |
|  |  |  | **Excluding SHHS** | |  | **SHHS only** | |  | **Excluding SHHS** | |  | **SHHS only** | |
| **Stage** | **Channel** |  | *b(male)* | *p(male)* |  | *b(male)* | *p(male)* |  | *b(BMI)* | *p(BMI)* |  | *b(BMI)* | *p(BMI)* |
|  |  |  |  |  |  |  |  |  |  |  |  |  |  |
| W | C3-M2 |  | 0.124 | **0.0002** |  | 0.109 | **0.0001** |  | 0.0016 | 0.52 |  | 0.0083 | 0.006 |
| NR |  |  | 0.250 | **3E-11** |  | 0.289 | **3E-24** |  | -0.0081 | 0.004 |  | -0.0087 | 0.004 |
| R |  |  | 0.233 | **2E-07** |  | 0.415 | **4E-23** |  | -0.0008 | 0.82 |  | -0.0044 | 0.33 |
|  |  |  |  |  |  |  |  |  |  |  |  |  |  |
| W | C4-M1 |  | 0.088 | 0.008 |  | 0.085 | 0.20 |  | -0.0058 | 0.022 |  | 0.0079 | 0.27 |
| NR |  |  | 0.199 | **7E-07** |  | 0.310 | **3E-09** |  | -0.0192 | **3E-10** |  | -0.0117 | 0.035 |
| R |  |  | 0.217 | **2E-06** |  | 0.425 | **2E-12** |  | -0.0074 | 0.034 |  | -0.0070 | 0.28 |
|  |  |  |  |  |  |  |  |  |  |  |  |  |  |
| W | EMG |  | -0.041 | 0.047 |  | -0.014 | 0.33 |  | -0.0082 | **2E-07** |  | 0.0025 | 0.12 |
| NR |  |  | -0.145 | **1E-05** |  | -0.155 | **4E-15** |  | -0.0165 | **4E-11** |  | -0.0067 | 0.001 |
| R |  |  | -0.018 | 0.59 |  | -0.022 | 0.172 |  | -0.0140 | **2E-08** |  | -0.0046 | 0.008 |
|  |  |  |  |  |  |  |  |  |  |  |  |  |  |
| W | C3-C4 |  | 0.301 | **1E-11** |  |  |  |  | **0.0083** | 0.014 |  |  |  |
| NR |  |  | 0.260 | **3E-20** |  |  |  |  | **0.0085** | **6E-05** |  |  |  |
| R |  |  | 0.223 | **9E-10** |  |  |  |  | **0.0085** | **0.0020** |  |  |  |
|  |  |  |  |  |  |  |  |  |  |  |  |  |  |
| W | M1-M2 |  | -0.062 | **0.0073** |  |  |  |  | **-0.0106** | **2E-09** |  |  |  |
| NR |  |  | 0.032 | 0.537 |  |  |  |  | **-0.0402** | **9E-24** |  |  |  |
| R |  |  | 0.173 | **0.0028** |  |  |  |  | **-0.0298** | **2E-11** |  |  |  |
|  |  |  |  |  |  |  |  |  |  |  |  |  |  |
|  |  |  |  |  |  |  |  |  |  |  |  |  |  |

**Figure 2-4. EEG spectral slope associations with sex and BMI in the CM-referenced dataset.** Coefficients and *p*-values from linear regression models of slope on sex and BMI, additionally controlling for age (and higher-order terms), race and cohort. Also see **Figure 2-5**.
