# Supplementary material for: Sources of Variation in the Spectral Slope of the Sleep EEG
Source: eNeuro. 2022 Sep 21;9(5):ENEURO.0094-22.2022. doi: 10.1523/ENEURO.0094-22.2022 (PMC9512622; doi:10.1523/ENEURO.0094-22.2022)
Supplement: Extended Data Figure 2-6 — EEG spectral slope associations with sex and BMI in the LM-referenced dataset. Coefficients and p-values from linear regression models of slope on sex and BMI, additionally controlling for age (and higher-order terms), race and cohort. The SHHS was excluded from all LM-reference analyses. Download Figure 2-6, DOC file. [file enu-eN-NWR-0094-22-s19.doc]

|  |  |  | **Male sex** | |  | **BMI** | |
| --- | --- | --- | --- | --- | --- | --- | --- |
| **Channel** | **Stage** |  | *b(male)* | *p(male)* |  | *b(BMI)* | *p(BMI)* |
|  |  |  |  |  |  |  |  |
| C3-LM | W |  | **0.178** | 3E-08 |  | 0.004 | 0.072 |
|  | NR |  | **0.343** | 5E-25 |  | 0.005 | 0.07 |
|  | R |  | **0.318** | 1E-14 |  | **0.009** | **0.003** |
|  |  |  |  |  |  |  |  |
| C4-LM | W |  | **0.158** | 1E-06 |  | 0.002 | 0.46 |
|  | NR |  | **0.330** | 2E-23 |  | 0.002 | 0.42 |
|  | R |  | **0.333** | 1E-15 |  | **0.008** | **0.009** |
|  |  |  |  |  |  |  |  |
| C3-M2 | W |  | **0.140** | 4E-06 |  | 0.002 | 0.41 |
|  | NR |  | **0.285** | 4E-15 |  | -0.008 | **0.0061** |
|  | R |  | **0.286** | 2E-12 |  | 0.001 | 0.78 |
|  |  |  |  |  |  |  |  |
| C4-M1 | W |  | **0.109** | **0.0005** |  | -0.005 | 0.029 |
|  | NR |  | **0.224** | 9E-09 |  | -0.017 | **1E-08** |
|  | R |  | **0.259** | 6E-10 |  | -0.006 | 0.05 |
|  |  |  |  |  |  |  |  |
| C3-C4 | W |  | **0.323** | 1E-12 |  | **0.009** | **0.010** |
|  | NR |  | **0.276** | 1E-22 |  | **0.008** | 6E-05 |
|  | R |  | **0.217** | 9E-10 |  | **0.008** | **0.002** |
|  |  |  |  |  |  |  |  |
| M1-M2 | W |  | **-0.056** | **0.02** |  | **-0.012** | 3E-11 |
|  | NR |  | 0.016 | 0.77 |  | **-0.043** | 1E-24 |
|  | R |  | **0.155** | **0.0106** |  | **-0.036** | 1E-14 |
|  |  |  |  |  |  |  |  |
| EMG | W |  | -0.045 | 0.047 |  | **-0.007** | 1E-05 |
|  | NR |  | -0.169 | 5E-06 |  | **-0.015** | 1E-07 |
|  | R |  | -0.038 | 0.30 |  | **-0.017** | 1E-09 |
|  |  |  |  |  |  |  |  |

**Figure 2-6. EEG spectral slope associations with sex and BMI in the LM-referenced dataset.** Coefficients and *p*-values from linear regression models of slope on sex and BMI, additionally controlling for age (and higher-order terms), race and cohort. The SHHS was excluded from all LM-reference analyses.
