# Supplementary material for: Sources of Variation in the Spectral Slope of the Sleep EEG
Source: eNeuro. 2022 Sep 21;9(5):ENEURO.0094-22.2022. doi: 10.1523/ENEURO.0094-22.2022 (PMC9512622; doi:10.1523/ENEURO.0094-22.2022)
Supplement: Extended Data Figure 2-8 — Associations between EEG and EMG slopes in the LM-referenced dataset.Correlation coefficients and p-values for associations between EEG and EMG slopes in the LM-referenced dataset. Equivalent results are also reported for the CM-derived EEG slopes (in this same dataset). EEG-EMG correlations are attenuated comparing LM-derived to CM-derived estimates (albeit still significantly larger than zero). Similar results were obtained when controlling for age, sex, cohort and other covariates (see legend for Figure 2-2 for details; note: Figure 2-2 shows standardized regression coefficients from the adjusted model, and so the CM results are not directly comparable to the correlation coefficient presented here). Download Figure 2-8, DOC file. [file enu-eN-NWR-0094-22-s20.doc]

|  |  | *r(EMG slope)* | | |  | *p(EMG slope)* | | |
| --- | --- | --- | --- | --- | --- | --- | --- | --- |
| **Channel** |  | **W** | **NR** | **R** |  | **W** | **NR** | **R** |
|  |  |  |  |  |  |  |  |  |
| C3-LM |  | 0.114 | 0.092 | 0.045 |  | 6E-10 | 2E-08 | 2E-02 |
| C4-LM |  | 0.109 | 0.104 | 0.067 |  | 2E-09 | 1E-10 | 2E-04 |
|  |  |  |  |  |  |  |  |  |
| C3-M2 |  | 0.153 | 0.220 | 0.190 |  | < 1E-15 | < 1E-15 | < 1E-15 |
| C4-M1 |  | 0.154 | 0.245 | 0.217 |  | < 1E-15 | < 1E-15 | < 1E-15 |
|  |  |  |  |  |  |  |  |  |

**Figure 2-8. Associations between EEG and EMG slopes in the LM-referenced dataset.** Correlation coefficients and *p*-values for associations between EEG and EMG slopes in the LM-referenced dataset. Equivalent results are also reported for the CM-derived EEG slopes (in this same dataset). EEG-EMG correlations are attenuated comparing LM-derived to CM-derived estimates (albeit still significantly larger than zero). Similar results were obtained when controlling for age, sex, cohort and other covariates (see legend for **Figure 2-2** for details; note: **Figure 2-2** shows standardized regression coefficients from the adjusted model, and so the CM results are not directly comparable to the correlation coefficient presented here).
