# Supplementary material for: Sources of Variation in the Spectral Slope of the Sleep EEG
Source: eNeuro. 2022 Sep 21;9(5):ENEURO.0094-22.2022. doi: 10.1523/ENEURO.0094-22.2022 (PMC9512622; doi:10.1523/ENEURO.0094-22.2022)
Supplement: Extended Data Figure 3-2 — Mean EEG slopes and state differences in the LM-referenced dataset. Comparable state-specific slope means, and tests of state differences as shown in Figure 1-3 (for the CM-dataset/channels), but here for the LM-derived slopes. Download Figure 3-2, DOC file. [file enu-eN-NWR-0094-22-s21.doc]

|  |  |  | Mean EEG spectral slope | | | | |  | Within-individual, between stage t-statistics | | | | | |
| --- | --- | --- | --- | --- | --- | --- | --- | --- | --- | --- | --- | --- | --- | --- |
| **Cohort** | **Channel** |  | **W** | **N1** | **N2** | **N3** | **R** |  | **NR - W** | **R - NR** | **R - W** |  | **N2 - N1** | **N2 - N3** |
|  |  |  |  |  |  |  |  |  |  |  |  |  |  |  |
| CHAT (BL+NR) | C3-LM |  | -1.10 | -2.58 | -2.81 | -2.54 | -3.20 |  | -54.40 | -18.97 | -65.45 |  | -8.81 | -13.14 |
|  | C4-LM |  | -1.12 | -2.58 | -2.82 | -2.56 | -3.21 |  | -52.59 | -18.64 | -64.98 |  | -9.19 | -12.48 |
|  |  |  |  |  |  |  |  |  |  |  |  |  |  |  |
| CCSHS | C3-LM |  | -1.38 | -2.69 | -3.00 | -2.81 | -3.40 |  | -44.10 | -13.03 | -47.94 |  | -6.11 | -8.90 |
|  | C4-LM |  | -1.38 | -2.69 | -3.05 | -2.87 | -3.44 |  | -42.32 | -13.14 | -46.44 |  | -7.13 | -8.58 |
|  |  |  |  |  |  |  |  |  |  |  |  |  |  |  |
| CFS | C3-LM |  | -1.38 | -2.72 | -2.84 | -2.65 | -3.77 |  | -41.99 | -23.76 | -54.28 |  | -0.71 | -10.27 |
|  | C4-LM |  | -1.40 | -2.78 | -2.91 | -2.72 | -3.85 |  | -42.21 | -23.58 | -55.32 |  | -0.86 | -9.31 |
|  |  |  |  |  |  |  |  |  |  |  |  |  |  |  |
| MrOS | C3-LM |  | -0.73 | -1.77 | -1.83 | -1.61 | -2.81 |  | -77.92 | -69.05 | -119.00 |  | 2.37 | -24.18 |
|  | C4-LM |  | -0.73 | -1.77 | -1.84 | -1.63 | -2.80 |  | -78.99 | -68.43 | -119.30 |  | 1.62 | -22.80 |
|  |  |  |  |  |  |  |  |  |  |  |  |  |  |  |
| SOF | C3-LM |  | -0.96 | -2.22 | -2.35 | -1.99 | -3.24 |  | -32.52 | -21.10 | -44.61 |  | 0.21 | -14.03 |
|  | C4-LM |  | -0.93 | -2.16 | -2.36 | -1.99 | -3.24 |  | -31.23 | -22.07 | -43.48 |  | -0.35 | -13.57 |
|  |  |  |  |  |  |  |  |  |  |  |  |  |  |  |

**Figure 3-2. Mean EEG slopes and state differences in the LM-referenced dataset.** Comparable state-specific slope means, and tests of state differences as shown in **Figure 1-3** (for the CM-dataset/channels), but here for the LM-derived slopes.
