# Supplementary material for: Sources of Variation in the Spectral Slope of the Sleep EEG
Source: eNeuro. 2022 Sep 21;9(5):ENEURO.0094-22.2022. doi: 10.1523/ENEURO.0094-22.2022 (PMC9512622; doi:10.1523/ENEURO.0094-22.2022)
Supplement: Extended Data Figure 3-3 — Cross-state correlations in EEG spectral slopes. All statistics based on the LM-reference datasets (slopes for C3-LM and C4-LM). Correlations stratified by cohort, with outliers (+/- 3 SD units) removed. All correlations are significantly greater than 0 (p < 10-10). Download Figure 3-3, DOC file. [file enu-eN-NWR-0094-22-s22.doc]

|  |  |  | *EEG slope cross-stage correlations* | | |
| --- | --- | --- | --- | --- | --- |
| **Cohort** | **Channel** |  | ***r( NR , R )*** | ***r( R , W )*** | ***r( NR , W )*** |
|  |  |  |  |  |  |
| CCSHS | C3-LM |  | 0.53 | 0.24 | 0.32 |
|  | C4-LM |  | 0.55 | 0.22 | 0.31 |
|  |  |  |  |  |  |
| CFS | C3-LM |  | 0.51 | 0.43 | 0.46 |
|  | C4-LM |  | 0.51 | 0.41 | 0.44 |
|  |  |  |  |  |  |
| CHAT(BL) | C3-LM |  | 0.62 | 0.28 | 0.28 |
|  | C4-LM |  | 0.58 | 0.26 | 0.25 |
|  |  |  |  |  |  |
| CHAT(NR) | C3-LM |  | 0.67 | 0.21 | 0.24 |
|  | C4-LM |  | 0.64 | 0.19 | 0.21 |
|  |  |  |  |  |  |
| CHAT(FU) | C3-LM |  | 0.58 | 0.41 | 0.43 |
|  | C4-LM |  | 0.56 | 0.35 | 0.41 |
|  |  |  |  |  |  |
| MrOS1 | C3-LM |  | 0.65 | 0.47 | 0.52 |
|  | C4-LM |  | 0.64 | 0.47 | 0.50 |
|  |  |  |  |  |  |
| MrOS2 | C3-LM |  | 0.73 | 0.55 | 0.58 |
|  | C4-LM |  | 0.76 | 0.55 | 0.57 |
|  |  |  |  |  |  |
| SOF | C3-LM |  | 0.61 | 0.44 | 0.52 |
|  | C4-LM |  | 0.68 | 0.45 | 0.51 |

**Figure 3-3. Cross-state correlations in EEG spectral slopes.** All statistics based on the LM-reference datasets (slopes for C3-LM and C4-LM). Correlations stratified by cohort, with outliers (+/- 3 SD units) removed. All correlations are significantly greater than 0 (*p* < 10^-10^).
