# Supplementary material for: Sources of Variation in the Spectral Slope of the Sleep EEG
Source: eNeuro. 2022 Sep 21;9(5):ENEURO.0094-22.2022. doi: 10.1523/ENEURO.0094-22.2022 (PMC9512622; doi:10.1523/ENEURO.0094-22.2022)
Supplement: Extended Data Figure 10-1 — Test-retest correlations and mean differences in the EEG spectral slope (MrOS and CHAT). Also see Figure 10 (MrOS) and Figure 10-2 (CHAT) for plots of test-retest EEG slope distributions. Download Figure 10-1, DOC file. [file enu-eN-NWR-0094-22-s23.doc]

|  |  |  |  | *Test-retest correlations* | |  | *Mean EEG slopes* | |  | *Test-retest mean differences* | |
| --- | --- | --- | --- | --- | --- | --- | --- | --- | --- | --- | --- |
| **Cohort** | **Channel** | **Stage(s)** |  | **r** | **p-value** |  | **Baseline** | **Follow-up** |  | **Difference** | **p-value** |
|  |  |  |  |  |  |  |  |  |  |  |  |
| MrOS | C3-LM | W |  | 0.55 | 8E-48 |  | -0.7 | -0.75 |  | -0.04 | 0.08 |
| (n=610 pairs, post QC) |  | NR |  | 0.69 | 3E-87 |  | -1.84 | -1.67 |  | 0.17 | 1E-15 |
| ~6 years |  | R |  | 0.80 | 8E-138 |  | -2.89 | -2.53 |  | 0.37 | 4E-50 |
|  |  |  |  |  |  |  |  |  |  |  |  |
|  | C4-LM | W |  | 0.51 | 7E-41 |  | -0.709 | -0.709 |  | 0.00 | 0.98 |
|  |  | NR |  | 0.63 | 5E-68 |  | -1.84 | -1.53 |  | 0.32 | 4E-42 |
|  |  | R |  | 0.75 | 6E-108 |  | -2.86 | -2.3 |  | 0.57 | 5E-87 |
|  |  |  |  |  |  |  |  |  |  |  |  |
|  | C3-LM | R - NR |  | 0.67 | 2E-78 |  | -1.04 | -0.853 |  | 0.20 | 9E-20 |
|  |  | R - W |  | 0.62 | 9E-64 |  | -2.17 | -1.76 |  | 0.41 | 1E-39 |
|  |  | NR - W |  | 0.49 | 2E-37 |  | -1.13 | -0.908 |  | 0.22 | 3E-16 |
|  |  |  |  |  |  |  |  |  |  |  |  |
|  | C4-LM | R - NR |  | 0.67 | 3E-80 |  | -1.01 | -0.778 |  | 0.25 | 2E-31 |
|  |  | R - W |  | 0.55 | 1E-48 |  | -2.14 | -1.58 |  | 0.57 | 3E-63 |
|  |  | NR - W |  | 0.43 | 1E-28 |  | -1.13 | -0.801 |  | 0.32 | 4E-30 |
|  |  |  |  |  |  |  |  |  |  |  |  |
| **Cohort** | **Channel** | **Stage(s)** |  | **r** | **p-value** |  | **Baseline** | **Follow-up** |  | **Difference** | **p-value** |
|  |  |  |  |  |  |  |  |  |  |  |  |
| CHAT | C3-LM | W |  | 0.10 | 0.38 |  | -1.08 | -1.15 |  | -0.08 | 0.42 |
| (n=80 pairs, post QC) |  | N2 |  | 0.69 | 1E-12 |  | -2.79 | -2.72 |  | 0.07 | 0.1 |
| ~6 months |  | R |  | 0.84 | 9E-23 |  | -3.26 | -3.16 |  | 0.09 | 0.006 |
|  |  |  |  |  |  |  |  |  |  |  |  |
|  | C4-LM | W |  | 0.08 | 0.46 |  | -1.06 | -1.21 |  | -0.16 | 0.1 |
|  |  | N2 |  | 0.71 | 4E-13 |  | -2.81 | -2.75 |  | 0.05 | 0.2 |
|  |  | R |  | 0.79 | 4E-18 |  | -3.26 | -3.14 |  | 0.10 | 0.004 |
|  |  |  |  |  |  |  |  |  |  |  |  |
|  | C3-LM | R - NR |  | 0.71 | 3E-13 |  | -0.456 | -0.431 |  | 0.03 | 0.52 |
|  |  | R - W |  | 0.28 | 0.01 |  | -2.17 | -2.02 |  | 0.17 | 0.07 |
|  |  | NR - W |  | 0.20 | 0.08 |  | -1.72 | -1.57 |  | 0.16 | 0.08 |
|  |  |  |  |  |  |  |  |  |  |  |  |
|  | C4-LM | R - NR |  | 0.58 | 2E-08 |  | -0.441 | -0.371 |  | 0.05 | 0.21 |
|  |  | R - W |  | 0.26 | 0.021 |  | -2.19 | -1.92 |  | 0.27 | 0.005 |
|  |  | NR - W |  | 0.23 | 0.041 |  | -1.76 | -1.55 |  | 0.22 | 0.02 |
|  |  |  |  |  |  |  |  |  |  |  |  |

**Figure 10-1. Test-retest correlations and mean differences in the EEG spectral slope (MrOS and CHAT).** Also see **Figure 10** (MrOS) and **Figure 10-2** (CHAT) for plots of test-retest EEG slope distributions.
