# Supplementary material for: Sources of Variation in the Spectral Slope of the Sleep EEG
Source: eNeuro. 2022 Sep 21;9(5):ENEURO.0094-22.2022. doi: 10.1523/ENEURO.0094-22.2022 (PMC9512622; doi:10.1523/ENEURO.0094-22.2022)
Supplement: Extended Data Figure 10-3 — Cross-sectional analysis of age-related flattening of the spectral slope. All statistics are based on the LM-reference datasets, using a multiple linear regression model of EEG slope (here average of C3-LM and C4-LM) on age (linear) plus covariates, performed within cohort. CCSHS was excluded as there was effectively no variation in age (most participants were either 17 or 18 years of age). Similar patterns of results were obtained for analyses of each individual LM-referenced channel. Download Figure 10-3, DOC file. [file enu-eN-NWR-0094-22-s24.doc]

|  |  | **Wake** | | |  | **NR** | | |  | **R** | | |
| --- | --- | --- | --- | --- | --- | --- | --- | --- | --- | --- | --- | --- |
| **Cohort** |  | ***Mean*** | ***b(age)*** | ***p(age)*** |  | ***Mean*** | ***b(age)*** | ***p(age)*** |  | ***Mean*** | ***b(age)*** | ***p(age)*** |
|  |  |  |  |  |  |  |  |  |  |  |  |  |
| CHAT(BL) |  | -1.11 | 0.071 | 0.058 |  | -2.81 | **0.158** | **6E-08** |  | -3.23 | **0.134** | **3E-06** |
| CHAT(NR) |  | -1.07 | **0.081** | **0.002** |  | -2.82 | **0.135** | **3E-08** |  | -3.17 | **0.117** | **4E-07** |
| CFS |  | -1.37 | -0.001 | 0.68 |  | -2.87 | **0.005** | **0.006** |  | -3.85 | **-0.009** | **1E-04** |
| MrOS1 |  | -0.71 | 0.001 | 0.69 |  | -1.82 | -0.002 | 0.53 |  | -2.85 | **0.009** | **0.0081** |
| SOF |  | -0.94 | -0.003 | 0.82 |  | -2.34 | -0.002 | 0.88 |  | -3.25 | 0.017 | 0.32 |
|  |  |  |  |  |  |  |  |  |  |  |  |  |
|  |  | **NR-W** | | |  | **R-NR** | | |  | **R-W** | | |
| **Cohort** |  | ***Mean*** | ***b(age)*** | ***p(age)*** |  | ***Mean*** | ***b(age)*** | ***p(age)*** |  | ***Mean*** | ***b(age)*** | ***p(age)*** |
|  |  |  |  |  |  |  |  |  |  |  |  |  |
| CHAT(BL) |  | -1.70 | **0.091** | **0.033** |  | -0.40 | -0.024 | 0.39 |  | -2.10 | 0.049 | 0.27 |
| CHAT(NR) |  | -1.75 | 0.054 | 0.094 |  | -0.35 | -0.018 | 0.38 |  | -2.11 | 0.035 | 0.259 |
| CFS |  | -1.49 | **0.006** | **0.0025** |  | -0.99 | **-0.015** | **8E-13** |  | -2.49 | **-0.009** | **0.00028** |
| MrOS1 |  | -1.11 | -0.003 | 0.18 |  | -1.02 | **0.011** | **0.00004** |  | -2.12 | **0.008** | **0.012** |
| SOF |  | -1.41 | 0.004 | 0.77 |  | -0.89 | 0.025 | 0.068 |  | -2.33 | 0.026 | 0.142 |
|  |  |  |  |  |  |  |  |  |  |  |  |  |

**Figure 10-3. Cross-sectional analysis of age-related flattening of the spectral slope.** All statistics are based on the LM-reference datasets, using a multiple linear regression model of EEG slope (here average of C3-LM and C4-LM) on age (linear) plus covariates, performed within cohort. CCSHS was excluded as there was effectively no variation in age (most participants were either 17 or 18 years of age). Similar patterns of results were obtained for analyses of each individual LM-referenced channel.
